# A randomised controlled trial of Standard Of Care versus RadioAblaTion in Early Stage HepatoCellular Carcinoma (SOCRATES HCC)

**DOI:** 10.1186/s12885-024-12504-2

**Published:** 2024-07-08

**Authors:** Alan Wigg, Jonathan Tibballs, Richard Woodman, Katherine Stuart, Hien Le, Stuart K. Roberts, John K. Olynyk, Simone I. Strasser, Michael Wallace, Jarad Martin, Annette Haworth, Nicholas Hardcastle, Kee Fong Loo, Colin Tang, Yoo Young Lee, Julie Chu, Richard De Abreu Lourenco, Adam Koukourou, Diederick De Boo, Kate McLean, Jackie Buck, Rohit Sawhney, Amanda Nicoll, Anouk Dev, Marnie Wood, Alicia Braund, Martin Weltman, Richard Khor, Miriam Levy, Tim Wang, Michael Potter, James Haridy, Ashok Raj, Oliver Duncan, Amany Zekry, Natalie Collier, James O’Beirne, Catherine Holliday, Yuvnik Trada, Jaw Tronidjaja, Jacob George, David Pryor

**Affiliations:** 1 Southern Adelaide Local Health Network, Adelaide, Australia; 2 Flinders University of South Australia, Adelaide, Australia; 3 Sir Charles Gardiner Hospital, Perth, Australia; 4 Princess Alexandra Hospital, Brisbane, Australia; 5 Central Adelaide Local Health Network, Adelaide, Australia; 6 University of South Australia, Adelaide, Australia; 7 The Alfred Hospital, Melbourne, Australia; 8 Fiona Stanley Hospital, Perth, Australia; 9 Royal Prince Alfred Hospital, Sydney, Australia; 10 University of Sydney, Sydney, Australia; 11 University of Newcastle, Newcastle, England; 12 Peter MacCallum Cancer Centre, Melbourne, Australia; 13 Sir Peter MacCallum Department of Oncology, University of Melbourne, Melbourne, Australia; 14 University of Technology Sydney, Sydney, Australia; 15 Monash Health, Melbourne, Australia; 16 Trans-Tasman Radiation Oncology Group, Waratah, Australia; 17 Eastern Health, Melbourne, Australia; 18 Royal Brisbane and Women’s Hospital, Brisbane, Australia; 19 Gold Coast University Hospital, Gold Coast, Australia; 20 Nepean Hospital, Sydney, Australia; 21 Austin Health, Melbourne, Australia; 22 Liverpool Hospital, Sydney, Australia; 23 Westmead Hospital, Sydney, Australia; 24 John Hunter Hospital, Newcastle, Australia; 25 Royal Melbourne Hospital, Melbourne, Australia; 26 St George Hospital, Sydney, Australia; 27 Wollongong Hospital, Wollongong, Australia; 28 Sunshine Coast University Hospital, Sunshine Coast, Australia; 29 Centre for Community-Driven Research, Sydney, Australia; 30 Calvary Mater, Newcastle, Australia; 31 Storr Liver Centre, Westmead Institute for Medical Research, Westmead Hospital, Sydney, Australia

**Keywords:** Early-stage hepatocellular carcinoma, Stereotactic ablative radiotherapy, SABR, SBRT, Thermal ablation, Transarterial therapies, Treatment stage migration

## Abstract

**Background:**

Therapeutic options for early-stage hepatocellular carcinoma (HCC) in individual patients can be limited by tumor and location, liver dysfunction and comorbidities. Many patients with early-stage HCC do not receive curative-intent therapies. Stereotactic ablative body radiotherapy (SABR) has emerged as an effective, non-invasive HCC treatment option, however, randomized evidence for SABR in the first line setting is lacking.

**Methods:**

Trans-Tasman Radiation Oncology Group (TROG) 21.07 SOCRATES-HCC is a phase II, prospective, randomised trial comparing SABR to other current standard of care therapies for patients with a solitary HCC ≤ 8 cm, ineligible for surgical resection or transplantation. The study is divided into 2 cohorts. Cohort 1 will compromise 118 patients with tumors ≤ 3 cm eligible for thermal ablation randomly assigned (1:1 ratio) to thermal ablation or SABR. Cohort 2 will comprise 100 patients with tumors > 3 cm up to 8 cm in size, or tumors ≤ 3 cm ineligible for thermal ablation, randomly assigned (1:1 ratio) to SABR or best other standard of care therapy including transarterial therapies. The primary objective is to determine whether SABR results in superior freedom from local progression (FFLP) at 2 years compared to thermal ablation in cohort 1 and compared to best standard of care therapy in cohort 2. Secondary endpoints include progression free survival, overall survival, adverse events, patient reported outcomes and health economic analyses.

**Discussion:**

The SOCRATES-HCC study will provide the first randomized, multicentre evaluation of the efficacy, safety and cost effectiveness of SABR versus other standard of care therapies in the first line treatment of unresectable, early-stage HCC. It is a broad, multicentre collaboration between hepatology, interventional radiology and radiation oncology groups around Australia, coordinated by TROG Cancer Research.

**Trial registration:**

anzctr.org.au, ACTRN12621001444875, registered 21 October 2021.

**Supplementary Information:**

The online version contains supplementary material available at 10.1186/s12885-024-12504-2.

## Background

Liver cancer is the second leading cause of cancer-related death globally, mainly accounted for by hepatocellular carcinoma (HCC) [1]. It is the only low survival cancer with a rapidly rising incidence. Survival rates for advanced disease are poor and treatment options for early-stage disease can be limited. The treatment strategy in patients with HCC is based on tumour stage, underlying liver function and performance status [2]. The Barcelona Clinic Liver Cancer (BCLC 0/A) staging system is commonly used to guide prognostication and treatment of patients with HCC [3]. For early-stage HCC (BCLC stage 0/A), curative surgical therapies such as liver transplantation and liver resection are recommended to achieve the best survival outcomes, however, less than 30% of patients are candidates for surgery upon diagnosis [2–5].

Thermal ablation using microwave ablation (MWA) or radiofrequency ablation (RFA) is the current standard curative therapy for non-surgical candidates with early-stage HCC ≤ 3 cm [6]. Larger tumour size (> 3 cm) and certain anatomical locations (subphrenic, subcapsular, in close proximity to large vessels or central biliary structures) have been associated with higher local failure rates and increased complication rates [7–9]. Furthermore, many patients with a single HCC nodule are not suitable for thermal ablation and undergo so called “treatment stage migration” [10, 11]. For these patients the current standard of care migrates to transarterial therapies traditionally considered as non-curative, such as transarterial chemoembolization (TACE) or transarterial radioembolization (TARE).

Stereotactic ablative body radiotherapy (SABR) has emerged as a highly effective, non-invasive treatment option for early-stage HCC and has the advantage of being able to treat larger tumours and those abutting the diaphragm, liver capsule, major blood vessels and bile ducts [9, 12, 13]. However, its use is not uniformly supported in clinical guidelines due to a lack of randomized evidence. Non-randomized studies and systematic reviews have reported high local control rates for SABR (> 90% at 3 years), with low rates of grade ≥ 3 toxicities (< 5%), and similar survival outcomes to thermal ablation [13–16]. One randomized study has demonstrated non-inferior 2-year local progression free survival for proton beam radiotherapy compared to RFA for recurrent HCC, however, no study has evaluated SABR against thermal ablation in the first line setting [17]. The Dutch TRENDY study aimed to evaluate SABR against TACE in a treatment stage migration cohort (predominantly solitary HCC not suitable for resection or thermal ablation), however, this study closed due to slow accrual [18]. For the 28 evaluable patients a post-hoc analysis reported 2-year local control of 100% for SBRT and 43.6% for TACE (*p* = 0.019), a promising finding that warrants investigation in a larger study.

The Trans-Tasman Radiation Oncology Group (TROG) 21.07 SOCRATES-HCC study aims to address this evidence gap in non-surgical, early-stage HCC. For patients with tumours ≤ 3 cm and deemed suitable for percutaneous thermal ablation (Cohort 1), SOCRATES-HCC aims to prospectively evaluate the efficacy, toxicity, health related quality of life and cost-effectiveness of SABR compared to modern thermal ablation techniques. For the population of patients with HCC > 3 cm or HCC ≤ 8 cm but ineligible for ablation (Cohort 2), SOCRATES-HCC will evaluate the efficacy of SABR compared to modern transarterial therapies (including the option of combined TACE-ablation) in the first line setting.

## Methods/design

### Study design

**Fig. 1:**
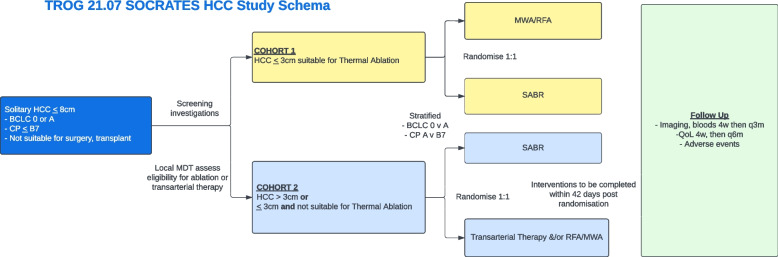
TROG 21.07 SOCRATES HCC study schema

TROG 21.07 SOCRATES-HCC is a phase II, prospective, randomised, parallel and open-label, multi-institutional superiority trial comparing SABR to other current standard of care therapies for patients with newly diagnosed early stage (BCLC 0/A), solitary ≤ 8 cm nodule HCC, ineligible for surgical resection or transplantation. The trial schema is represented in Fig. 1.

Patients with tumours ≤ 3 cm and eligible for percutaneous thermal ablation comprise cohort 1 and will be randomly assigned (1:1 ratio) to percutaneous thermal ablation (MWA or RFA) or SABR. Patients with tumours ≤ 3 cm assessed as ineligible for thermal ablation or patients with tumours > 3 cm up to 8 cm in size comprise cohort 2 and will be randomly assigned (1:1 ratio) to SABR or best other standard of care therapy comprising any transarterial therapy (TACE or TARE). For patients in cohort 2 with tumours > 3 cm considered suitable for percutaneous thermal ablation or combined procedures, thermal ablation is allowed.

### Study objectives and hypotheses

The primary hypothesis of this study is that SABR will result in higher rates of freedom from local progression (FFLP) at 2 years compared to percutaneous thermal ablation in cohort 1 and compared to best standard of care therapy in cohort 2. The primary endpoint is 2-year FFLP according to the modified Response Evaluation Criteria in Solid Tumours (mRECIST) [19]. To account for differences in arterial enhancement patterns following radiation-based therapies (SABR and TARE), progression on RECIST v1.1 criteria will also be counted as a progression event [20]. Key secondary endpoints include progression (at any site) free survival (PFS), overall survival (OS), adverse events (CTCAE v5), patient reported outcomes (including EORTC QLQ-C30, QLQ-HCC18), a qualitative assessment of treatment experience and health economic analyses.

#### Rationale for choice of primary endpoint

The aim of any local therapy is to maximise local control whilst minimising toxicity and any impact on functional reserve. This study is powered to detect a clinically relevant improvement in the primary endpoint of 2-year FFLP. Whilst overall survival is considered the most robust efficacy endpoint for an intervention it can be confounded by the multitude of effective downstream therapies available and their subsequent effects on progression of tumour and underlying liver dysfunction [21, 22]. This is particularly the case when evaluating therapies in early stage HCC. Demonstration of a significant improvement in local control within the context of a randomised trial would provide evidence to support the inclusion of SABR as an initial treatment option for single nodule HCC (BCLC-O/A).

#### Study setting

SOCRATES-HCC will be conducted across 21 Australian centres including both metropolitan and regional tertiary care public hospitals. The study is a collaboration between hepatologists, radiation oncologists and interventional radiologists and is coordinated by TROG (trial sponsor) and endorsed by the Gastroenterological Society of Australia (GESA), the Abdominal Radiology Group of Australia and New Zealand (ARGANZ) and the Australian Gastrointestinal Trials Group (AGITG).

#### Characteristics of participants

The target population includes any newly diagnosed, early stage (BCLC 0/A) HCC with solitary ≤ 8 cm nodule, ineligible for or declined liver resection, not planned for transplantation and with clinically compensated cirrhosis (Child–Pugh score ≤ B7).

#### Eligibility criteria

Patients must meet all the inclusion criteria and none of the exclusion criteria below to be eligible for this trial.

Inclusion criteria

1. Histological or radiological diagnosis of single, new HCC with largest diameter ≤ 8 cm (BCLC stage 0 or A).
  - If prior history of HCC, the prior HCC must have been:
    Early stage, solitary HCC, ≤ 5 cm in size and,
    Have arisen within a different liver segment to the current HCC and,
    Treated with curative intent therapy > 2 years prior with no evidence of active disease at the site.
2. As per local multidisciplinary HCC meeting consensus patient is suitable for percutaneous thermal ablation and/or transarterial therapies and not suitable for or declined liver resection and not planned for liver transplantation.
3. Child–Pugh score ≤ B7 with no or diuretic-controlled ascites
4. ECOG performance status ≤ 2
5. Platelets ≥ 50 × 10^9^/L, Haemoglobin ≥ 80 g/L, Neutrophils ≥ 1.0 × 10.^9^/L, INR < 1.8 (except if on therapeutic anticoagulation)
6. 18 years of age or older and able to provide written consent

Exclusion criteria

1. Presence of multifocal HCC, macrovascular invasion or extrahepatic disease
2. Prior treatment for any HCC within last 2 years.
3. Clinically evident ascites or hepatic encephalopathy
4. Prior abdominal radiation therapy that would preclude the delivery of protocol defined SABR to the tumour
5. Untreated Hepatitis B or C 
6. Known additional invasive malignancy (excluding non-melanoma skin cancer) that is progressing or required treatment within the last 2 years.
7. Pregnancy 

#### Randomization and blinding

Randomization is conducted centrally via a secure web-based platform using blocks of size 4 to help ensure minimal imbalance in the size of treatment groups for each cohort. Participants in each cohort will be stratified for Child–Pugh classification (A vs. B7) and BCLC stage (0 vs. A). Participant and treating clinicians will not be blinded to the treatment assignment after the randomization process. Given the distinct post-treatment imaging appearances of the treated area following different therapies, it is also not feasible to reliably blind the local radiologists or central independent radiological review to treatment assignment.

### Treatment interventions

All primary therapy interventions are to commence within 28 days of randomization and must be completed within 42 days of randomization. This time window allows for additional percutaneous thermal ablation procedures (if initial ablation incomplete) or repeat TACE procedures or combination therapy (e.g., TACE-RFA for HCC > 3 cm) as part of the first line standard of care therapy.

#### Percutaneous thermal ablation

MWA or RFA will utilise CT or ultrasound guidance aiming for an ablation margin of > 5 mm. An adequate ablation margin must be confirmed on a post-ablation contrast-enhanced CT, preferably immediately post-ablation or within 24 h. In the event of initial incomplete ablation, further attempts at complete ablation are recommended. Documentation of procedures and adequacy of ablation margin are required to be submitted to TROG for all cases. In addition, central review of the post-ablation CT and verification of ablation margin will be conducted for each centre’s first treatment. Once each centre has passed the central quality assurance (QA) review, subsequent real time reviews will be sampled in a 1:5 ratio.

MWA or RFA may be used in cohort 2 for tumours > 3 cm if this represents standard of care therapy at the institution. It may be combined with transarterial therapies. All therapy is required to be completed within the primary therapy window of within 42 days from randomisation. However, MWA or RFA alone is not allowed for tumours ≤ 3 cm in cohort 2 as this is a specific exclusion criterion for cohort 2. These patients are eligible for cohort 1.

#### Transarterial therapy

Transarterial therapy may include TACE or TARE and can be administered as per local practice. TARE should aim to deliver a mean tumour dose of ≥ 120 Gy. Multiple TACE procedures are allowed to attain the best response; however, treatments should be completed within the primary therapy window of within 42 days of randomization.

#### Stereotactic Radiotherapy

SABR must commence within 28 days of randomisation and be completed within 42 days of randomisation. The local investigator has discretion over the prescription dose and fractionation schedule, within the ranges stipulated in Table 1. The aim is to deliver the highest possible biological dose to the planning target volume (PTV) from the protocol defined dose schedules, whilst adhering to the organ at risk (OAR constraints (Table 2). For participants with well compensated liver function (Child–Pugh A) and with tumours not abutting dose limiting organs, a schedule delivering ≥ 100 Gy BED10 is recommended. This corresponds to 42-45 Gy in 3 fractions or 50 Gy in 5 fractions delivered to ≥ 95% of the PTV. Treatment fractions are recommended to be delivered on 2–3 non-consecutive days per week, over a period of ≤ 15 days.

**Table 1:** Recommended SABR dosing schedules

Criteria	Dose	Fractions	Bed_10_
CP-A no dose limiting OAR^a^	50 or 42–45	5 3	100 100–112
CP-B7 no dose limiting OAR^a^	40–50 36	5 (preferred) 3	72–100 79
Dose limiting OAR^a^	35–40	5	60–72

*Abbreviations*: *BED* biologically effective dose, *CP* Child–Pugh, *OAR* organ at risk, *SABR* stereotactic ablative body radiotherapy

^*^Including meeting liver dose constraints

**Table 2:** Organ at risk (OAR) constraints

**Standardised name**	**Constraint**	**Per protocol**	**Minor variation**	**Major variation**
**a: OAR Dose Constraints for 5 Fraction Schedules**				
**Esophagus**	D0.1 cc	≤ 33Gy	> 33Gy but ≤ 35Gy	> 35 Gy
**Stomach**	D0.1 cc	≤ 32Gy	> 32Gy but ≤ 35Gy	> 35 Gy
	D10cc	≤ 25 Gy	> 25Gy	-
**Duodenum**	D0.1 cc	≤ 32Gy	> 32Gy but ≤ 35Gy	> 35 Gy
	D5cc	≤ 25 Gy	> 25Gy	-
**SmallBowel**	D0.1 cc	≤ 32Gy	> 32Gy but ≤ 35Gy	> 35 Gy
	D10cc	≤ 25 Gy	> 25Gy	-
**LargeBowel**	D0.1cc	≤ 34Gy	> 34Gy but ≤ 38Gy	> 38 Gy
**Heart**	D0.1cc	≤ 34Gy	> 34 but ≤ 38Gy	> 38Gy
	D30cc	≤ 30Gy	> 30Gy	-
**SpinalCord_PRV**	D0.035cc	≤ 25.3Gy	-	> 25.3 Gy
**Kidneys**	Dmean	≤ 10Gy	> 10Gy	-
	D ≥ 200cc*	≤ 15Gy	> 15Gy but ≤ 17.5Gy	> 17.5Gy
Kidney_R; Kidney_L*	V10Gy	≤ 10%	> 10% but ≤ 45%	> 45%
**BileDuct_Common**	D0.1cc	≤ 50Gy	> 50Gy	-
**Gallbladder**	D0.1cc	≤ 55Gy	> 55Gy	-
**Chestwall**	D0.5cc	≤ 50Gy	> 50Gy	-
**Skin**	D0.1cc	≤ 39.5Gy	> 39.5Gy	-
**b: OAR Dose Constraints for 3 Fraction Schedules**				
**Esophagus**	D0.1 cc	≤ 23Gy	> 23Gy but ≤ 25.2Gy	> 25.2 Gy
**Stomach**	D0.1 cc	≤ 20Gy	> 20Gy but ≤ 22.2 Gy	> 22.2 Gy
	D10cc	≤ 16.5Gy	-	> 16.5Gy
**Duodenum**	D0.1 cc	≤ 20Gy	> 20Gy but ≤ 22.2 Gy	> 22.2 Gy
	D10cc	≤ 11.4Gy	-	> 11.4Gy
**SmallBowel**	D0.1 cc	≤ 23Gy	> 23Gy but ≤ 25.2Gy	> 25.2 Gy
	D5cc	≤ 17.7Gy	-	> 17.7Gy
**LargeBowel**	D0.1cc	≤ 26Gy	> 26Gy but ≤ 28.2Gy	> 28.2 Gy
**Heart**	D0.1cc	≤ 26Gy	> 26Gy but ≤ 30Gy	> 30Gy
**SpinalCord_PRV**	D0.035cc	≤ 20.3Gy	-	> 20.3 Gy
**Kidneys**	Dmean	≤ 8.5Gy	> 8.5Gy	-
	D ≥ 200cc**	≤ 14Gy	> 14Gy but ≤ 16Gy	> 16Gy
Kidney_R; Kidney_L**	V10Gy	≤ 10%	> 10% but ≤ 33%	> 33%
**BileDuct_Common**	D0.1cc	≤ 36Gy	> 36Gy but ≤ 39 Gy	> 39Gy
**Gallbladder**	D0.1cc	≤ 42 Gy	> 42Gy	-
**Chestwall**	D0.1cc	≤ 36.9Gy	> 36.9Gy	-
	D30cc	≤ 30Gy	> 30Gy	-
**Skin**	D0.1cc	≤ 33Gy	> 33Gy	-

*This constraint is described as the minimum critical volume of the organ that must receive a specified threshold dose or lower (i.e. be spared from receiving a dose higher than the threshold dose) and may be termed a ‘cold’ constraint. For example, for a combined kidney volume of 250 cc, at least 200 cc should receive a dose of 14Gy (3 fraction schedule) or lower (D≥200cc ≤14Gy, used as a ‘cold’ constraint*; or D50cc ≤14 Gy, used as a ‘hot’ constraint). Some treatment planning systems allow the user to record ‘cold’ constraints directly. However, many do not and therefore these require adjusting into a ‘hot’ (standard) constraint format

**This constraint is applicable for participants with solitary kidney or where Kidney_L or Kidney_R Dmean >10Gy

Each centre will undergo pre-trial credentialing with specific focus on images used for treatment planning, image spatial registration and respiratory motion management to minimise geometric uncertainty in treatment delivery. Documentation of procedures and adherence to protocol defined treatment are required to be submitted to TROG for each case. In addition, each centre’s first case will undergo central QA review prior to commencing therapy including an assessment of the adequacy of target volume delineation, verification of motion management strategy and on-treatment imaging verification. After passing the first case, subsequent cases will be sampled in a 1:5 ratio.

### Assessments

Participant assessments are tabulated in Table 3. Radiological progression is defined as per mRECIST. Given the potential for more complex vascular changes following radiation-based therapies, local progression events will also include progression on RECIST v1.1 criteria. For cohort 1, local progression will include any recurrence within 10 mm of the ablation zone for MWA/RFA or within 15 mm of the target lesion for SABR [17]. For SABR treatments this will equate to a > 10 mm margin on the high dose region, i.e. the PTV which itself is a ≥ 5 mm margin on the target lesion. Central, independent radiological review will be undertaken for progression events with equivocal findings or non-consensus regarding progression requiring interval imaging to confirm progression (backdated to the previous imaging time point).

**Table 3:** Schedule of assessments

Assessment	PeR Randomisation	End oftreatment	Follow up										Progression^12^
			**Post treatment**	**Post randomisation**									
			**4 wks**	**3 mo**	**6 mo**	**9 mo**	**12 mo**	**15 mo**	**18 mo**	**21 mo**	**24 mo**	**30 + mo **^**1**^	
**Eligibility confirmation**	✔												
**Informed consent**	✔												
**Medical History**	✔												
**Clinical assessment**^**2,3**^	✔	✔	✔	✔	✔	✔	✔	✔	✔	✔	✔	✔	
**Concomitant Medications**^**4**^	✔	✔	✔	✔	✔	✔	✔	✔	✔	✔	✔	✔	
**Bloods**^**5**^	✔		✔	✔	✔	✔	✔	✔	✔	✔	✔	✔	
**Hepatitis status**^**6**^	✔												
**Abdominal Imaging **^**7,8**^	✔			✔	✔	✔	✔	✔	✔	✔	✔	✔	
**Chest Imaging**^**9**^	✔												✔
**Pregnancy Test**^**10**^	✔												
**Patient Reported Outcomes**^**11**^	✔		✔		✔		✔		✔		✔	✔	
**Adverse Event Reporting** **(CTCAE v5)**		✔	✔	✔	✔	✔	✔	✔	✔	✔	✔	✔	
**Participant experience interview**			^**✔13**^										

Key

^1^Subsequent follow up every 6 months thereafter until study completion

^2^To include: ECOG Performance status, presence of ascites or encephalopathy, weight, body mass index

^3^To confirm absence of uncontrolled ascites or encephalopathy only (ECOG, weight, BMI not required)

^4^After disease progression confirmed, only subsequent regimens of anti-cancer therapy will be recorded

^5^Includes FBC (Hb, platelets, WCC, neutrophils, lymphocytes), INR, PT/APTT, AST, ALT, ALP, GGT, total bilirubin, albumin, creatinine, sodium and AFP

^6^Documentation of hepatitis B & C virological status and need for treatment

^7^Multiphase contrast-enhanced CT or MRI of the liver. See Appendix 3 Imaging Guidelines for recommended CT and MRI sequences

^8^For thermal ablation treatments in cohort 1 only, a contrast enhanced CT is required to verify the ablation margin within 24 h of ablation. See Sect. 6.1.1 Quality Assurance (QA) measures for MWA/RFA

^9^CT chest (± pelvis)

^10^Women with childbearing potential only. Testing as per institutional protocol

^11^EORTC QLQ-C30, QLQ-HCC18

^12^Follow-up should continue beyond progression to allow for collection of subsequent therapies, QoL, and second sites of progression

^13^If consented, the interview will be conducted by a central designated interviewer within 4 weeks of treatment completion

New intrahepatic and extrahepatic lesions are defined as per mRECIST. For extrahepatic progression mRECIST criteria excludes ascites and pleural effusion unless cytologically confirmed but otherwise retains the RECIST v1.1 criteria for measurable disease.

#### Management of disease progression

After per-protocol progression has been confirmed by central radiological review, treatment will be according to local institutional standard of care. Crossover therapy is permitted. Relevant data (subsequent treatments, further progressions, survival status) will continue to be collected post progression events.

### Statistical analysis and sample size

For cohort 1 (HCC ≤ 3 cm and eligible for thermal ablation) the accrual target is 118 participants. A 20% difference in 2-year FFLP was considered clinically significant for this surrogate endpoint given the potential for local salvage and multiple subsequent lines of therapy. We estimated a 2-year FFLP of 75% in the thermal ablation arm. Assuming a 2-sided type 1 error rate of alpha = 0.05, 47 evaluable patients per arm will provide 80% power to detect an absolute difference in 2-year FFLP of 20% (95% SABR versus 75% thermal ablation). The required sample size will be inflated to *N* = 47/0.80 = 59 per arm to allow for a drop-out rate of 10% and a 2-year mortality rate of 10%.

For cohort 2 (HCC > 3 cm or ineligible for thermal ablation if ≤ 3 cm) the accrual target is 100 participants. We estimated a 2-year FFLP for the standard of care (SOC) therapies of 65%. Assuming a 2-sided type 1 error rate of alpha = 0.05, a sample size of 40 patients per arm will provide 80% power to detect an absolute difference in event rates at 2 years of 25% (90% SABR versus 65% SOC) [23–25]. Assuming a 10% drop-out rate and 10% mortality rate at 2 years the estimated number of patients per treatment arm is *N* = 40/0.80 = 50 per arm.

All analyses will be performed according to an intention-to-treat protocol, with a per-protocol analysis performed as a sensitivity analysis. For the FFLP, PFS, time to tumor progression, tumor objective response rate and OS endpoints, analysis will be performed comparing 2-year estimates from cumulative incidence (FFLP) and Kaplan–Meier survival curves (PFS and OS). The comparisons between groups (SABR vs. MWA/RFA and SABR vs. SOC) will be analysed separately. Secondary analysis using Gray and log-rank tests will be performed to evaluate differences in overall time to event distributions. Fine-Gray, Cox proportional hazards regression will be used to estimate hazard ratios with 95% confidence intervals. Secondary outcomes including the EORTC QLQ-C30 and QLQ-HCC18 (presented as mean scores over time) will be assessed using linear mixed-effects models to account for repeated measures. Additionally, time to deterioration of combined global health status/quality of life will be analysed. Time to deterioration will be defined as the time to first onset of a ≥ 10 point decrease from baseline. A 2-sided type-1 error rate of alpha = 0.05 will be used for all hypothesis testing. All analyses will be performed using Stata software (version 17).

Cost-effectiveness will be estimated as the incremental cost-effectiveness ratio for the use of SABR relative to the relevant comparator for each of the patient cohorts separately (percutaneous ablation eligible and stage migration patients, respectively and for an overall weighted analysis based on the proportion of early inoperable HCC patients in Australia anticipated to fall into these two groups). Outcomes for the cost-effectiveness analysis include the difference in the proportion of patients with FFLP at two years, and the difference in quality adjusted life years (QALYS) observed over the study period. In the first instance, outcomes will be expressed based on the primary outcome, the two-year FFLP rate, and the cost-effectiveness expressed as the cost per additional patient with local control at two years. QALYs, the combined impact of the impact of HCC and its treatment on survival and quality of life, will be assessed using patient completed information from the EORTC QLQ-C30. The base case analysis will adjust for the effect on OS of crossover post-progression. The potential for costs and outcomes to extend beyond the duration of the trial, and the resulting impact on the cost per QALY, will be explored in a modelled analysis.

### Research funding and governance

The trial is funded by an Australian Medical Research Future Fund (MRFF) grant, with no role in of study design, data collection, management, analysis, interpretation of data, writing of the report and publications. The trial is sponsored by TROG with oversight of study design, data collection, management, analysis, interpretation of data, writing of the report and publications. A trial management committee of key investigators and trial coordinators will meet 2-weekly to manage the study and monitor recruitment. A trial executive committee of all key investigators, site principal investigators and coordinators will meet 3-monthly during trial. The TROG Independent Data Safety Monitoring Committee, separate to the trial management committee, will independently monitor the conduct of the trial to ensure its ethical and scientific integrity.

### Trial status

The study is approved under the Australian national mutual acceptance ethical review framework by Peter MacCallum Cancer Centre Human Research Ethics Committee (HREC/86417/PMCC) and is registered in the anzctr.org.au trial database: ACTRN12621001444875. Accrual commenced in December 2022. It is estimated it will take 3 years to complete accrual with a further 2 years minimum follow-up for participants.

## Discussion

Current standard of care management of unresectable, early-stage HCC has limitations with variable local control rates and frequent treatment stage migration. Promising non-randomized evidence suggests a role for SABR as primary therapy in this setting, however, a lack of randomized evidence has hampered its incorporation into clinical guidelines with resultant considerable variation in practice. The SOCRATES-HCC study will provide a randomized, multicentre evaluation of the efficacy, safety, cost-effectiveness and patient acceptability of SABR versus other SOC therapies in this setting. Such randomized studies have yet to be successfully completed but have the potential to change current treatment algorithms. A significant strength of our study lies in its broad national, multisite collaboration led by hepatology units around the country in conjunction with radiation oncology and interventional oncology colleagues, coordinated by an experienced collaborative trials group (TROG) and support from other professional and trial groups (GESA, ARGANZ and AGITG).

### Supplementary Information

Supplementary Material 1.

Supplementary Material 2.

Supplementary Material 3.

## Data Availability

No datasets were generated or analysed during the current study.

## Abbreviations

AGITG: Australian Gastrointestinal Trials Group
ARGANZ: Abdominal Radiology Group of Australia and New Zealand
BED: Biologically effective dose
BCLC: Barcelona Clinic Liver Cancer staging
CT: Computerised Tomography
CTCAE: Common Terminology Criteria for Adverse Events
ECOG: Eastern Co-operative Oncology Group
FFLP: Freedom from local progression rate
GESA: Gastroenterological Society of Australia
Gy: Gray
HCC: Hepatocellular Carcinoma
HREC: Human Research Ethics Committee
MWA: Microwave ablation
OAR: Organ at Risk
OS: Overall survival
PFS: Progression free survival
PTV: Planning Tumour Volumes
QA: Quality Assurance
QALY: Quality-adjusted life year
RECIST: Response evaluation criteria in solid tumours
RFA: Radiofrequency ablation
SABR: Stereotactic ablative body radiotherapy
SOC: Standard of Care
TACE: Transarterial chemoembolisation
TARE: Transarterial radioembolisation
TROG: Trans-Tasman Radiation Oncology Group
