# Supplementary material for: A randomised controlled trial of Standard Of Care versus RadioAblaTion in Early Stage HepatoCellular Carcinoma (SOCRATES HCC)
Source: BMC Cancer. 2024 Jul 8;24:813. doi: 10.1186/s12885-024-12504-2 (PMC11229272; doi:10.1186/s12885-024-12504-2)
Supplement: Supplementary file 1 — Supplementary Material 1. [file 12885_2024_12504_MOESM1_ESM.pdf]

CLINICAL STUDY PARTICIPANT INFORMATION AND CONSENT FORM

PART A
GENERAL INFORMATION

A

NationalPICF.com.au

Clinical Studies

What is a clinical study?

"We do studies to find out the best way to provide medicine – we are trying to see if they are safe and that they work".

Clinical studies are research investigations in which people volunteer to help evaluate new procedures, or tests that prevent, detect, treat or manage various diseases or medical conditions.

This helps to determine if an intervention works and is safe, and if it is better than interventions that are already available.

Clinical studies can be used for different purposes.
Some clinical studies look at how people respond to a new intervention and what side effects might occur. Clinical studies might also compare existing interventions, test new ways to use or combine existing interventions, or observe how people respond to other factors that might affect their health.

Clinical study interventions could include:

- preventive care strategies
- vaccines, cells and biological products
- behavioural therapies
- surgical and medical treatments and procedures
- medical devices
- drugs
- health service changes
- education
- diagnostic tests

Who has approved this clinical study?

A Human Research Ethics Committee (HREC) approved this study.

The HREC checks that the study meets with the standards of the Australian National Statement on Ethical Conduct in Human Research (2007). The National Statement ensures that the interests, safety and wellbeing of people who take part in studies are protected.

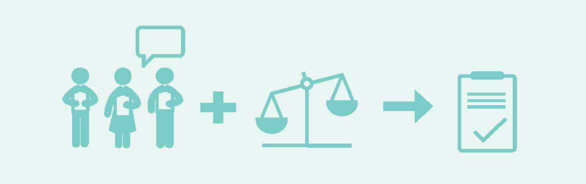

Clinical studies and you

Participant information and consent

Everyone taking part in a clinical study must give informed consent, or have a parent, guardian or other legally authorised person give informed consent on their behalf, before they enter the study.

To help you decide whether or not to be part of a study, members of the study team will explain the details of the study to you. They will also give you information to read (the participant information and consent form).

After talking with the researchers and reading the study details, you can decide whether to participate. You do not have to agree to participate in the study.

If you do agree to enter the study, you will be asked to sign a consent form.

After you sign the form, the researchers will give you a copy to refer to during or after the study.

The participant information and consent form has three parts:

A General information

This booklet introduces you to clinical studies and informed consent, and provides some questions that you might want to ask the clinical study team.

B Study details

An information sheet with details about a specific study, including its purpose, duration, required procedures, risks and any potential benefits.

C Consent form

A form that you, a parent/guardian or a person responsible, sign if you wish to participate in a trial, to show that you voluntarily agree to take part and understand what your involvement will be.

What is informed consent?

Informed consent means that potential participants are given information about the key facts of a clinical study before deciding whether or not to take part.

If you are asked to take part in a clinical study, you are free to say yes or no at any time. There is no pressure on you to enter or stay in a study.

You are encouraged to ask questions about anything that is not clear to you or that you do not understand. Take your time, and talk it over with family and friends, or your regular GP before deciding whether to take part.

If you decide to take part, you will be asked to sign the consent form. The consent form is not a contract, and you may leave the study at any time without losing any of your normal rights. If there are any changes to the study, you will be kept informed and you may be asked to give your consent again before continuing with the study.

Signing the consent form means that you:

- Understand what you have read
- Consent to take part in the study
- Consent to the activities described in the participant information and consent form
- Consent that your personal and health information can be accessed by the researchers
- Consent that your general practitioner and/or treating specialist will be notified of your participation in the clinical study as well as any clinically relevant information noted by the researchers during your participation in the study.

What happens to information about me?

If you sign the consent form, you are agreeing that the researchers may collect and use information about you for the study in the ways they have described in the document. This could include information that identifies you, and details about your health now, in the past or in the future. As well as information, the researchers may collect and use samples.

What happens if I leave a clinical study? Can a clinical study be stopped?

You can decide to stop taking part in a study at any time, for example if your condition is getting worse, you are finding it difficult to participate or you have concerns.

You can choose to leave a study at any time without giving a reason and without any effects on the care that you will continue to receive. If you do withdraw from a clinical study, the relationship between you and your doctor will not be affected. It is important to discuss your decision to leave a study with the research team before you leave, so that they can advise you about any safety or follow-up requirements and what will happen to information about you that has been collected for the study.

If there are signs that the intervention in a study could be unsafe, the study team or the regulators monitoring the study will stop the study. In addition, if the new intervention is found to be clearly superior or inferior during a study, the study may be stopped. Your care will always be followed up.

If you do withdraw your consent during the clinical study, the research team will stop collecting personal information about you.

Asking questions

Some questions you may want to ask after you have read Part B

If you are thinking about being part of a clinical study, you should know as much as possible about the study, your involvement in it and the commitment you are making.
You might find some of the answers to your questions in Part B of the participant information and consent form.

You can also discuss your questions with the study team and your doctor. Consider taking a family member or friend along to the discussion for support and help in asking questions or recording answers.

Plan what to ask ahead of time.
Don't hesitate to ask any new questions that you think of during the study.

Some questions that you may want to ask

- About the Study
  - What is the aim of the study? Will it help people? Will it help me?
  - Has the intervention been tested before? What was the outcome? Can it help others?
  - Will the study use a placebo (a substance with no clinical effect?)
  - Who is conducting and paying for this research?
  - What are the alternatives to participation?
  - Will any samples will be collected? How often? Are there any risks associated with the collection of the samples? What will happen to the samples after the study?
  - Will any study tests provide information about the current or future health of me or my family members?
- About your involvement
  - How might this study affect my daily life? How much of my time will be needed?
  - What kinds of tests and procedures are involved?
  - Who can I contact for support and information during the study? Will someone be available 24 hours a day?
- About costs
  - Who will pay for the experimental intervention?
  - Will my expenses be covered?
  - If complications arise from the study, who is responsible for paying any costs associated with them?
- About what happens after the study
  - What follow-up care, if any, is available after the study?
  - How long will it be before the results of the study are known?
  - How do I find out the results of the study?
  - Will I have access to the experimental intervention after the study if I wish to continue with it?

The Australian clinical trials website lists some other things you might want to discuss and suggests additional information sources: [www. australianclinicaltrials.gov.au/how-be-part-clinical-trial)](www.australianclinicaltrials.gov.au/how-be-part-clinical-trial)

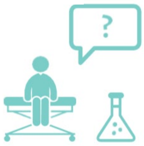

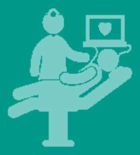

The Study Contacts

Part B of the participant information and consent form will contain contact details for people involved in the study. There are normally three key contacts:

- The study team
  (the researchers and support staff)
- The research site
- The Human Research Ethics Committee
  (This committee reviews the study for its scientific merit and protects the interests of trial participants, and may provide independent advice).

Please keep the participant information and consent form, and the contact details, in case you would like to talk to any of these people. You can contact them at any time during the study to:

- Ask questions about the study or your treatment
- Ask for advice about anything that concerns you
- Make suggestions about the study or your treatment
- Compliment the study staff or organisation
- Complain about any aspect of the study

Notes

For further information about clinical trials, visit the Australian clinical trials website: www.australianclinicaltrials.gov.au/consumers

Participant information and consent form – Consent form

[insert institution header]

Participant information and consent form – Study details

**Title**	**A randomised controlled trial of Standard Of Care versus RadioAblaTion in Early Stage HepatoCellular Carcinoma**
**Short Title**	**SOCRATES HCC**
**Project Number**	TROG 21.07
**Project Sponsor**	Trans Tasman Radiation Oncology Group (t/a TROG Cancer Research)
**Principal Investigator**	[Principal Investigator]
**Location**	[Location]

1. Would you like to take part in this clinical study?

We invite you to take part in our clinical study testing different treatment options for early stage hepatocellular carcinoma (liver cancer). This is because you have been diagnosed for early stage hepatocellular carcinoma with a single lesion within your liver and surgery is either not possible, or other health problems make surgery high risk or you have decided not to have surgery.

This document tells you about the study and describes what will happen if you take part. If there is anything you don't understand or want to know more about, please ask us.

If you don't know what to ask, there are some questions to consider in the *Clinical study participant information and consent form: Part A – General information*.

You might also want to talk to a relative, a friend or your GP before you make up your mind. You may also take this form away with you. If you decide to go ahead, we will ask you to sign the consent form (*the last page of this document*).

2. Why are we doing this research?

The SOCRATES HCC study aims to investigate whether a radiotherapy technique (called Stereotactic Ablative Body Radiotherapy or SABR) can improve outcomes for people with early stage liver cancer compared to other current treatments.

The current standard of care treatments can vary depending on your individual circumstances and include;

- Radiofrequency ablation (RFA) or microwave ablation (MWA) that use heating probes directly inserted into the tumour to kill the cancer cells.
- Transarterial chemoembolisation (TACE) or transarterial radioembolisation (TARE) that use chemotherapy or radioactive particles injected into the blood supply of the tumour to kill the cancer cells.

Stereotactic Ablative Body Radiotherapy (SABR) is a highly precise radiation therapy technique that uses high energy x-rays focused on the tumour to kill the cancer cells. It is currently used as an effective treatment for a number of other cancer types including early stage lung and prostate cancer. The main aim of this study is to see whether treatment with SABR can improve outcomes for people with early stage liver cancer compared to these other current treatments.

3. Do I have to take part in this research project?

Participation in any research is voluntary. If you do not wish to take part, you do not have to. If you decide to take part and later change your mind, you are free to withdraw from the project at any stage.

If you decide to take part, you will be given this participant information and consent form to sign and you will be given a copy to keep.

Your decision whether to take part or not to take part, or to take part then withdraw will not affect your routine treatment, your relationship with those treating you or your relationship with [Institution].

4. What is involved in the study?

This study aims to recruit 218 participants from across Australia. It is expected that these participants will be recruited over 2 years. Each patient will remain part of the study until the last patient recruited has reached 2 years after having treatment (this is called the end of the study). The study is expected to be active for about 5 years.

The main steps of the study are:

I. Agreeing to participate in the study (consenting)
II. Screening and Randomisation
III. Undergoing treatment
IV. Follow-Up

Most of these steps will be routine but others are needed to check that it is safe for you to participate/continue participating in the study. You are welcome to bring a family member or friend to all appointments.

I. Agreeing to participate in the study (consenting)

Your study doctor will talk to you about the study and if you agree to take part, they will ask you to sign the study consent forms. You will be given a copy of these forms to keep. Any trial specific procedures will only take place after you have signed the consent form.

II. Screening and Randomisation

After you have given your consent, your study doctor will arrange for a number of procedures to be conducted prior to confirming your inclusion in the study (screening). The clinic visit will take 20-30 minutes.

Procedure	Details
**Blood test**	A small amount of blood will be collected and sent to pathology to check the function of your organs, your blood chemistry, your blood cell count and your clotting factors.
**Clinical Assessment**	Your doctor will perform a check of your overall health and will include a measurement of your height and weight and will also ask you about any medication you are taking and any symptoms you are experiencing.
**Medical History**	You will be asked about your medical and surgical history
**Imaging**	To assess the location and size of your cancer, you will be required to have medical imaging of your abdomen and chest.
**Health and Quality of Life Questionnaire**	You will be asked to complete a questionnaire on symptoms from your cancer and their impact on your day to day life. It will take approximately 10-15 minutes to complete.

**Pregnancy test**	If you are a woman that has childbearing potential, you will be asked to take a pregnancy test.
**Demographic data**	The study team will record your age, postcode, gender, indigenous status and your primary language.

Once it has been confirmed you are suitable to join the study, you will be randomly assigned to one of the two possible treatment groups:

Group 1<br>Stereotactic Ablative Body Radiotherapy (SABR)	OR	Group 2<br>Standard of Care treatment (SOC)

This is a randomised controlled study. Sometimes we do not know which treatment is best for treating a condition. To find out we need to compare different treatments. We put people into groups and give each group a different treatment. The results are compared to see if one is better. To try to make sure the groups are the same, each participant is put into a group by chance (random). There is an equal chance of you being in Group 1 or Group 2.

If you have any questions about randomisation and what group you may be put into, you can ask your study doctor to talk about this further with you.

III. Undergoing Treatment

Before you start your allocated treatment, your study doctor will review your current symptoms and ask you to complete a Health and Quality of Life Questionnaire.

a. Group 1: Stereotactic Ablative Body Radiotherapy (SABR)

Patients in Group 1 will receive radiation therapy using a technique called Stereotactic Ablative Body Radiotherapy (SABR) that will be delivered in 3 or 5 outpatient treatment sessions (each 20 to 45 minutes in duration) spaced out over 1 to 2 weeks. The exact number of treatment sessions received, and the duration of each session depends on the size and location of your liver cancer.

In preparation for SABR treatment you will undergo an initial planning appointment where the treating team will lie you in a comfortable position on the treatment couch, take some measurements and perform a CT scan. This process generally takes one to two hours. An MRI scan may also be performed. In some cases a needle guided insertion of special markers into the liver near the tumour is performed to help guide the SABR treatment. A radiation oncologist will explain your individualised treatment to you.

b. Group 2: Standard of care therapies

Patients in Group 2 will receive standard of care treatment (SOC) as per your institutions local practice that will be administered by a doctor called an Interventional Radiologist.

The therapies you will be offered will depend on the size and location of your liver cancer and may include one or a combination of radiofrequency ablation (RFA) / microwave ablation (MWA) and/or transarterial chemoembolization (TACE) / transarterial radioembolisation (TARE). This may require an anaesthetic and an overnight admission to hospital.

Your Interventional Radiologist will explain your individualised treatment to you.

At the end of your therapy your study doctor will arrange a brief clinical assessment, including asking you about any symptoms and record any new medications. This will take 10-15 minutes.

IV. Follow-Up

Your study doctor will ask you to attend clinic 4 weeks after your therapy has completed. At this visit you will be asked how you are feeling and any possible side effects will be recorded. Some of the

same procedures that were conducted in screening will be repeated, to see if any of the results have changed. These include;

- a blood test to check the function of your organs, your blood chemistry, your blood cell count and your clotting factors
- a clinical assessment (10-15 minutes), including vitals, being asked about any changes to the medications you are taking and/or symptoms you are experiencing.
- a questionnaire on your health and quality of life (10-15 minutes)

  If you become upset or distressed as a result of your participation in this study, the researcher is able to arrange for counselling or other appropriate support. Any counselling or support will be provided by staff who are not members of the research team

Also at this visit, you will be invited to take part in a one-off telephone interview that will take approximately 20 minutes and will involve briefly talking about your treatment experience in the SOCRATES trial. The details about this interview are outlined in section 6.

After the 4 weeks after treatment visit, other regular follow-up visits will be scheduled so that your study doctor can check on your progress. These visits will be scheduled in 3 monthly intervals for 2 years and then 6 monthly until completion of the trial. The same procedures will be repeated but also include imaging; CT or MRI of your liver (and if applicable CT of your Chest). The questionnaire on your health status will only be collected 6 monthly until completion of the trial. .

In the event that you are unable to attend a clinic appointment, the study team may still contact you by phone and/or collect information about your health via your medical records or from other health services (like your GP or other local health clinics) and/or post you a copy of the questionnaire on your health status to fill out and return.

Figure 1: THE FLOW DIAGRAM shows the main time points for the study

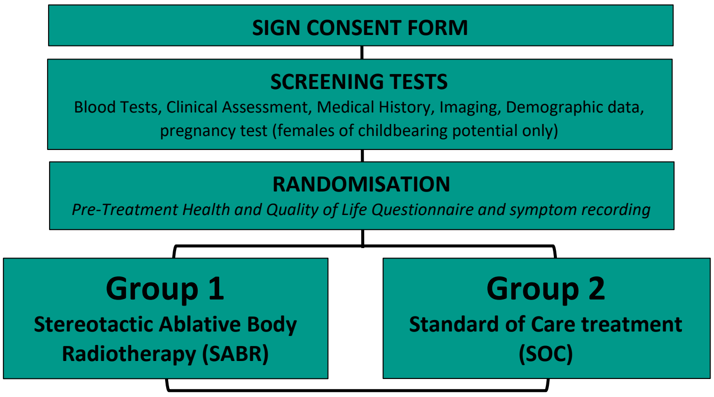

FOLLOW-UP

Visit	Clinical Assessment	Blood test	Imaging	Telephone interview on treatment experience	Health and Quality of Life Questionnaire
4 weeks after therapy	✓			✓	✓
3 months^	✓	✓	✓		
6 months^	✓	✓	✓		✓
9 months^	✓	✓	✓		
12 months^	✓	✓	✓		✓
15 months^	✓	✓	✓		

18 months^	✓	✓	✓		✓
21 months^	✓	✓	✓		
24 months^	✓	✓	✓		✓
30 months^	✓	✓	✓		✓
36 months^	✓	✓	✓		✓
6 monthly thereafter until trial completion	✓	✓	✓		✓

^after randomisation

5. Services Australia [Medicare Benefits Schedule (MBS) and Pharmaceutical Benefits Scheme (PBS)] data

One of the aims of this study is to look at the resource use and cost effectiveness of the different therapies. To do this, at the end of the study, the study doctors would like to assess your data held at Services Australia regarding what medical and pharmaceutical benefits you have accessed 6 months prior to and during your time on the study. You will be asked to sign a separate consent form authorising the study to access your Services Australia information, see the separate Services Australia Participant Information Document and Participant Consent Form, your study doctor will provide you with this consent form. Services Australia is not involved in this research other than to provide the information that you have consented to the release of, should you decide to participate in this study. Services Australia has confirmed that this research and any associated documents have received approval from a Human Research Ethics Committee (HREC) that is registered with and operates within guidelines set out by the National Health and Medical Research Council (NHMRC).

6. Telephone Interview on Treatment Experience

Once you have completed the study treatment, the researchers will invite you to talk about your treatment experience in a telephone interview. They are doing this to further understand the patient experience and see if this varies according to the type of treatment received (e.g., SABR, RFA / MWA and/or TACE / TARE). They are also interested in understanding each patient's level of satisfaction with their treatment experience.

Each interview will be conducted by an independent researcher, from Flinders Medical Centre, who is not part of the treatment or chief investigator research team. The interview will take approximately 20 minutes and will involve briefly talking about your treatment experience (i.e., you will be asked about the good and bad points of the treatment, if you think there is anything that would make the experiences better etc).

Only the staff at this centre and the interview will know your name and contact details, these won't be shared with anyone else in the study team and will only be used to conduct the interview. The interview will be recorded and the person interviewing you will check you are still happy to participate during the call before asking any questions. Your information will be kept secure, password protected and identified by a unique study number.

Taking part in this interview is your choice. If you say yes, the study team will ask for your permission for your contact details to be collected so that they can organise for the interview to take place around 4 weeks after your treatment has finished. If you say no, or change your mind after saying yes, it will not affect the care you receive for your cancer.

7. Who is conducting and paying for this research?

This research study is being sponsored by the Trans Tasman Radiation Oncology Group (aka TROG Cancer Research), a not-for-profit research group involving many cancer researchers in Australia, as well as internationally. This study has been awarded a grant by NHMRC Medical Research Future Fund. This is a federal government grant.

In addition, if knowledge acquired through this research leads to discoveries that are of commercial value to TROG Cancer Research, the study doctors or their institutions, there will be no financial benefit to you or your families from these discoveries.

No member of the research team will receive a personal financial benefit from your involvement in this study (other than their ordinary wages).

Some of the tests, medication and or treatments used in this study may be part of the standard care used to maintain your health even if you did not take part in the study. You will be responsible for the cost of this standard care in the usual way (health insurance, Medicare and your personal contribution depending on your circumstances). If you are Medicare ineligible, you may incur significant costs. You should discuss your individual circumstances with your study doctor prior to your participation in this research project.

Neither you nor your doctor will be paid for taking part in this study. [Institution name] will receive a nominal payment to cover hospital costs for the treatment and to offset the costs of conducting this research.

8. What if something new comes up during the study?

If we find something new about an intervention while the study is under way, the study doctor will discuss with you what it means and whether you want to continue in the study. If you decide to continue in the clinical study, we will ask you to sign an updated consent form.

9. Could the researchers stop the trial early?

Yes, it happens sometimes. If it does, the study doctor will let you know and explain the reason behind the decision. The study doctor will discuss and arrange continued treatment as needed for you.

10. What will happen to the confidential information about me?

We will keep all personal information confidential and securely stored.

Personal information about you, such as your name and address will be permanently removed from documentation before it leaves the clinic. Your personal information will be replaced with a unique study number and study centre.

11. What information will be collected, and how will it be stored?

Any information collected about you for this clinical trial will be kept confidential and securely stored. We will not disclose your information without your permission, except in compliance with the law.

Information concerning your participation in the study will be sent to the TROG Cancer Research central office in Newcastle (NSW), which coordinates the SOCRATES HCC study, by the study doctor or their designate.

- This may include copies of your medical records, medical reports, your radiotherapy treatment details and images from any scans to assess your progress (such as CT,MRI scans).

- This information will only be identified by a unique study number. In no instance will the study centre identify you by name on these documents. They have policies of strict confidentiality and will not release any information concerning you, except to other researchers in this study.
- A copy of your signed consent form for audit purposes. The form will be identified by a unique study number only.
- This information will also be sent to the TROG Cancer Research central office and will be stored indefinitely to be utilised in future research to advance our knowledge about cancer and its treatments. This is called Data Sharing and is not optional. Please see section 12 for further details.

All other information and data collected by [Name of institution] will be stored and destroyed in line with the hospital policy.

It is anticipated that the results of this study will be published and/or presented in a variety of forums. In any publication and/or presentation, the information will be provided in such a way that you cannot be identified.

Your health records and any information obtained during the research project may be subject to inspection (for the purpose of verifying the procedures and the data) by the relevant authorities and authorised representatives of the Sponsor, TROG Cancer Research, the institution relevant to this Participant Information Sheet, [Name of institution], or as required by law. By signing the Consent Form, you authorise the release of, or access to, this confidential information to the relevant study personnel and regulatory authorities as noted above.

Australian and [Insert the name of a state or territory] privacy law gives you the right to request access to your information that the researchers have collected and stored. The law also gives you the right to request corrections to any information about you that you disagree with. Please contact the study team (contacts in section 17 *Who do I contact if I have a question or complaint or seek compensation for injury?* of this document) if you would like to access your information.

By signing the consent form,

- You are giving us permission for the following to access, collect and send information concerning your participation in the study to the TROG Cancer Research central office, including a copy of your signed consent form.
- You are also giving permission for TROG Cancer Research to store indefinitely the information concerning your participation in the SOCRATES HCC study for use in this study and/or in additional or future research which has been approved by the appropriate Human Research Ethics Committee (HREC), conducted by researchers in the SOCRATES HCC study and their collaborators, who may work at other national or international locations or facilities.
    - You may opt out of the indefinitely storage of this data for future by completing the Opt-Out consent from at the end of this information sheet. This can be completed at any time thought out the duration of the study.

12. What tissue and blood samples will be collected and how will they be stored?

Routine blood tests will be collected to examine your full blood count and biochemistry. These tests will be used to determine your general health status and to screen for a variety of disorders, such as anaemia and infection, as well as nutritional status. The collection of these samples is a part of your routine care for your cancer so are not optional. All blood samples taken for this purpose will only be used for this clinical trial and will be stored and destroyed in line with the Hospital/Pathology laboratory's policy.

13. Will any of my information or samples be used in future research

Yes, unless you opt-out, your information (data) (excluding Services Australia information) may be used in future research. Data collected during the study will be analysed to answer this study's research question, but will also be stored indefinitely by the study sponsor to be utilised in future research to advance our knowledge about cancer and its treatments (Data Sharing);

- Data may include the information recorded about you for the study, including but not limited to your radiotherapy treatment details, images from any collected scans or cancer outcomes.
- This further research may be conducted by the study investigators, study sponsor and/ or collaborating researchers. Any third party will receive only coded or anonymised information.
- The future research may involve combining the data collected from multiple related trials in Australia and from around the world or looking at the data to answer additional research questions.
- Any future research will be approved by TROG Cancer Research and the SOCRATES HCC Trial Chair/s and/or other appropriate organisations, such as a Human Research Ethics Committee (HREC), before any data is shared.

14. What are my responsibilities during the study?

If you agree to participate in this study, you agree to be responsible for attending all study-specific appointments. You also agree to comply with the other conditions in this document. If you cannot, or do not wish to accept this responsibility, then we cannot accept you as a participant in the study. Either way you choose, you will still receive the best possible care whether or not you take part in this study.

15. Can I have other medicines or procedures during this clinical study?

The treatments on study are directed at controlling your cancer. You are unable to have any further cancer directed treatments without discussing it with your study doctor first.

The treatments given as a part of this study can react with other medications, so it is very important that you discuss your current medications with your study doctor and inform them if you are started on any new medications by another doctor, in case you need additional monitoring or the medication needs to be stopped for the time you are involved.

You must tell us about any procedures or medicines you may be taking. This is in your interest as well as important for the trial. You must tell us about any over-the-counter medications, vitamins or herbal remedies you are taking and about acupuncture or other alternative procedures because they may also interact or interfere with the study treatments. You must also tell us about any changes to these while you are participating in the clinical trial.

16. What possible benefits might I get by taking part?

We cannot guarantee that you will receive any benefits from this research. The study is being carried out to see if we can improve the way we treat your type of liver cancer. The quality of the treatments used in the study are closely monitored by the study team. It is hoped that stereotactic radiation therapy will be well tolerated and improve outcomes by reducing the chances of the cancer returning, however, there may be no clear benefit to you from your participation in this research.

17. What risks do I run by taking part?

Medical procedures, medicine and tests often have side effects. You may have no side effects, some or all of the side effects listed below. These side effects may be mild, moderate or severe. If you have any of these side effects, or are worried about them, talk with your study doctor.

Your study doctor will also be looking out for side effects, but there may also be side effects that the researchers do not expect or do not know about and that may be serious. Tell your study doctor immediately about any new or unusual symptoms that you get.

Many side effects go away shortly after the treatment ends. However, sometimes side effects can be serious, long lasting or permanent. If a severe side effect or reaction occurs, your study doctor may need to stop your treatment. Your study doctor will discuss the best way of managing any side effects with you. Some unwanted effects may actually not be related to the study, nevertheless it is important to document these.

Radiation therapy

There are many factors that can affect the type and severity of side effects, including the location and size of your tumour, and your general health. The table below outlines many of the possible known side effects, however, your radiation oncologist will discuss with you which specific side effects may be associated with your planned radiation treatment. It is important to note that in previous trials of SABR for liver cancer that SABR has been very well tolerated with significant side effects described in less than 5% of patients.

This list is not exhaustive so you may experience some effects not listed. You should contact your study doctor if you experience any side effects even if you are not sure that any problems you may have are

related to your cancer treatment.

Short term side effects (first few weeks)	Potential long term side effects (months to years)
**Common (20-50%)**<br>• Tiredness<br>• Temporary, minor changes in blood tests without symptoms<br><br>**Less common (<20%)**<br>• Reduced appetite<br>• Nausea (an anti-nausea medication is generally given prior to treatment to reduce this risk)<br>• Discomfort in the treated area<br>• Bowel changes - Loose bowels (or constipation from the nausea medication)<br>• Skin redness or dryness in the treated area<br><br>**Uncommon (<5%)**<br>• Significant liver damage (symptomatic)<br>• Stomach or bowel ulceration or bleeding (if tumour close to stomach or bowel)<br>• Discomfort or difficulty swallowing (if tumour close to oesophagus)<br>• If undergoing fiducial marker insertion – small risk of pain, bleeding or infection from procedure.	**Less common (<20%)**<br>• Reduction in liver function without symptoms<br>• Skin Changes<br>• If tumour close to chest wall - discomfort or rib fracture<br>• If tumour close to diaphragm – temporary discomfort, dry cough or shortness of breath<br><br>**Uncommon (<5%)**<br>• Symptomatic liver impairment<br>• Reduced kidney function (rarely requiring additional intervention).<br>• Permanently lower blood counts (eg platelets)<br><br>**Rare (less than 1 in 1000)**<br>• Secondary Cancer

Standard of Care Therapies

Risks associated with the standard of care therapies (microwave or radiofrequency ablation, transarterial chemoembolization, transarterial radioemblization) are well known and remain the same whether or not you are participating in this trial. One of the important aims of the trial will be comparing the differences in side effects between SABR and standard of care treatments. The risks of standard of care treatments will be explained by your Doctor before any of these treatments are scheduled. You may require some form of sedation (to make you relaxed and sleepy) or a general anaesthetic during the procedure. Pain or discomfort following the procedure may require medication for relief. Flu-like symptoms with fever, pain and nausea can occur in the days following thermal ablation or transarterial embolization which settles within a few days. Major infections, bleeding or damage to nearby organs are uncommon (<5%).

Radiation from diagnostic imaging scans

This research project involves exposure to a very small amount of radiation (from diagnostic imaging scans like CTs). As part of everyday living, everyone is exposed to naturally occurring background radiation and receives a dose of about 2 millisieverts (mSv) each year. *[Add wording as specified from Radiation Risk Assessment*] or [The radiological imaging associated with this study is the same as you would normally receive for your care at this hospital.]

Commented [RM1]: Site to select wording depending on site requirements (i.e. risk assessed by Radiation Safety Officer)

Reproductive risks

The effects of liver cancer radiotherapy on the unborn child and on the newborn baby are not known. All participants are advised to use effective contraception during the course of the research. *OR* All participants must avoid pregnancy during the course of the research.

Commented [RM2]: Site to select wording depending on site requirements (i.e catholic institution)

Female participants: It is advisable that you are not pregnant or breast-feeding and do not become pregnant during the course of treatment and for 2 months afterwards.

- If you are female and child-bearing is a possibility, you will be required to undergo a pregnancy test prior to commencing the research project
- If you do become pregnant whilst participating in the research project, you should advise your study doctor immediately. They will advise on further medical attention should this be necessary.

Male participants: Radiation may cause temporary abnormalities in sperm. You should refrain from fathering a child or donating sperm during the course of treatment and for 2 months afterwards.

- You should advise your study doctor if you father a child while participating in the research project. Your study doctor will advise on medical attention for your partner should this be necessary.

18. What happens if I am injured as a result of my participation in this trial?

If you suffer any injuries or complications that may be as a result of your participation in this study, you should immediately contact your doctor, who will assist you in arranging appropriate medical treatment.

- If you are eligible for Medicare, you can receive any medical treatment required to treat the injury or complication, free of charge, as a public patient in any Australian public hospital.
- If you are Medicare ineligible, you may incur significant costs. You should discuss your individual circumstances with your study doctor prior to your participation in this research project.

In the unlikely event of an injury caused by your participation in this study, compensation may be payable to you. TROG Cancer Research maintains a clinical trial's insurance policy. Please ask your doctor if you would like more information on this policy.

19. What happens when the study ends?

After the study ends, your doctors will still ask you to attend follow up visits as part of your ongoing care.

It may be a number of years before the results of this research are available. When the results are available they may be published as 'articles' in medical journals. A summary of the results will also be available to the public on the TROG Cancer Research website (www.trog.com.au) and will be published in the TROG community newsletter. Your identity will not be revealed in these publications. Please ask your study doctor if you want to know more about this.

In the unexpected case that this research is not published, then all patients will receive a written report.

20. What if I withdraw from this research project?

You are under no obligation to continue with the research study. You may change your mind at any time about participating in the research. People withdraw from studies for various reasons and you do not need to provide a reason. If you decide to withdraw from the project, please notify your study doctor or complete the 'Participant Withdrawal of Consent Form' at the end of this form. If you do withdraw your consent during the research project, the study doctor and relevant study staff will not collect study-specific information from you, although personal information already collected will be retained to ensure that the results of the research project can be measured properly and to comply with law. The study doctor would still like to collect information about how you are getting on, which is recorded during your routine care. A member of the research team will ask if you give your permission for this at the time of the withdrawal.

You should be aware that data collected by the sponsor up to the time you withdraw will form part of the research project results. If you do not want them to do this, you must tell them before you join the research group. If you withdraw from the study, your information that has already been analysed and/or included in a publication may not be able to be withdrawn or destroyed.

21. Who do I contact if I have a question or complaint or seek compensation for injury?

We have included several contacts for you below. Who you contact depends on what information you need.

For all study enquiries or if you want to talk to the study team at any time:

Principal Study Doctor

Name	[Name]
Position	[Position]
Telephone	[Phone number]
Email	[Email address]

Study Coordinator/Nurse

Name	[Name]
Position	[Position]
Telephone	[Phone number]
Email	[Email address]

If you experience any side effects or complications as a result of this clinical trial, you should contact the study team as soon as possible. They will arrange appropriate medical help:

24-hour medical emergency contact for the study team

Name	[Name]
Position	[Position]
Telephone	[Phone number]

If you wish to discuss the study with someone not directly involved, particularly in relation to matters concerning policies, information or complaints about the conduct of the study or your rights as a participant, you may contact:

Reviewing HREC name	Peter MacCallum Cancer Centre Ethics Committee
HREC Executive Officer	Ethics Coordinator
Telephone	(03) 8559 7450
Email	ethics@petermac.org
HREC Reference Number	

If you are injured or experience severe side effects, you can take your complaints or requests for compensation to;

Position	Research Governance Officer
Telephone	[Phone Number]
Email	[Email address]

22. The consent form

Sign the consent form only after you have made up your mind to take part in this clinical trial. If you wish, we will arrange for someone to read the form to you in a language you understand. All study participants must be provided with a signed and dated copy of the participant information and consent form for their personal record.

Consent form

**Title**	**A randomised controlled trial of Standard Of Care versus RadioAblaTion in Early Stage HepatoCellular Carcinoma**	
**Short title**	**SOCRATES HCC**	
**Protocol number**	TROG 21.07	
**Project sponsor**	TROG Cancer Research	
**Study doctor**	[Study doctor]	
**Clinical contact person**	[Name, location]	[Business number]
**24-hour medical contact**	[24-hour phone number]	[Email address]

Consent Agreement

I have read the Participant Information Sheet or someone has read it to me in a language that I understand.

I understand the purposes, procedures and risks of the research described in the project.

I have had an opportunity to ask questions and I am satisfied with the answers I have received.

I freely agree to participate in this research project as described and understand that I am free to withdraw at any time during the study without affecting my future health care.

I give permission for my doctors, other health professionals, hospitals or laboratories outside this hospital to release information to [name of institution] concerning my disease, medical history and treatment for the purposes of this project. I understand that such information will remain confidential.

I understand that the sponsors of this study may make my data available to other researchers for future research. I grant advanced permission for the possible future sharing of information collected about me with other organisations, with the understanding that I will not be identifiable from this information.

I consent to my treating doctor/s (including my GP) being notified of my participation in this study and any clinically relevant information noted by the study doctor in the conduct of the study.

I understand that I will be given a signed copy of this document to keep.

	**Yes**	**No**
	(please tick)	
I agree to participate in a telephone interview after completing my treatment to discuss talk about my treatment experience unless I have withdrawn from the study.	☐	☐

Declaration by Participant

Name of Participant (please print) ____

Signature ____________________________ Date ____________________________

Under certain circumstances, a witness* to the informed consent is required *(see Note for Guidance on Good Clinical Practice CPMP/ICH/135/95 at 4.8.9)*

Name of Witness (please print) ____

Signature ____________________________ Date ___________________________

** Witness is not to be the Investigator, a member of the study team or their delegate. Witness must be 18 years or older.*

# If a subject is unable to read or if a legally acceptable representative is unable to read, an impartial witness should be present during the entire informed consent discussion. By signing the consent form, the witness attests that the information in the consent form and any other written information was accurately explained to, and apparently understood by, the subject or the subject's legally acceptable representative, and that informed consent was freely given by the subject or the subject's legally acceptable representative.

Declaration by Study Doctor/Senior Researcher†

I have given an explanation of the clinical study, its procedures and risks and I believe that the participant has understood that explanation.

Name of Study Doctor/ Senior Researcher† (please print) ________________________________

Signature ____________________________ Date ________________________________

† A senior member of the research team must provide the explanation of, and information concerning, the research project.

Declaration by Interpreter (if applicable)

I am a qualified interpreter. I have given an explanation of the research project, its procedures and risks and I believe that the patient has understood that explanation.

Name of Interpreter (please print) ____

Signature ____________________________ Date ________________________________

Note: All parties signing the consent section must date their own signature

Opt-out of indefinite data storage

**Title**	**A randomised controlled trial of Standard Of Care versus RadioAblaTion in Early Stage HepatoCellular Carcinoma**
**Short Title**	**SOCRATES HCC**
**Project number**	TROG 21.07
**Project Sponsor**	TROG Cancer Research
**Principal Investigator**	[Principal Investigator]

I wish to withdraw my consent for TROG Cancer Research indefinitely store my data after the study has been completed.

Name of Participant (please print) ____

Signature ___________________________ Date ___________________________

Participant consent form – Withdrawal of Participation

**Title**	**A randomised controlled trial of Standard Of Care versus RadioAblaTion in Early Stage HepatoCellular Carcinoma**
**Short Title**	**SOCRATES HCC**
**Project number**	TROG 21.07
**Project Sponsor**	TROG Cancer Research
**Principal Investigator**	[Principal Investigator]

Declaration by Participant

I wish to withdraw my participation in the above research project effective from the date below. I understand that:

1. choosing to withdraw from the study will not affect my routine treatment, my relationship with those treating me or my relationship with [Institution], or my access to Health Services or Government benefits
2. that the trial data already collected by the sponsor will form part of the research project results, as described in the Participant Information Sheet any information about me that has already been analysed and/or included in a publication by the study, may not be able to be destroyed

I request that the study handles the following information they have collected about me in the following way:

INFORMATION HELD	DESTROY* (please tick)	RETAIN^
Scan images that have been collected for the study *(i.e. CT scans)*	☐	☐

* Information is deleted so it can no longer be used for research
^ Information or samples collected can continue to be used for research

ROUTINE CARE INFORMATION	YES (please tick)	NO
I consent that any further information collected in my routine care be used in the research study	☐	☐

Name of Participant (please print) ____

Signature _______________________________ Date _______________________________

If the participant's decision to withdraw is communicated verbally, the Study Doctor/ Senior Researcher will need to describe the circumstances below.

Declaration by Study Doctor/Senior Researcher[†]

I have given a verbal explanation of the implications of withdrawal from the trial and I believe that the participant has understood that explanation.

Signature ____		Date____________
Name of Study Doctor/ Senior Researcher[†] (please print)	____________________________________	

[†] A senior member of the research team must provide the explanation of, and information concerning, this research study.

Note: All parties signing the consent section must date their own signature
