# Supplementary material for: A randomised controlled trial of Standard Of Care versus RadioAblaTion in Early Stage HepatoCellular Carcinoma (SOCRATES HCC)
Source: BMC Cancer. 2024 Jul 8;24:813. doi: 10.1186/s12885-024-12504-2 (PMC11229272; doi:10.1186/s12885-024-12504-2)
Supplement: Supplementary file 2 — Supplementary Material 2. [file 12885_2024_12504_MOESM2_ESM.docx]

| 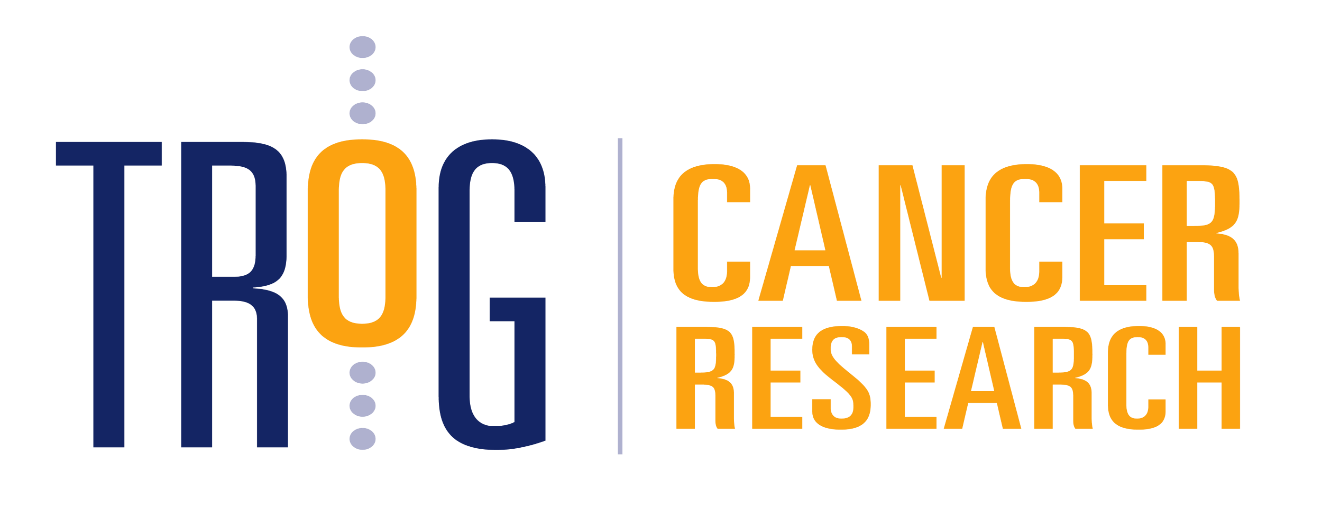 | | |
| --- | --- | --- |
| 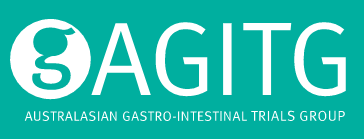 | 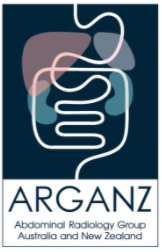 |  |

**TROG 21.07**

**SOCRATES HCC**

**A randomised controlled trial of Standard Of Care versus RadioAblaTion in Early Stage HepatoCellular Carcinoma**

| **PROTOCOL NUMBER:** | **21.07** |
| --- | --- |
| **SPONSOR:** | Trans Tasman Radiation Oncology Group  *(t/a TROG Cancer Research)* |
| **ANZCTR:** | ACTRN12621001444875 |
| **PROTOCOL VERSION:** | **3.0 25 August 2023** |

**Protocol Version History**

| **Date** | **Type** | **Number** |
| --- | --- | --- |
| 09 May 2022 | Original | 1.0 |
| 20 June 2022 | Amendment | 1.1 |
| 22 July 2022 | Amendment | 1.2 |
| 01 June 2023 | Amendment | 2.0 |
| 25 August 2023 | Amendment | 3.0 |

**FOREWORD**

This document is intended to describe a Trans Tasman Radiation Oncology Group (TROG) study and to provide information about procedures for screening, enrolling and treating study participants. It is not intended that the Protocol be used as a guide for the treatment of patients who are not enrolled on this trial.

TROG will not accept any data for analysis unless each participating site has all the applicable approvals in place (such as ethical and local governance) for patient enrolment and participation in this study.

Amendments to the document may be necessary; when approved by TROG, these will be circulated by the Trial Coordinating Centre, on behalf of TROG, to Trial Sites participating in the Study.

# TRIAL PERSONNEL

| **Trial Chair**  *(Hepatology Lead)* | **Prof Alan Wigg**  Flinders Medical Centre  Flinders Drive, Bedford Park, SA 5042  E: [**Alan.wigg@sa.gov.au**](mailto:Alan.wigg@sa.gov.au) |
| --- | --- |
| **Trial Chair**  *(Radiation Oncology lead)* | **A/Prof David Pryor**  Metro South Hospital and Health Service  199 Ipswich Road Woolloongabba, QLD 4102  E: [**david.pryor@health.qld.gov.au**](mailto:david.pryor@health.qld.gov.au) |
| **Trial Statistical Centre** | **Prof Richard Woodman**  Flinders University  Sturt Road, Bedford Park. SA. 5042  E: [**richard.woodman@flinders.edu.au**](mailto:richard.woodman@flinders.edu.au) |
| **Trial Coordination Centre** | **TROG Cancer Research**  PO Box 88  Waratah  New South Wales 2289 Australia  **P: +61 (0)2 401 43911**  **F: +61 (0)2 401 43920**  **E:** [**SOCRATES_HCC@trog.com.au**](mailto:SOCRATES_HCC@trog.com.au) |
| **Sponsor** | **Trans Tasman Radiation Oncology Group**  (t/a TROG Cancer Research)  ABN: 45 132 672 292 |

# PROTOCOL AUTHORISATION

**TROG Scientific Committee (TSC);**

| A/Prof S Senthi | c/o PO Box 88, Waratah,  NSW 2289 Australia |  |
| --- | --- | --- |
|  |  | Sasha Senthi, TSC Chair |
|  |  |  |
|  |  | Date |

**Sponsor;**

| Trans Tasman Radiation Oncology Group | PO Box 88, Waratah,  NSW 2289 Australia |  |
| --- | --- | --- |
|  |  | Susan Goode, TROG CEO |
|  |  |  |
|  |  | Date |

**Trial Chair/s;**

| Prof Alan Wigg |  |  |
| --- | --- | --- |
|  |  | Alan Wigg |
|  |  |  |
|  |  | Date |
|  |  |  |
| A/Prof David Pryor |  |  |
|  |  | David Pryor |
|  |  |  |
|  |  | Date |
|  |  |  |

**TABLE OF CONTENTS**

[TRIAL PERSONNEL 3](#_Toc145487547)

[PROTOCOL AUTHORISATION 4](#_Toc145487548)

[LIST OF FIGURES 7](#_Toc145487549)

[LIST OF TABLES 7](#_Toc145487550)

[TRIAL EXECUTIVE 9](#_Toc145487551)

[TRIAL MANAGEMENT COMMITTEE 9](#_Toc145487552)

[ABBREVIATIONS 10](#_Toc145487553)

[TRIAL SUMMARY 12](#_Toc145487554)

[1 INTRODUCTION AND BACKGROUND 16](#_Toc145487555)

[1.1 Background 16](#_Toc145487556)

[1.2 Current Standard of Care 16](#_Toc145487557)

[1.3 Rationale to compare Stereotactic Ablative Body Radiotherapy (SABR) to the current standard of care (SoC) therapies. 16](#_Toc145487558)

[1.4 Stereotactic Ablative Body Radiotherapy (SABR) 17](#_Toc145487559)

[1.5 Rationale for choice of primary endpoint 19](#_Toc145487560)

[1.6 Challenges in response assessment in hepatocellular carcinoma and across modalities. 20](#_Toc145487561)

[1.7 Patient reported outcomes 21](#_Toc145487562)

[1.8 The SOCRATES Trial 21](#_Toc145487563)

[2 TRIAL OBJECTIVES 22](#_Toc145487564)

[2.1 Research Hypothesis 22](#_Toc145487565)

[2.2 Objectives 22](#_Toc145487566)

[3 TRIAL DESIGN 22](#_Toc145487567)

[4 PARTICIPANT SELECTION AND ELIGIBILITY 24](#_Toc145487568)

[4.1 Target population 24](#_Toc145487569)

[4.2 Accrual numbers and timelines 24](#_Toc145487570)

[4.3 Eligibility Criteria 24](#_Toc145487571)

[4.3.1 Inclusion criteria 24](#_Toc145487572)

[4.3.2 Exclusion criteria 24](#_Toc145487573)

[5 SCREENING 26](#_Toc145487574)

[5.1 Screening Log 26](#_Toc145487575)

[5.2 Informed Consent 26](#_Toc145487576)

[5.3 Participation in other research 26](#_Toc145487577)

[5.4 Methods for assigning treatment groups 26](#_Toc145487578)

[5.4.1 Cohort selection 26](#_Toc145487579)

[5.4.2 Randomisation Treatment Allocation 26](#_Toc145487580)

[5.5 Blinding 27](#_Toc145487581)

[6 TREATMENT INTERVENTIONS 28](#_Toc145487582)

[6.1 Percutaneous thermal ablation 28](#_Toc145487583)

[6.1.1 Cohort 1 Participants 28](#_Toc145487584)

[6.1.2 Cohort 2 participants 29](#_Toc145487585)

[6.2 Transarterial therapy 29](#_Toc145487586)

[6.2.1 QA measures for TACE/TARE in cohort 2 29](#_Toc145487587)

[6.3 Stereotactic Ablative Body Radiotherapy (SABR) 29](#_Toc145487588)

[6.3.1 Pre-trial Quality Assurance (QA) Program 29](#_Toc145487589)

[6.3.2 RT Treatment Prescription and Schedule 30](#_Toc145487590)

[6.3.3 Dose Criteria 30](#_Toc145487591)

[6.3.4 Target Volume Definitions 30](#_Toc145487592)

[6.3.5 Motion Management 30](#_Toc145487593)

[6.3.6 Treatment Delivery 30](#_Toc145487594)

[6.3.7 Treatment Verification 30](#_Toc145487595)

[6.3.8 On-Trial Radiotherapy Quality Assurance (RT QA) Program 31](#_Toc145487596)

[6.4 Concomitant and other treatments 31](#_Toc145487597)

[6.5 Treatment post progression 31](#_Toc145487598)

[7 TRIAL PARTICIPANT ASSESSMENTS 32](#_Toc145487599)

[7.1 Screening 32](#_Toc145487600)

[7.2 End of Treatment 33](#_Toc145487601)

[7.3 Follow-up 33](#_Toc145487602)

[7.3.1 4 weeks Post Treatment (+/- 1 week) 33](#_Toc145487603)

[7.3.2 Post Randomisation 33](#_Toc145487604)

[7.4 Progression 34](#_Toc145487605)

[7.5 Survival status 34](#_Toc145487606)

[7.6 Schedule of Assessments 34](#_Toc145487607)

[8 DISEASE PROGRESSION 35](#_Toc145487608)

[8.1 Definition of Progression 36](#_Toc145487609)

[8.1.1 Local progression 36](#_Toc145487610)

[8.1.2 New Lesions 36](#_Toc145487611)

[8.1.3 Summary of Response Assessment 36](#_Toc145487612)

[8.2 Definitions of (non-PD) target lesion responses 37](#_Toc145487613)

[8.3 Management of Disease progression 37](#_Toc145487614)

[9 PATIENT REPORTED OUTCOMES 38](#_Toc145487615)

[9.1 EORTC QLQ-C30 38](#_Toc145487616)

[9.2 EORTC QLQ-HCC18 38](#_Toc145487617)

[9.3 Non-English speaking patients 38](#_Toc145487618)

[9.4 Patient Reported Outcome Completion and Missing Data 38](#_Toc145487619)

[9.5 Completion of Patient Reported Outcome questionnaires 38](#_Toc145487620)

[10 QUALITATIVE REVIEW OF THE PATIENT EXPERIENCE 39](#_Toc145487621)

[10.1 Number of participants 39](#_Toc145487622)

[10.2 Recruitment and consent process 39](#_Toc145487623)

[10.3 Data collection 39](#_Toc145487624)

[10.4 Data Storage 40](#_Toc145487625)

[10.5 Analysis 40](#_Toc145487626)

[11 WITHDRAWAL FROM TREATMENT OR WITHDRAWAL FROM STUDY 40](#_Toc145487627)

[11.1 Discontinuation of a participant from treatment 40](#_Toc145487628)

[11.2 Withdrawal from Study 41](#_Toc145487629)

[11.3 Participant transfers 41](#_Toc145487630)

[12 TERMINATION OF THE STUDY 41](#_Toc145487631)

[13 SAFETY REPORTING AND MEDICAL MANAGEMENT 41](#_Toc145487632)

[13.1 Definitions 41](#_Toc145487633)

[13.2 Adverse event (AE) reporting 42](#_Toc145487634)

[13.2.1 Adverse Events (AE) 42](#_Toc145487635)

[13.2.2 Serious Adverse Events (SAE) 42](#_Toc145487636)

[13.3 New cancers 44](#_Toc145487637)

[14 STATISTICAL CONSIDERATIONS 45](#_Toc145487638)

[14.1 Sample Size Calculation 45](#_Toc145487639)

[14.2 Sequence Generation 45](#_Toc145487640)

[14.3 Endpoint Definitions 45](#_Toc145487641)

[14.4 Analysis population 45](#_Toc145487642)

[14.5 Definition of analyses sets 46](#_Toc145487643)

[14.5.1 Full analysis set 46](#_Toc145487644)

[14.5.2 Safety analysis set 46](#_Toc145487645)

[14.6 Statistical Analysis Plan 46](#_Toc145487646)

[14.6.1 Health economics analysis 46](#_Toc145487647)

[14.7 Secondary Analysis 47](#_Toc145487648)

[15 STUDY AND DATA MANAGEMENT 48](#_Toc145487649)

[15.1 Participating Centres 48](#_Toc145487650)

[15.2 Training of study site personnel 48](#_Toc145487651)

[15.2.1 Radiotherapy 48](#_Toc145487652)

[15.3 Monitoring of the study 48](#_Toc145487653)

[15.3.1 Site visits 48](#_Toc145487654)

[15.3.2 Audit or inspection 49](#_Toc145487655)

[15.3.3 Archiving of study documents 49](#_Toc145487656)

[15.3.4 Regulatory reviews 49](#_Toc145487657)

[15.4 Study timetable and end of study 49](#_Toc145487658)

[15.5 Data acquisition and management 49](#_Toc145487659)

[15.6 Security and back up of data 49](#_Toc145487660)

[15.7 Study Conduct Quality Assurance reviews 50](#_Toc145487661)

[15.7.1 Regulatory Reviews 50](#_Toc145487662)

[15.7.2 Informed Consent 50](#_Toc145487663)

[15.7.3 Eligibility Reviews 50](#_Toc145487664)

[15.7.4 Progression Reviews 50](#_Toc145487665)

[16 RESEARCH GOVERNANCE 50](#_Toc145487666)

[16.1 Sponsor and Funder 50](#_Toc145487667)

[16.2 Trial Chairpersons 50](#_Toc145487668)

[16.3 Trial Executive Committee and the Trial Management Committee 50](#_Toc145487669)

[16.4 Independent Data Safety Monitoring Committee 50](#_Toc145487670)

[16.5 Principal Investigator 51](#_Toc145487671)

[16.6 Consumer Representative 51](#_Toc145487672)

[17 PATIENT PROTECTION AND ETHICAL CONSIDERATIONS 51](#_Toc145487673)

[17.1 Ethical Principles and Regulatory Compliance 51](#_Toc145487674)

[17.2 Adherence to Protocol 51](#_Toc145487675)

[17.3 Cultural values and principles 51](#_Toc145487676)

[17.4 Confidentiality 51](#_Toc145487677)

[17.5 Informed consent 52](#_Toc145487678)

[17.6 Insurance and compensation 52](#_Toc145487679)

[18 PUBLICATION AND PRESENTATION POLICY 52](#_Toc145487680)

[19 REFERENCES 53](#_Toc145487681)

[APPENDIX 1: Child-Pugh score & Barcelona Clinic Liver Cancer (BCLC) staging system 56](#_Toc145487682)

[A1.1 Child-Pugh score 56](#_Toc145487683)

[A1.2 Barcelona Clinic Liver Cancer (BCLC) staging system 56](#_Toc145487684)

[APPENDIX 2: ECOG Performance Status 57](#_Toc145487685)

[APPENDIX 3: Imaging Guidelines 58](#_Toc145487686)

[A3.1 Tri/quad-phasic CT protocol 58](#_Toc145487687)

[A3.2 Triphasic MRI of the Liver 58](#_Toc145487688)

[APPENDIX 4: mRECIST for Hepatocellular Carcinoma 60](#_Toc145487689)

[APPENDIX 5: Qualitative Review of the patient experience of non-operative treatment in early-stage hepatocellular carcinoma: Example interview script 61](#_Toc145487690)

# LIST OF FIGURES

[**Figure 1: Study Schema** 15](#_Toc145487691)

[**Figure 2: mRECIST assessment of tumour lesions at baseline** 60](#_Toc145487692)

[**Figure 3: mRECIST assessment of tumour response** 60](#_Toc145487693)

# LIST OF TABLES

[**Table 1: Summary of clinical studies reporting outcomes after SABR or PA in patients with early-stage HCC** 18](#_Toc145487694)

[**Table 2: Objectives and Endpoints** 22](#_Toc145487695)

[**Table 3: Recommended SABR Dosing Schedules** 30](#_Toc145487696)

[**Table 4: Schedule of Assessments** 34](#_Toc145487697)

[**Table 5: Definition of Local Progression by Cohort** 36](#_Toc145487698)

[**Table 6: Summary of Response Assessment** 36](#_Toc145487699)

[**Table 7: Definitions of causality** 43](#_Toc145487700)

[**Table 8: Endpoint definitions** 45](#_Toc145487701)

[**Table 9: Cultural principles** 51](#_Toc145487702)

# TRIAL EXECUTIVE

| **Name** | **Role** | **Affiliation** |
| --- | --- | --- |
| Prof Alan Wigg | Trial Chairperson | Flinders Medical Centre |
| A/Prof David Pryor | Trial Chairperson | Metro South Hospital and Health Service |
| Dr Jonathan Tibballs | Interventional Radiologist | Sir Charles Gairdner Hospital |
| Dr Katherine Stuart | Hepatologist | Metro South Hospital and Health Service |
| Prof Stuart Roberts | Hepatologist | Alfred Health |
| A/Prof Hien Le | Radiation Oncologist | Royal Adelaide Hospital |
| Prof Annette Haworth | Medical Physicist | University of Sydney |
| Prof Richard Woodman | Statistician | Flinders University |
| A/Prof Richard De Abreu Lourenco | Health Economist | CREST, University of Technology Sydney |

# TRIAL MANAGEMENT COMMITTEE

The Trial Management Committee (TMC) will be constituted from the members of the Trial executive and will include identified collaborators, members of the TROG central operations office, Principal Investigators and other key study personnel, to ensure representation from a range of sites and professional groups. Where possible, membership will include consumer group representation.

A copy of the current membership of the TMC can be obtained from the trial management team at TROG Cancer Research.

# ABBREVIATIONS

| 3D | 3-dimensional |
| --- | --- |
| BCLC | Barcelona Clinic Liver Cancer staging |
| CRF | Case Report Forms |
| CT | Computerised Tomography |
| CP | Child Pugh score |
| CTCAE | Common Terminology Criteria for Adverse Events |
| CTRA | Clinical Trial Research Agreement |
| CTV | Clinical Target Volume |
| DEB | Drug Eluting Bead |
| ECOG | Eastern Co-operative Oncology Group |
| FBC | Full blood count |
| FFLP | Freedom from local progression rate |
| GCP | Good Clinical Practice |
| GTV | Gross Tumour Volume |
| HCC | Hepatocellular Carcinoma |
| Hgb | Haemoglobin |
| HR | Hazard ratio |
| HREC | Human Research Ethics Committee |
| HRQoL | Health-related Quality of Life |
| IDSMC | Independent Safety Data Monitoring Committee |
| LTC | Local tumour control rate |
| MRI | Magnetic Resonance Imaging |
| MWA | Microwave ablation |
| NCI | National Cancer Institute |
| OAR | Organ at Risk |
| OS | Overall survival |
| PA | Percutaneous ablation |
| Plts | Platelets |
| PRO | Patient Reported Outcome |
| PTV | Planning Tumour Volumes |
| QA | Quality Assurance |
| QALY | Quality-adjusted life year |
| RCT | Randomised controlled trial |
| RFA | Radiofrequency ablation |
| RT | Radiation Therapy |
| SAE | Serious Adverse Event |
| SABR | Stereotactic ablative body radiotherapy |
| SM | Stage migration |
| SOC | Standard of Care |
| TACE | Transarterial chemoembolisation |
| TARE | Transarterial radioembolisation |
| TCC | Trial Coordinating Centre |
| TMC | Trial Management Committee |
| TSC | TROG Scientific Committee |

# TRIAL SUMMARY

| **Data category** | **Information** | |
| --- | --- | --- |
| **Protocol title** | **A randomised controlled trial of Standard Of Care versus RadioAblaTion in Early Stage HepatoCellular Carcinoma** | |
| **Synopsis** | Current standard of care (SOC) first line therapy for patients with inoperable, early stage, solitary, ≤3cm hepatocellular carcinoma (HCC) not planned for transplant is thermal ablation with microwave (MWA) or radiofrequency (RFA). However, for larger tumours or tumours in difficult anatomical locations thermal ablation may be contraindicated or associated with higher rates of local recurrence. This leads to treatment stage migration with non-curative therapies such as transarterial chemo-embolisation (TACE). Stereotactic ablative body radiotherapy (SABR) is an alternative treatment for inoperable early stage HCC with few contraindications to therapy and reported local control rates in the order of 90% at 2 years. SABR is increasingly used for patients with recurrent HCC not suitable for or progressing after ablation or transarterial therapies and in some centres is now also considered a standard first line treatment option, however, high level evidence is lacking. The SOCRATES study will compare SABR to other current SOC treatments in the first line setting for non-surgical candidates with solitary, early stage  HCC. | |
| **Trial Registry** | Registry Name | [anzctr.org.au](https://www.anzctr.org.au/Trial/Registration/TrialReview.aspx?id=381818&isReview=true) |
|  | Trial Identifying; | ACTRN12621001444875p |
|  | Date of registration | 21 October 2021 |
| **Universal Trial Number** | - | |
| **Secondary identifying numbers** | TROG 21.07 | |
| **Source(s) of monetary or material support** | MRFF Clinical Trials Grant  (Rare Cancers Rare Diseases and Unmet Need) | |
| **Primary sponsor contact details** | TROG Cancer Research  PO Box 88 Waratah, NSW, 2298  +61 2 40143911 | |
| **Collaborators and Supporters** | Australasian Gastro-Intestinal Trials Group (AGITG)  Abdominal Radiology Group of Australia and New Zealand (ARGANZ)  Gastrointestinal Society of Australia (GESA) | |
| **Contact for public queries** | Clinical Research Associate  +61 2 40143911 | |
| **Contact for scientific queries** | Prof Alan Wigg  E: [Alan.wigg@sa.gov.au](mailto:Alan.wigg@sa.gov.au) | |
| **Countries of recruitment** | Australia | |
| **Health condition(s) or problem(s) studied** | Newly diagnosed early stage (BCLC 0/A) HCC with solitary ≤8 cm tumour, ineligible for surgical resection and not planned for transplantation. | |
| **Intervention(s)** | Percutaneous Thermal Ablation (MWA/RFA)  Transarterial Therapies (TACE/TARE)  Stereotactic Ablative Body Radiotherapy (SABR) | |
| **Eligibility criteria** | **Inclusion**   - Histological or radiological diagnosis of single, new HCC with largest diameter ≤8 cm (BCLC stage 0 or A).   1. If prior history of HCC, the prior HCC must have been:      - Early stage, solitary HCC, ≤5 cm in size **and**,      - Have arisen within a different liver segment to the current HCC **and,**      - Treated with curative intent therapy>2 years prior with no evidence of activedisease at the site. - As per local multidisciplinary meeting consensus patient is suitable for percutaneous thermal ablation and/or transarterial therapies **and** is not suitable for or declined resection **and** not planned for transplant. - Child-Pugh score ≤B7 with no or diuretic-controlled ascites - ECOG performance status ≤2 - Platelets ≥50x10^9^/L, Haemoglobin ≥80 g/L, Neutrophils ≥1.0x10^9^/L, INR <1.8 (except if on therapeutic anticoagulation) - 18 years of age or older and able to provide written consent | |
|  | **Exclusion**   - Presence of multifocal HCC, macrovascular invasion or extrahepatic disease - Clinically evident ascites or hepatic encephalopathy - Prior abdominal radiation therapy that would preclude the delivery of protocol defined SABR to the tumour. - Prior treatment for any HCC within last 2years. - Known additional invasive malignancy (excluding non-melanoma skin cancer) that is progressing or required treatment within the last 2 years. - Untreated Hepatitis B or C - Pregnancy | |
| **Study type** | Prospective, parallel and open-label randomised control trial | |
| **Phase** | II | |
| **Target sample size** | **Cohort 1**: HCC ≤ 3cm **and** eligible for percutaneous thermal ablation (SABR v MWA/RFA) = 118  **Cohort 2:** HCC > 3cm **or** ≤ 3cm ineligible for percutaneous thermal ablation (SABR v SOC) = 100 | |
| **Primary objectives** | To evaluate 2 year freedom from local progression (FFLP) for SABR versus thermal ablation in Cohort 1 and SABR versus standard of care (transarterial therapies and/or thermal ablation) in Cohort 2. | |
| **Key secondary objectives** | 1. 2-year progression-free survival (PFS) 2. 2-year overall survival (OS) 3. Safety and adverse events (CTCAE v5.0) 4. Patient reported outcomes (EORTC QLQ-C30, QLQ-HCC18) 5. Patient treatment experience assessment 6. Cost-effectiveness analysis | |
| **Statistical considerations** | **Cohort 1** (randomised 1:1 SABR versus thermal ablation) will have 80% power to detect a difference of 20% in 2-year FFLP between SABR (95%) and thermal ablation (75%). Assuming a 2-sided type 1 error rate of alpha=0.05, 47 evaluable patients per arm will be required. A total of 118 patients will be randomised to allow for up to 24 patients not being evaluable for the primary endpoint (assuming a drop-out rate of up to 10% and a 2-year mortality rate of 10%)  **Cohort 2** (randomised 1:1 to SABR versus SOC), will have 80% power to detect a difference of 25% in 2-year FFLP between SABR (90%) and transarterial therapies (65%). Assuming a 2-sided type 1 error rate of alpha=0.05, 40 evaluable patients per arm will be required. A total of 100 patients will be randomised to allow for 20 patients not being evaluable for the primary endpoint (assuming a drop-out rate of 10% and a 2-year mortality rate of 10%). | |
| **Plain Language Summary** | This study will investigate whether a radiotherapy technique (called SABR) can treat early stage liver cancer more effectively than current treatments which use heating probes directly inserted into the tumour or chemotherapy or radioactive particles injected into the blood supply of the tumour. | |

**Figure 1: Study Schema**


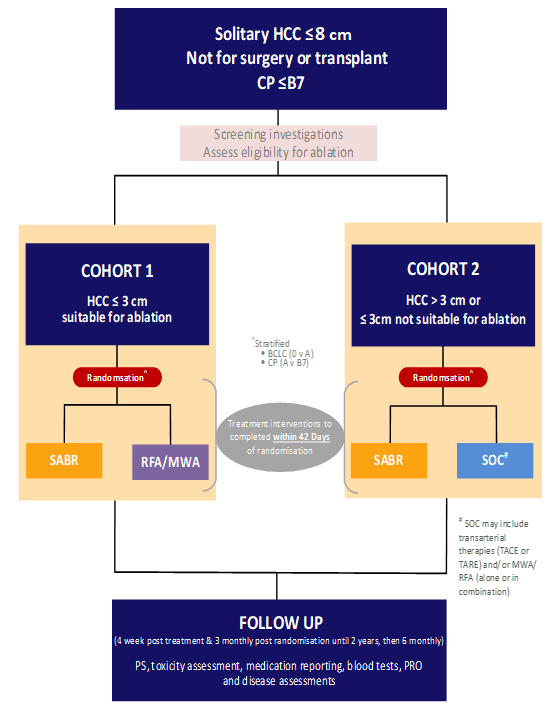


HCC: Hepatocellular carcinoma | CP: Child Pugh score | BCLC: Barcelona Clinic Liver Cancer staging |

RFA: Radiofrequency Ablation | MWA: Microwave ablation | SABR: Stereotactic Ablative Body Radiotherapy |

SOC: Standard of Care | TACE: Transarterial chemoembolization |TARE: Transarterial radioembolization |

PS: Performance Status | PRO: Patient Reported Outcomes

# INTRODUCTION AND BACKGROUND

## Background

Liver cancer is the fourth leading cause of cancer-related death globally, mainly accounted for by hepatocellular carcinoma (HCC).(1, 2) Over the last 40 years, the incidence rate in Australia has jumped by almost eight-fold, from 1.37 per 100 000 in 1982 to 8.60 per 100 000 in 2019, corresponding to the fastest rising incidence and mortality rate of any cancer in Australia.(3, 4) It is the only low survival cancer with a rapidly rising incidence. Survival rates for advanced disease are poor and treatment options for early stage disease can be limited.

The treatment strategy in patients with HCC is based on tumour stage, underlying liver function and performance status.(5) The Barcelona Clinic Liver Cancer (BCLC 0/A) staging system is commonly used to guide prognostication and treatment.(6) For early-stage HCC (BCLC stage 0/A), curative surgical therapies such as liver transplantation and liver resection are recommended to achieve the best survival outcomes, however, less than 30% of patients are candidates for surgery upon diagnosis.(5-8).

## Current Standard of Care

Thermal ablation using microwave (MWA) or radiofrequency (RFA) is the current standard curative therapy for non-surgical candidates with early-stage HCC ≤ 3cm. However, many patients with a single HCC nodule are not suitable for thermal ablation and undergo so called “treatment stage migration”. (9, 10) For these patients the current standard of care migrates to transarterial therapies traditionally considered as non-curative, such as transarterial chemoembolization (TACE) or transarterial radioembolization (TARE). More recently, the combination of TACE and radiofrequency ablation RFA has shown improved overall and recurrence free survival compared to RFA alone, most evident for the cohort with HCC >3cm. (11)

## Rationale to compare Stereotactic Ablative Body Radiotherapy (SABR) to the current standard of care (SoC) therapies.

Thermal ablation is the preferred first line therapy for inoperable, solitary, early-stage HCC ≤ 3cm, however, there are two main limitations.

Firstly, the rate of contraindications to thermal ablation for patients with early-stage HCC can be high, and often underappreciated in real-world practice, as reported in several retrospective studies where 34-57% of patients did not undergo ablative therapies.(9, 10) Larger tumour size (>3cm) and certain anatomical locations (subphrenic, subcapsular, in close proximity to large vessels or central biliary structures) have been associated with higher local failure rates and increased complication rates. (12-14) Technical factors related to real-time image localisation or patient specific comorbidities may also come in to play. In a retrospective multicentre study of 245 patients with early-stage (BCLC 0/A) HCCs referred to multidisciplinary clinics within South Australia, 140 (57%) had contraindications to thermal ablation.(15) The main reasons cited were difficult/high risk tumour locations (66 patients) and tumour size >3 cm (65 lesions). A further Australian multicentre study of 253 BCLC early stage HCC patients in Victoria found 41.5% of patients underwent treatment stage migration to therapies such as TACE.(10) Treatment stage migration to non-curative therapies such as TACE is associated with lower local control rates at 2 years, in the order of 23 - 40%.(10, 16-18) A recent retrospective study of high dose TARE in early stage disease, however, reported very low rates (<10%) of local progression.(19) Given the retrospective nature of these studies there is likely significant bias limiting between treatment comparisons. SOCRATES will prospectively evaluate the propotion of non-surgical candidates with solitary HCC who undergo treatment stage migration. In this population of patients with HCC >3cm or HCC ≤ 3cm but ineligible for ablation **(Cohort 2)**, SOCRATES will evaluate the efficacy of modern transarterial therapy (inlcuding the option of combined TACE-ablation) compared to SABR in the first line setting within the context of a prospective randomised controlled trial.

Secondly, reported rates of local recurrence following thermal ablation are highly variable, ranging from <10% to >30% at 2 to 3 years. (12, 15, 20-26) Previous randomised controlled trials of resection versus RFA and of MWA versus RFA have reported local recurrence rates of between 5% to 12%.(24, 26) The multicentre SURF study from Japan randomised 301 patients (median tumour size 1.8 cm) with early stage disease to resection or RFA and reported local recurrences in 8% of the surgical cohort and 16% of the RFA cohort.(27) Tumour size >2cm was associated with a higher risk of local recurrence after RFA. A single centre experience from the University of Toronto using RFA as first-line therapy for <3cm HCC reported local recurrence as the first site of relapse in 16% of patients (12% if <2cm and 20% if >2cm).(20) Multicentre, retrospective case series from Australian centres have reported local recurrence rates ranging from 23% at 14 months to 37% at 3 years.(10, 15) The variability in reported local control rates is likely multifactorial and related to issues of patient selection (tumour size, location), modality (MWA v RFA ), operator technique and experience, duration of follow-up and endpoint definitions. For patients with tumours ≤ 3cm and deemed suitable for percutaneous thermal ablation **(Cohort 1)**, SOCRATES aims to prospectively and comprehensively evaluate the efficacy, toxicity, health related quality of life and cost-effectiveness of modern thermal ablation techniques compared to SABR.

## Stereotactic Ablative Body Radiotherapy (SABR)

SABR is a significant advance from traditional external beam radiotherapy techniques that enables high radiation doses to be delivered to small target volumes with higher precision and in fewer treatment sessions (typically 3 to 5). SABR can treat larger tumours with similarly high local control rates and those abutting the diaphragm, liver capsule, major blood vessels and bile ducts. (14, 28, 29) Tumours abutting the stomach or bowel can also be treated with appropriate motion management and dose reduction at the tumour/bowel interface.(30) Whilst SABR aims to confine the high radiation doses to the ablation zone, there is a zone of intermediate and low dose fall-off with the potential advantage of treating adjacent microsopic satellites. Further advantages lie in its non-invasive nature and routine integration of onboard stereotactic 3D and 4D image guidance for robust target localisation and verification of treatment accuracy.

Recent non-randomised studies and systematic reviews investigating the efficacy of SABR in BCLC 0/A HCC patients are outlined in **Table *1***.(14, 28, 29, 31-38) These studies report high local control rates (>90% at 3 years) and similar survival outcomes in comparison to thermal ablation.(14, 31, 32, 37, 39) In these studies, SABR has a low reported rate of grade ≥3 toxicities (≤5%) and the risk of classical radiation induced liver disease (RILD) in patients with well-compensated liver function (Child-Pugh A) is low (<1-5%).(23, 28, 29, 33-36, 38, 40-42)

In the recurrent setting, one randomised study demonstrated non-inferior 2-year local progression free survival for proton beam radiotherapy (PBT) compared to RFA. (23) In this study 144 patients with 1 or 2 recurrent nodules (<3cm) after prior therapy were randomised at the point of diagnosis of recurrence to receive either RFA or PBT (66 Gy equivalent in 10 fractions). Of the 72 patients initially randomised to RFA, 22 (31%) were subsequently deemed technically unsuitable for RFA and underwent PBT (n=19) or TACE (n=3), in keeping with the above mentioned treatment stage migration data. In the proton therapy arm 9 patients (12.5%) were unable to undergo PBT (generally due to concern regarding proximity to bowel) and received RFA (n=6) or other therapies. In both the per protocol (RFA n = 56 and PBT n=80) and intention to treat populations PBT had a higher 2-year local progression free survival (94.8% PP and 92.8% ITT) than RFA (83.9% PP and 83.2% ITT). Although grade 3 AEs occurred more frequently in the RFA group than the PBT group (16.1% *vs.* 0%, *p <*0.001), these toxicities were transient and all patients recovered. In both arms, treatment-related late hepatic failure and death without evidence of disease progression and/or subsequent treatment were not observed.

Despite promising non-randomised data and increasing utilisation, there are currently no randomised studies comparing SABR with SOC therapies in the first line (i.e. treatment naïve) setting for inoperable, early-stage HCC. A small number of non-randomised studies comparing outcomes between thermal ablation and SABR for early stage HCC are summarised in **Table *1***. In the majority of these studies SABR was associated with high local control rates, particularly for tumours >2cm. Three studies reported lower local control rates with thermal ablation for the subgroups with subphrenic and perivascular locations.(14, 32, 39) The interpretation of this data is hampered by lack of randomisation and therefore the effects of bias (from known and unknown factors) remain difficult to exclude. Whether there is a true difference in local control between SABR and SOC therapies is therefore unknown at present.

**Table 1: Summary of clinical studies reporting outcomes after SABR or PA in patients with early-stage HCC**

| **Study (Year)** | **Study Type** | **N** | **Study cohort/ Inclusion criteria** | **Intervention** | **Primary/ Secondary outcomes +/- significant predictors of LTC/ OS** |
| --- | --- | --- | --- | --- | --- |
| Dobrzycka et al. (2019) | Meta-analysis | 973 | 16 observational studies analysing SABR in early-stage HCC (single tumour ≤5cm or ≤3 lesions each ≤3cm, without macrovascular invasion or metastasis; CP A or B) | SABR | LTC: 94% (1-year); 92% (2-year) 93% (3-year)  OS: 90.9% (1-year); 67.5% (2-year); 73.4% (3-year)  Grade ≥3 toxicities: 5.3% |
| Rim et al. (2019) | Meta-analysis | 1950 | 32 observational studies analysing SABR in early to advanced stage HCC (with vascular invasion and/or extrahepatic metastasis) | SABR | Size <5cm: LTC: 91.0% (1-year); 86.3% (3-year)  Size <5cm: OS: 79.5% (1-year); 51.9% (3-year) |
| Park et al. (2020) | Retrospective review | 290 | Single centre, retrospective analysis of SABR in small HCC (<6 cm, ≤3 lesions, no vascular invasion or metastasis) | SABR | Size ≤3cm: LTC 93.3% (5-year)  Size >3cm: LTC 76.5% (5-year)  OS: 45% (5-year, whole cohort) |
| Liu et al. (2020) | Retrospective review | 96 | Single centre, retrospective analysis of SABR in early to advanced stage HCC | SABR | BCLC 0/A: FFLP: 94% (18 months)  BCLC 0/A: OS: 95% (12 months) |
| Mathew et al. (2020) | Prospective/ Retrospective review | 297 | Multicentre analysis of SABR in early-stage HCC without vascular invasion or metastasis, including large lesion >5cm (median size 2.7cm, range 0.5-18.1cm) | SABR | LTC: 93.4% (1-year); 86.7% (3-year)  Subgroup analysis- size <3cm: LTC 90% (3-year)  OS: 77% (1-year); 39% (3-year) |
| Yoon et al. (2020) | Phase II clinical trial | 50 | Single centre, single arm trial for early-stage HCC (single lesion ≤5cm, or ≤3 lesions with a sum of total diameter ≤5cm) | SABR | LTC: 100% (2-year); 97% (5-year)  OS: 96% (2-year); 78% (5-year) |
| Kubo et al. (2018) | Retrospective review | 65 | Single centre, retrospective analysis of SABR in BCLC 0/A HCC, <3 HCC, each >5cm | SABR | LTC: 100% (3- & 5-year)  OS: 56% (3-year) |
| ***Compared to RFA*** | | | | | |
| Kim et al. (2020) | Retrospective review | 2064 | Multicentre, retrospective analysis with propensity score matching comparing SABR versus RFA in early HCC (≤6cm, ≤3 lesions with a sum of total diameter (≤6cm, no vascular invasion/ metastasis) | SABR or RFA | LTC: 83.6% SABR versus 68.9% RFA (2-year)  Subgroup analysis- superior LTC with SABR in small tumours (≤3 cm) irrespective of location (HR 0.33)  OS: 22.4% SABR versus 28.9% RFA (2-year) |
| Hara et al. (2019) | Retrospective review | 374 | Multicentre, retrospective analysis with propensity score matching comparing SABR versus RFA in early HCC (≤3cm, ≤3 lesions, no vascular invasion/ metastasis) | SABR or RFA | LTC: 93.6% SABR versus 79.8% RFA (3-year)  OS: 70.4%SABR versus 69.1%RFA (3-year) |
| Kim et al. (2019) | Retrospective review | 668 | Single centre, retrospective analysis with propensity score matching comparing SABR versus RFA for inoperable HCC (88% BCLC 0/A) | SABR or RFA | LTC: 74.9% SABR versus 64.9% RFA (2-year)  Subgroup analysis- Decreased LTC for HCC ≥2cm and subphrenic location with RFA (HR 1.7) but not SABR  OS: 71.8% SABR versus 76.4%RFA (2-year) |
| Wahl et al. (2016) | Retrospective review | 224 | Single centre, retrospective analysis of SABR versus RFA for inoperable, non-metastatic HCC | SABR or RFA | LTC: 83.8% SABR versus 80.2% RFA (2-year)  Subgroup analysis- Decreased LTC for HCC ≥2cm with RFA (HR 3.35) but not SABR  OS: 46% SABR versus 53% RFA (2-year) |
| Jeong Y et al. (2021) | Retrospective review | 226 | Single centre, retrospective analysis of SABR (n=87 patients) versus RFA (n=179) in small (≤3 cm) HCC with inverse probability treatment weighting to adjust for imbalances | SABR or RFA | LTC: 96.3% SABR vs 90.6% RFA (4-year)  Subgroup analysis: poorer LTC for RFA if HCC in perivascular location.  OS: 70.2% SABR vs 71.8% RFA (4-year) |
| ***Retrospective analysis of local recurrence rate after PA using our local institutions data*** | | | | | |
| Chinnaratha et al. (2015) | Retrospective review | 126 | Multicentre, retrospective analysis of PA for early HCC (single ≤5cm or ≤3 lesions each <3cm) | RFA | Local recurrence: 23.4% (mean FU 14 months)  RFS: 30% (3-year) |
| Loo et al. (2020) | Retrospective review | 245 | Multicentre, retrospective analysis PA outcomes and stage migration therapies in treatment naïve, early HCC (single ≤3cm or ≤3 lesions each <3cm | RFA vs SM therapies | LTC: 71% (2-year); 63.1% (3-year) PA versus 28% (2-year) 19% SM therapies  OS: 80% (2-year); 71% (3-year) PA versus 52% (2-year) 40% (3-year) SM therapies |

*FFLP: Freedom from local progression rate, HR: Hazard ratio, LTC: local tumour control rate, OS: overall survival rate, RCT: randomised controlled trial, SM: stage migration*

## Rationale for choice of primary endpoint

The aim of any local therapy is to maximise local control whilst minimising toxicity and any impact on functional reserve. This study is powered to detect a clinically relevant improvement in the primary endpoint of 2 year freedom from local progression (FFLP) with progression free survival (PFS), overall survival (OS), toxicity and patient reported outcomes representing important secondary endpoints. Whilst overall survival is considered the most robust efficacy endpoint for an intervention it can be confounded by the multitude of effective downstream therapies available and their subsequent effects on progression of tumour and underlying liver dysfunction.(43, 44) This is particularly the case when evaluating therapies in early stage HCC. For HCC multiple sequential local therapies are often employed whilst the disease remains in an early stage and hence, the trial investigators consider it impractical to limit post-progression therapy, including the option of cross-over therapy in the event of local or other intra-hepatic progression. Demonstration of a significant improvement in local control without increased toxicity within the context of a randomised trial would provide evidence to support the inclusion of SABR as an initial treatment option for single HCC (BCLC-O/A).

## Challenges in response assessment in hepatocellular carcinoma and across modalities.

Assessment of response and progression poses a number of challenges in the setting of local therapies for early stage HCC.(43, 44) For so called “typical” lesions that display intratumoural (non-rim like) arterial enhancement, complete necrosis of the tumour (as evidenced by a lack of arterial enhancement) following local therapies may be scored as stable disease (SD) via RECIST v1.1 or even progressive disease (PD) if cystic swelling of the necrotic lesion occurs. For thermal ablation, the post-ablation cavity is typically larger than the baseline RECIST v1.1 tumour measurement. Furthermore, the presence of regenerative nodules, post-therapy perfusional changes, ascites, pleural effusions and enlarged peri-portal lymph nodes can all occur in the context of underlying cirrhosis and/or prior therapies in the absence of true disease progression. The modified RECIST (mRECIST) criteria were developed to address the above issues and reduce the overreporting of progressive disease. (43)

mRECIST takes into account the events that can be attributable to the natural history of cirrhosis as above and also introduced the concept of “viable” tumour for response assessment of target lesions. It defines “typical” intrahepatic HCC lesions as ones that demonstrate intratumoural (*i.e.* non-rim-like) arterial enhancement on contrast-enhanced CT or MRI. For typical lesions mRECIST recommends measuring the longest diameter of viable tumour (i.e. any persistent enhancement) instead of overall lesion diameter. Target lesion progression using mRECIST criteria requires at least a 20% increase (compared to nadir) in longest diameter of viable arterial enhancing tumour with an absolute increase of ≥5mm. This measurement should exclude intervening areas of complete necrosis. A new typical intrahepatic lesion must be ≥1cm. This has been shown to be a more accurate predictor of response and progression for typical lesions treated with thermal ablation or TACE. (43)

“Atypical” intrahepatic lesions are defined in mRECIST as non-enhancing or rim enhancing lesions. These features can be seen in well differentiated, steatotic or poorly differentiated tumours and have often undergone biopsy confirmation. For these lesions RECIST v1.1 criteria, i.e. overall tumour diameter measurements, should apply. A new atypical intrahepatic lesion is regarded as equivocal until interval growth ≥10mm is demonstrated on subsequent scans.

Whilst mRECIST has advantages for response assessment in the setting of thermal ablation, TACE and some systemic therapies, the distinct mechanism of action of radiation-based therapies introduces further challenges. For radiation-based therapies such as radio-embolisation and SABR, both persistent arterial phase hyperenhancement within the target lesion and geographic arterial phase hyperenhancement without washout in the adjacent parenchyma are common in the first 3-12 months following treatment.(45) In contrast to thermal ablation or TACE, residual enhancement in this period is not predictive of subsequent local progression. (46)

The distinct natural progression of target lesion and adjacent parenchymal changes following SABR has been illustrated by Mendiratta-Lala and colleagues.(46) In the first 3 to 12 months following SABR, the arterial phase hyperenhancement gradually converts to portal venous and delayed phase hyperenhancement, as radiation induced veno-occlusion and fibrosis develops, with overlying capsular retraction. Lack of washout or other features such as diffusion restriction can help differentiate expected post-radiation perfusional changes from infiltrative tumour. Radiation-induced biliary strictures, and their associated distal segmental changes, can develop after SABR for more central tumours. Despite mRECIST response having a lower predictive ability in the early months following SABR, with the majority demonstrating stable disease, subsequent new nodular or increasing volume and intensity of arterial phase hyperenhancement with washout are indicators of progressive disease.

Despite the above challenges in assigning various categories for response such as stable disease, partial or complete response, the endpoint of progressive disease appears to be more reliable. The primary endpoint of the SOCRATES study will be FFLP with local progression defined as target lesion progression as per mRECIST criteria (viable tumour diameter for typical lesions, lesion diameter for atypical lesions). Given the potential for more complex vascular changes following radiation-based therapies, RECIST v1.1 measurements will also be taken and local progression events will also include progression on RECIST v1.1. criteria. For the thermal ablation cohort, the post-ablation cavity will serve as baseline for RECIST v1.1 measurements. Central, independent radiological review will be undertaken for progression events with equivocal findings or non-consensus regarding progression requiring interval imaging to confirm progression (backdated to the previous imaging time point).

## Patient reported outcomes

Given the increasing number of therapies available for early stage HCC, evaluation of patient reported outcomes (PROs) can provide valuable additional insights into the relative tolerability profile of these therapies. A scoping review of PROs in HCC by the American Association for the Study of Liver Diseases (AASLD) has highlighted a paucity of high-quality data, in particular for locoregional therapies (TACE/TARE) and radiotherapy in HCC. (47) The majority of studies to date have consisted of small, single-centre cohorts.The European Organization for Research and Treatment of Cancer (EORTC) Quality of Life Questionnaire (EORTC QLQ-C30) is a well validated general PRO measure and will provide valuable information on general health related quality of life (HRQoL) and health utility across the treatments.(48) In addition, the EORTC QLQ-C30 can also be used to assess quality of life weights for inclusion in assessments of relative treatment value as part of cost-effectiveness analyses(49). The EORTC Hepatocellular Carcinoma 18-question module (EORTC QLQ-HCC18) was developed specifically to assess symptom burden and impact on HRQoL in people with HCC. (50) It has been validated previously in advanced and pre-treated, unresectable HCC cohorts with estimates of meaningful within patient changes developed.(51) However, data specifically on first line therapy for early stage HCC and in particular comparisons between SABR and current first-line therapies are lacking.

## The SOCRATES Trial

Despite encouraging randomised and non-randomised data and it’s increasing use in a number of centres around Australia, SABR is currently not recommended as a first-line therapy option for early-stage HCC in most HCC guidelines owing to a lack of randomised studies. (5, 6, 52) High-quality randomised controlled trials of SABR in patients with early-stage HCC who are non-surgical candidates, are important to compare efficacy, toxicity, patient acceptability and cost-effectiveness with the current SOC. The SOCRATES randomised controlled trial will assess the efficacy, toxicity, HRQoL profile and cost effectiveness of SABR compared to the current SOC treatment in patients with first diagnosis of unresectable, single nodule HCC.

# TRIAL OBJECTIVES

## Research Hypothesis

SABR will result in higher rates of FFLP at 2 years compared to percutaneous thermal ablation ± TACE or transarterial therapies (TACE/TARE).

## Objectives

**Table 2: Objectives and Endpoints**

|  | **OBJECTIVE** | **ENDPOINT** |
| --- | --- | --- |
| **PRIMARY** | Evaluate the efficacy of SABR compared to SOC first line therapies in terms of local lesion control | 2-year FFLP according to mRECIST ( or RECIST v1.1 when mRECIST can’t be used) |
| **SECONDARY** | Evaluate progression-free survival | 2-year PFS |
|  | Evaluate overall survival | 2-year OS |
|  | Evaluate safety and adverse events | Cumulative incidence of adverse events (CTCAE v5) |
|  | Compare patient reported outcomes between SABR and SOC therapies | EORTC QLQ-C30 and QLQ-HCC18 - mean scores over time. Time to ≥10 point decrease in the combined GHS/QoL score. |
|  | Cost-effectiveness analysis | Incremental cost per outcome (additional patient with FFLP at 2 years; and quality adjusted life years) for SABR compared with SoC. |
|  | Qualitative review of patient experience | Patient treatment experience assessed by qualitative methods |
| **EXPLORATORY** | To identify biomarkers associated with treatment outcome. | Changes in serum AFP, haematological and liver function parameters post-treatment. |

# TRIAL DESIGN

The study is designed as a phase II, prospective, randomised, parallel and open-label, multi-institutional superiority trial for patients with newly diagnosed early stage (BCLC 0/A) , solitary ≤8cm nodule HCC, ineligible for surgical resection or transplantation.

Patients with tumours ≤ 3cm **and** eligible percutaneous thermal ablation will be randomly assigned (1:1 ratio) to either thermal ablation or SABR.

Patients with tumours ≤ 3cm assessed as ineligible for thermal ablation or patients with tumours >3cm will be randomly assigned (1:1 ratio) to either SABR or standard of care which can include transarterial therapies (TACE or TARE) and or percutaneous thermal ablation (MWA or RFA). See **Figure 1: Study Schema.**

# PARTICIPANT SELECTION AND ELIGIBILITY

## Target population

The target population includes any newly diagnosed, early stage (BCLC 0/A) HCC with solitary ≤8 cm nodule, ineligible for or declined liver resection, not planned for transplantation and with clinically compensated cirrhosis (Child-Pugh score ≤B7).

## Accrual numbers and timelines

Target accrual is 118 participants for Cohort 1 and 100 for Cohort 2. Participant enrolment is anticipated to take 2 years. The total duration of this study with assigned treatment and follow-up is estimated to be around 5 years, which includes obtaining ethics and governance approvals (6 months), recruitment (2 years), follow-up of all patients (2 years per patient) and completion of data collection and analysis (6 months)

## Eligibility Criteria

Patients must meet all of the inclusion criteria and none of the exclusion criteria to be eligible for this trial. **It is important that there will be no exceptions (exemptions or waivers) made to these eligibility requirements at the time of enrolment**.

All enquiries about eligibility should be addressed as soon as possible and prior to enrolment by contacting [SOCRATES_HCC@trog.com.au](mailto:SOCRATES_HCC@trog.com.au).

### Inclusion criteria

1. Histological or radiological diagnosis of single, new HCC with largest diameter ≤8 cm (BCLC^[[1]](#footnote-2)^ stage 0 or A).
   1. If prior history of HCC, the prior HCC must have been:
      - Early stage, solitary HCC, ≤5 cm in size **and**,
      - Have arisen within a different liver segment to the current HCC **and,**
      - Treated with curative intent therapy >2 years prior with no evidence of activedisease at the site.
2. As per local multidisciplinary HCC meeting consensus patient is suitable for percutaneous thermal ablation and/or transarterial therapies **and** not suitable for or declined liver resection **and** not planned for liver transplantation.
3. Child-Pugh score ≤B7^*^ with no or diuretic-controlled ascites
4. ECOG performance status ≤2^[[2]](#footnote-3)^
5. Platelets ≥50x10^9^/L, Haemoglobin ≥80 g/L, Neutrophils ≥1.0x10^9^/L, INR <1.8 (except if on therapeutic anticoagulation)
6. 18 years of age or older and able to provide written consent

### Exclusion criteria

1. Presence of multifocal HCC, macrovascular invasion or extrahepatic disease
2. Prior treatment for any HCC within last 2 years.
3. Clinically evident ascites or hepatic encephalopathy
4. Prior abdominal radiation therapy that would preclude the delivery of protocol defined SABR to the tumour.
5. Untreated Hepatitis B or C
6. Known additional invasive malignancy (excluding non-melanoma skin cancer) that is progressing or required treatment within the last 2 years.
7. Pregnancy

# SCREENING

## Screening Log

Appropriately qualified personnel at the Trial Site will screen patient health records for potentially suitable patients according to the eligibility criteria in the protocol. The Trial Site will then record de-identified information relating to each patient screened on the screening log (including in-eligible patients); screening will continue until the target population is achieved. The screening log will be requested by the Trial Coordinating Centre on a regular basis for the purpose of monitoring accrual.

## Informed Consent

Patients must be given the current ethically approved participant information sheet for their consideration. Patients should only be asked to consent to the study after they have had sufficient time to consider the trial and the opportunity to ask any further questions. Any study specific procedures can only be performed after the participants has consented to participate.

Patients will also be asked to separately consent to the study team accessing their Medicare data for PBS and MBS funded health care service use.

## Participation in other research

Enrolment of a participant into another treatment intervention study is not permitted prior to confirmed disease progression.

Enrolment in unrelated non-interventional studies is discouraged but will be considered on a case by case basis by the Trial Management Committee. Any study that could impact on the primary endpoint of the SOCRATES study is not permitted.

## Methods for assigning treatment groups

The appropriate delegated staff member will determine the patients eligibility (see Section 4.3 Eligibility Criteria) prior to randomisation.

### Cohort selection

Each participant will be allocated into a cohort based on the following criteria;

- Patients with HCC ≤ 3 cm deemed **eligible** for percutaneous thermal ablation by the local MDT^[[3]](#footnote-4)^ will be allocated to Cohort 1
- Patients with HCC >3cm or deemed **ineligible** for percutaneous thermal ablation by the local MDT will be allocated to Cohort 2

### Randomisation Treatment Allocation

Participant will be randomised within each cohort;

- Participants in Cohort 1 will be randomly allocated (1:1) to receive either SABR verses percutaneous thermal ablation.
- Participants in Cohort 2 will be randomly allocated (1:1) to receive SABR verses SOC therapies
  *Note: SOC therapies include transarterial therapies (TACE or TARE) and/or MWA/RFA (alone or in combination).*

All randomisations will be completed through a web-based, password-protected randomisation system. Once the investigator has confirmed eligibility, the randomisation details are to be entered into the system by suitably delegated site personnel. The eligibility criteria will be reviewed by trial management staff prior to randomsiation. A confirmation of randomisation and treatment allocation will be emailed to site personnel, including the principal investigator.

Randomisation for both cohorts will be performed using block sizes of 4 to help ensure minimal imbalance in the size of treatment groups for each cohort.

Participants in each cohort will be stratified for;

1. Child-Pugh classification (A vs. B7)
2. BCLC stage (0 vs. A ; i.e. maximum tumour diameter ≤2 cm vs. >2cm)

Participants can only be randomised once in this trial.

## Blinding

Participant and treating clinicians will not be blinded to the treatment assignment after the randomisation process. Given the distinct post-treatment imaging appearances of the different therapies it is also not feasible to reliably blind the local radiologists or central independent radiological review to treatment assignment.

Data collectors and analysers will be blinded to treatment assignment

# TREATMENT INTERVENTIONS

All primary therapy interventions are to commence within **28 days of randomisation** and must be completed within **42 days of randomisation**. This time window allows for additional percutaneous thermal ablation procedures (if initial ablation incomplete) or for combination therapy (eg. TACE-RFA for HCC >3cm) as part of the “first line” standard of care therapy.

## Percutaneous thermal ablation

### Cohort 1 Participants

- Technique (MWA or RFA) will be as per local departmental protocols and delivered as per local practice.
  - Patients undergoing irreversible electroporation or laparoscopic assisted ablations are not eligible for the study.
  - Relative contraindications for percutaneous thermal ablation include tumours <10mm from the gallbladder, hepatic flexure or stomach, or central bile ducts.
  - Hydro-dissection to protect adjacent structures, eg. bowel, is permitted.
- MWA or RFA will utilise CT, Cone-beam CT or ultrasound guidance aiming for an ablation margin of >5mm.
- A contrast-enhanced CT should be performed to verify ablation margins within 24 hours (refer to section 6.1.1.1 for further details).
- In the event of initial incomplete ablation, further attempts at complete ablation are recommended within the primary therapy timeframe. This will be considered part of the initial course of primary treatment.
- All primary therapy procedures including repeat ablations must be completed **within 42 days of randomisation**.

#### Quality Assurance (QA) measures for MWA/RFA in Cohort 1

- A post-ablation contrast enhanced CT is required to verify the ablation margin.
  This is recommended to occur within 24 hours post ablation and includes;
  - Immediate post-ablation contrast enhanced CT in the case of cone-beam CT or CT guidance
  - Contrast enhanced CT within 24 hours of US guided ablation^[[4]](#footnote-5)^

If there are concerns regarding adequacy of the margin repeat contrast enhanced CT or MRI imaging may be considered within 4 weeks of ablation.

- Central collection of initial baseline (pre-randomisation abdominal imaging scans as per **Table *5*: Schedule of Assessments**) and post-ablation imaging confirming complete ablation (and ablation margin), in DICOM format will be required.
  - Central review of the post-ablation contrast enhanced CT and ablation margin estimate will be conducted for each centre’s first case and sampling 1 in 5 thereafter. Additional sampling may be undertaken as determined by the central review team.
  - Submission of treatment details (including thermal ablation technique, estimation of minimum and maximum ablation margin) will also be required and should be collected via the SOCRATES Percutaneous Ablation Worksheet.
  - The post-ablation imaging data and treatment details for participants sampled for central review should be submitted to TROG within 2 weeks of the date of the ablation.
- Please refer to the SOCRATES HCC Quality Assurance (QA), Planning and Delivery Guidelines for further details.

### Cohort 2 participants

- MWA or RFA may be used in cohort 2 for tumours >3cm if this represents standard of care therapy at the institution. It may be combined with transarterial therapies. All therapy is required to be completed within the primary therapy window of within 42 days from randomisation.
- MWA or RFA is not allowed for tumours ≤3cm in cohort 2 as this is a specific exclusion criteria for cohort 2. These patients are eligible for cohort 1.
- Technique will be as per local departmental protocols.

#### QA Measures for MWA or RFA in cohort 2

- Collection of treatment reports and mode of ablation (MWA v RFA, CT v USS-guided)
- Immediate post-ablation imaging and margin assessment will be as per departmental protocols and will not be assessed centrally for cohort 2.
  - Please see **APPENDIX 3: Imaging Guidelines** for recommendations on specific imaging sequences.

## Transarterial therapy

Transarterial therapy this may include TACE or TARE as per local practice.

- TARE should aim to deliver a mean tumour dose of ≥120Gy.

### QA measures for TACE/TARE in cohort 2

- Collection of treatment reports.
- TACE
  - Document TACE technique eg cTACE, DEB TACE, B-TACE (conventional or bead)
  - Document chemotherapy agent/s used.
  - Document size of DEBs used.
- TARE
  - Document predicted mean dose to tumour on the basis of Tc-MAA scintigraphy and liver volume studies.
  - Document dosimetry method
    - BSA, partition, modified partition, voxel-based dosimetry.
  - Document software package/s used.
  - Document actual tumour dose achieved on basis of post-TARE PET-CT if possible.

## Stereotactic Ablative Body Radiotherapy (SABR)

Radiation therapy (RT) treatment details are provided in the SOCRATES HCC Quality Assurance (QA), RT Planning and Delivery Guidelines. The QA, RT Planning and Delivery Guidelines must be used as the primary source for planning and delivering radiotherapy treatment within the trial.

### Pre-trial Quality Assurance (QA) Program

All centres participating in this study must have completed pre-trial RT QA components **prior to enrolling patients** to the study.

- - - 1. Evidence of prior SABR Phantom Dosimetry Audit (conducted by an external independent auditing body)
      2. Completion of SABR Facility Questionnaire including Liver SABR Process and Motion Management Module
      3. Submission of benchmarking case if not previously performed for abdominal SABR technique*

**Additional credentialing activities may be required as deemed necessary by the Study team.*

Please refer to the SOCRATES HCC QA, Planning and Delivery Guidelines for further details on the RT QA program.

### RT Treatment Prescription and Schedule

SABR must commence within 28 days of randomisation and be completed within 42 days of randomisation. The local investigator has discretion over the prescription dose and fractionation schedule, within the following ranges stipulated. The fraction schedules permitted are as follows:

**Table 3: Recommended SABR Dosing Schedules**

| **CRITERIA** | **DOSE** | **FRACTIONS** | **BED_10_** |
| --- | --- | --- | --- |
| CP-A no dose limiting OAR* | 50 or  42-45 | 5  3 | 100  100-112 |
| CP-B7 no dose limiting OAR* | 40-50  36 | 5 (preferred)  3 | 72-100  79 |
| Dose limiting OAR* | 35-40 | 5 | 60-72 |

*Including meeting liver dose constraints.

The aim is to deliver the highest possible biological dose to the planning target volume (PTV ) from the protocol defined dose schedules, whilst adhering to the organ at risk (OAR) constraints. For participants with well compensated liver function (CP-A) and with tumours not abutting dose limiting organs, a schedule delivering ≥100 Gy BED10 is recommended. This corresponds to 42-45Gy in 3 fractions or 50Gy in 5 fractions delivered to ≥95% of the PTV.

Treatment fractions are recommended to be delivered on 2-3 non-consecutive days per week, over a period of ≤ 15 days. Delays and treatment gaps should be avoided, however if gaps occur, please refer to the SOCRATES HCC QA, RT Planning and Delivery Guidelines for further information.

### Dose Criteria

Target volume and OAR dose constraints must be adhered to. The planning target goal is for the prescription isodose to encompass at least 95% of the PTV and 99% of the GTV/ITV. Please refer to the SOCRATES HCC QA, RT Planning and Delivery Guidelines for further details.

### Target Volume Definitions

Target volumes must be defined as per ICRU 50, 62 and 83, with clear definitions, individual contouring and specific labeling^(53-55)^. Please refer to the SOCRATES HCC QA, RT Planning and Delivery Guidelines for details.

### Motion Management

A motion management strategy must be utilised for all participants. Various motion management strategies can be used to minimise treatment volumes and reduce dose to normal tissue, and should be selected based on treatment site and participant suitability. Please refer to the SOCRATES HCC QA, RT Planning and Delivery Guidelines for details.

### Treatment Delivery

Treatment must be delivered using a conformal or intensity modulated technique including DCAT, IMRT, VMAT, TomoTherapy or CyberKnife. Beam arrangements, energies, dose rates and other specific approaches are at the discretion of the treating centre. Please refer to the SOCRATES HCC QA, RT Planning and Delivery Guidelines for details.

### Treatment Verification

All patients must undergo daily pre-treatment online imaging verification. Verification imaging must be capable of visualising the target (or surrogate) with soft tissue matching as well as bone alignment. This would necessitate on-board imaging capable of 3-dimensional representation of soft tissue. Fiducial tracking is also acceptable. Please refer to the SOCRATES HCC QA, RT Planning and Delivery Guidelines for details.

### On-Trial Radiotherapy Quality Assurance (RT QA) Program

This trial includes an on-trial RT QA program which consists of the following:

1. Phase 1 Sampling:
   - Pre-Treatment SABR QA Technical Review
   - Post-Treatment (pre-RT sampled) SABR QA Technical Review
2. Phase 2 Sampling:
   - Post-Treatment SABR QA Technical Review

Please refer to the SOCRATES HCC QA, RT Planning and Delivery Guidelines.

## Concomitant and other treatments

Participants may receive any medication that is clinically indicated for treatment of adverse events. All concomitant treatments given, with reasons for the treatment, will be recorded in the CRF.

Information on any concomitant medications and treatment in the 4 weeks prior to randomisation will be recorded.

Prior to confirmation of disease progression (or study withdrawal/completion), subsequent regimens of anti-cancer therapy or other investigational drugs should not be used. After disease progression confirmed, only subsequent regimens of anti-cancer therapy will be recorded in CRF.

## Treatment post progression

After per-protocol progression has been confirmed by central radiological review (please refer to Section 8 DISEASE PROGRESSION and Section 15.7.4 Progression Reviews for further details), treatment will be according to local institutional standard of care. Crossover therapy is permitted.

Where expedited central review is required, the treating team should contact [qa@trog.com.au](mailto:qa@trog.com.au) to discuss timelines for data submission and central review.

# TRIAL PARTICIPANT ASSESSMENTS

Patients must provide written, informed consent (Section 5.2 Informed Consent) before any study specific procedures occur.

## Screening

Consenting patients will be assessed to ensure that they meet the trial eligibility criteria (Section 4.3 Eligibility Criteria). Subjects who do not meet these criteria must not be enrolled into the study.

All screening procedures are to be completed as per **Table 5: Schedule of Assessments** and within 28 days of randomisation (unless stated below) and include;

- Demographic and characteristics (including gender and indigenous status)
- Documentation of eligibility for MWA/RFA or TACE/TARE.
- Documentation of Child Pugh status and BCLC staging
- Documentation of viral hepatitis B and C status and treatment
- Medical/surgical history including prior anti-cancer therapies
- Clinical Assessment including;
  - ECOG Performance status
  - Presence of ascites or encephalopathy
  - Weight, height and body mass index
- Concomitant medications (from 4 weeks prior to consent)
- Patient reported outcomes (EORTC QLQ-C30, QLQ-HCC18)
- Disease assessment
  - Abdominal Imaging: Multiphase contrast-enhanced CT or MRI of the liver (See APPENDIX 3: Imaging Guidelines for recommended sequences)
  - Chest imaging: CT chest
- Blood tests:
  - Clinical chemistry: AST, ALT, ALP, GGT, total bilirubin, albumin, creatinine, sodium
  - Haematology: Haemoglobin (Hgb), White Cell Count (WCC), Neutrophil count (Neuts), Lymphocyte count (Lymph), Platelet count (Plts)
  - Coagulation: INR
  - AFP

Once screening procedures have been conducted and the patient’s eligibility has been confirmed, the patients may be randomised into the study (see section **5.4.2 Randomisation Treatment Allocation**).

Treatment interventions are to be commenced within 28 days of randomisation and be completed within the primary therapy window (not more than 42 days from randomisation).

## End of Treatment

To be performed on the last day +/- 2 days of SABR treatment or prior to discharge following each standard of care therapy.

- Clinical assessment including;
  - ECOG Performance status
  - Adverse event reporting (CTCAE v5.0)
  - Concomitant medications
- For the thermal ablation treatments in cohort 1, a post-ablation contrast enhanced CT is required to verify the ablation margin.
  - This is recommended to occur within 24 hours post ablation (see section 6.1.1.1 Quality Assurance (QA) measures for MWA/RFA)

Note: A summary of staff resources used in the planning and delivery/conduct of treatment will be submitted in to the trial database at the End of Treatment visit (as described in **section 14.6.1 Health economics analysis**)

## Follow-up

### 4 weeks Post Treatment (+/- 1 week)

- Blood tests:
  - Clinical chemistry: AST, ALT, ALP, GGT, total bilirubin, albumin, creatinine, sodium
  - Haematology: Hgb, WCC, Neuts, Lymph, Plts
  - Coagulation: PT, APTT, INR
  - AFP
- Clinical assessment including;
  - ECOG Performance status
  - Presence of ascites or encephalopathy and Child Pugh score
  - Weight and body mass index
  - Adverse event reporting (CTCAE v5.0)
    - *Assessment of toxicity is continuous and will include any event occurring between assessments*.
  - Concomitant medications
  - Recording of any additional investigations or events performed or hospitalisations (required for Health economics analysis)
- Patient reported outcomes (QLQ-C30, QLQ-HCC18)
- Invitation for the participant to take part in a telephone interview to talk about their treatment experience in the trial with verbal confirmation of continuing consent
  - If consented, the interview will be conducted by a central designated interviewer within 2 weeks.
  - See **section 10 QUALITATIVE REVIEW OF THE PATIENT EXPERIENCE** for further details.

### Post Randomisation

Visits will occur from 3 months post-randomisation in 3 monthly intervals (+/- 2 week) to 2 years and then 6 monthly (+/- 2week) until completion of the trial and are to include;

- Blood tests:
  - Clinical chemistry: AST, ALT, ALP, GGT, total bilirubin, albumin, creatinine, sodium
  - Haematology: Hgb, WCC, Neuts, Lymph, Plts
  - Coagulation: PT, APTT, INR
  - AFP
- Clinical assessment including:
  - ECOG Performance status
  - Presence of ascites or encephalopathy and Child Pugh score
  - Weight and body mass index
  - Adverse event reporting (CTCAE v5.0)
    - *Assessment of toxicity is continuous and will include any event occurring between assessments*.
  - Concomitant medications
  - Recording of any additional investigations or events performed or hospitalisations (required for Health economics analysis)
- Disease assessment:
  - Abdominal Imaging: Multiphase contrast-enhanced CT or MRI of the liver
    - In the individual patient it is recommended that the same imaging modality is used throughout the study for more reliable response assessment.
    - See **APPENDIX 3: Imaging Guidelines** for recommended sequences.
  - See **Section 8 DISEASE PROGRESSION** for further details on assessment for disease progression.

#### Patient reported outcomes

Patient reported outcomes (QLQ-C30, QLQ-HCC18) to be collected at 6 monthly intervals post-randomisation (e.g. 6, 12, 18, 21, 24, 30, 36 months).

## Progression

Once progression has been confirmed on abdominal imaging, completion of restaging with a CT chest (+/- pelvis) is required within 2 weeks.

On-trial assessments should continue beyond progression to allow for collection of subsequent therapies, QoL, and second sites of progression to aid primary and secondary endpoint evaluations. It is expected that the use of further locoregional therapies for new sites of intrahepatic disease relapse outside the original target lesions will be a common event over the course of study.

## Survival status

Survival status will be obtained by the site personnel, when requested periodically by the trial management team, by contacting the participant, checking the participant's hospital records contacting the participant's treating clinician or general practitioner and/or checking publicly available death registries.

## Schedule of Assessments

**Table 4: Schedule of Assessments**

| **ASSESSMENT** | **PRE**  **RANDOMISATION** |  | **END OFTREATMENT** | **FOLLOW UP** | | | | | | | | | | **PROGRESSION^12^** |
| --- | --- | --- | --- | --- | --- | --- | --- | --- | --- | --- | --- | --- | --- | --- |
|  |  |  |  | **Post Treatment** | **Post**  **Randomisation** | | | | | | | | |  |
|  |  |  |  | **4 wks** | **3**  **mo** | **6**  **mo** | **9**  **mo** | **12**  **mo** | **15**  **mo** | **18**  **mo** | **21**  **mo** | **24**  **mo** | **30+**  **mo ^1^** |  |
| **Eligibility confirmation** | ✓ |  |  |  |  |  |  |  |  |  |  |  |  |  |
| **Informed consent** | ✓ |  |  |  |  |  |  |  |  |  |  |  |  |  |
| **Medical History** | ✓ |  |  |  |  |  |  |  |  |  |  |  |  |  |
| **Clinical assessment^2^** | ✓ |  | ✓ | ✓ | ✓ | ✓ | ✓ | ✓ | ✓ | ✓ | ✓ | ✓ | ✓ |  |
| **Concomitant Medications^4^** | ✓ |  | ✓ | ✓ | ✓ | ✓ | ✓ | ✓ | ✓ | ✓ | ✓ | ✓ | ✓ |  |
| **Bloods^5^** | ✓ |  |  | ✓ | ✓ | ✓ | ✓ | ✓ | ✓ | ✓ | ✓ | ✓ | ✓ |  |
| **Hepatitis status^6^** | ✓ |  |  |  |  |  |  |  |  |  |  |  |  |  |
| **Abdominal Imaging ^7,8^** | ✓ |  |  |  | ✓ | ✓ | ✓ | ✓ | ✓ | ✓ | ✓ | ✓ | ✓ |  |
| **Chest Imaging^9^** | ✓ |  |  |  |  |  |  |  |  |  |  |  |  | ✓ |
| **Pregnancy Test^10^** | ✓ |  |  |  |  |  |  |  |  |  |  |  |  |  |
| **Patient Reported Outcomes^11^** | ✓ |  |  | ✓ |  | ✓ |  | ✓ |  | ✓ |  | ✓ | ✓ |  |
| **Adverse Event Reporting**  **(CTCAE v5)** |  |  | ✓ | ✓ | ✓ | ✓ | ✓ | ✓ | ✓ | ✓ | ✓ | ✓ | ✓ |  |
| **Participant experience interview** |  |  |  | ✓**^13^** |  |  |  |  |  |  |  |  |  |  |

**KEY;**

| 1 | Subsequent follow up every 6 months thereafter until study completion |  |
| --- | --- | --- |
| 2 | To include: ECOG Performance status, presence of ascites or encephalopathy, weight, body mass index |  |
| 3 | To confirm absence of uncontrolled ascites or encephalopathy only (ECOG, weight, BMI not required) |  |
| 4 | After disease progression confirmed, only subsequent regimens of anti-cancer therapy will be recorded |  |
| 5 | Includes FBC (Hb, platelets, WCC, neutrophils, lymphocytes), INR, PT/APTT, AST, ALT, ALP, GGT, total bilirubin, albumin, creatinine, sodium and AFP |  |
| 6 | Documentation of hepatitis B & C virological status and need for treatment |  |
| 7 | Multiphase contrast-enhanced CT or MRI of the liver. See Appendix 3 Imaging Guidelines for recommended CT and MRI sequences. |  |
| 8 | For thermal ablation treatments in cohort 1 only, a contrast enhanced CT is required to verify the ablation margin within 24 hours of ablation. See Section 6.1.1 Quality Assurance (QA) measures for MWA/RFA |  |
| 9 | CT chest (+/- pelvis) |  |
| 10 | Women with childbearing potential only. Testing as per institutional protocol. | |
| 11 | EORTC QLQ-C30, QLQ-HCC18 |  |
|  |  |  |
| 12 | Follow-up should continue beyond progression to allow for collection of subsequent therapies, QoL, and second sites of progression |  |
| 13 | If consented, the interview will be conducted by a central designated interviewer within 4 weeks of treatment completion. |  |

# DISEASE PROGRESSION

Participants will undergo regular tumour assessment (multiphase contrast-enhanced CT or MRI of the liver) as per **Table 5: Schedule of Assessments** until disease progression, withdrawal, end of trial, or death, whichever occurs first. See **APPENDIX 3: Imaging Guidelines** for recommendations on specific imaging sequences.

- Baseline tumour measurements must be undertaken prior to any protocol treatment commencing. (see **Figure 2: mRECIST assessment of tumour lesions at baseline** for target lesion definition). The initial assessment of response will be determined after all protocol treatment has been completed, irrespective whether all of the planned treatment was delivered.
- The target lesion is to be assessed by mRECIST and RECIST v1.1 criteria (see sections **8.1 Definition of Progression** and **8.2 Definitions of (non-PD) target lesion** responses and **Figure 3: mRECIST assessment of tumour response**) at each applicable follow-up timepoint until progression.

Local progression is defined as at the first timepoint that either mRECIST **or** RECIST v1.1 criteria for progression are met. In addition, for cohort 1 (thermal ablation verses SABR) local progression will include the development of any new lesions within 10mm of the ablation cavity following thermal ablation or within 15mm of the target lesion following SABR.

In the event of a progression event, or if the investigator is in doubt as to whether progression has occurred, the imaging should be submitted for central radiological review and no further therapy undertaken until unequivocal progression has been confirmed. Repeat imaging at the next scheduled assessment, or sooner if clinically indicated, is recommended for equivocal findings. If subsequent repeat imaging confirms unequivocal progression the date of the initial imaging will be used as the date of progression. Please refer to section 14.7.4 for further details.

## Definition of Progression

### Local progression

Target lesion progression will be assessed by mRECIST and RECIST v1.1. If arterial phase enhancement cannot be used then RECIST v1.1 criteria for progression will apply (see **Table 7: Summary of Response Assessment**).

- For **typical** lesions, local progression is defined by evaluation of the target lesion as measured by mRECIST criteria, i.e. an increase of ≥20% (compared to nadir) in longest diameter of viable arterial enhancing tumour with an absolute increase of ≥5mm, excluding intervening areas of complete necrosis. (43)

The longest viable tumour diameter may or may not be located in the same scan plane in which the baseline longest viable tumour diameter was measured.

- For **atypical** lesions (non-arterially enhancing or rim-enhancing at baseline) or for those lesions with equivocal or discordant central review mRECIST findings, progression according to RECIST v1.1 criteria will also be scored as a local progression event, i.e. an increase of ≥20% (compared to nadir) in longest diameter of the whole target lesion with an absolute increase of ≥5mm. (56)

Equivocal findings or discordant central radiological assessments should be confirmed with interval imaging and additional therapy should not be undertaken until unequivocal progression has been confirmed. Equivocal findings are expected to be more common for the radiation-based therapies (TARE and SABR).

Additionally, for Cohort 1, local progression will include any recurrence within 10mm of the ablation zone for MWA/RFA or within 15mm of the target lesion for SABR.(23) For SABR treatments this will equate to a >10mm margin on the high dose region, i.e the planning target volume (PTV) which itself is a ≥5mm margin on the target lesion. Given there can be significant parenchymal atrophy and contraction around the SABR treated lesion with distortion of liver shape, a direct overlay of the PTV will not accurately depict the treated region over time. Similarly, it is acknowledged that a larger thermal ablation margin or in the case of SABR, the use of larger PTVs or development of larger volumes of off-target parenchymal contraction introduces some variability to this spatial definition.

**Table 5: Definition of Local Progression by Cohort**

| **COHORT AND TREATMENT** | | **BASELINE MEASUREMENT** | **LOCAL PROGRESSION DEFINITION** |
| --- | --- | --- | --- |
| **Cohort 1**  **Comparison** | **MWA/RFA** | Ablation Cavity* | Within 10mm of ablation cavity |
|  | **SABR** | Target lesion | Within 15mm of target lesion |
| **Cohort 2**  **Comparison** | **SOC** | Target lesion | Target lesion |
|  | **SABR** | Target lesion | Target lesion |

*For RECIST V1.1 measurement

### New Lesions

New intrahepatic and extrahepatic lesions are defined as per mRECIST criteria.

- For extrahepatic progression mRECIST criteria excludes ascites and pleural effusion unless cytologically confirmed but otherwise retains the RECIST v1.1 criteria for measurable disease.

### Summary of Response Assessment

**Table 6: Summary of Response Assessment**

| **mRECIST** | **RECIST v1.1** |
| --- | --- |
| **Primary Target Lesion**  **Typical lesion**: ≥ 20% increase in diameter of viable (arterially enhancing) tumour from nadir with absolute increase of ≥5 mm, excluding intervening areas of necrosis.  **Atypical lesion**: measure as per RECIST v1.1 | **Target lesion**  If equivocal or discordant review findings on mRECIST then a ≥ 20% increase in diameter of target lesion from nadir with absolute increase of ≥5 mm as per RECIST v1.1. will be scored as a local progression event. |
| **New Intrahepatic Lesions**  **Typical lesion**: new lesion ≥1cm  **Atypical lesion**: requires increase in size of nodular lesion by ≥ 1cm on serial imaging.  **Macrovascular invasion**: new arterially enhancing vascular thrombus. |  |
| **Extrahepatic Lesions**  As per RECIST v1.1 with the exception that new or progressing ascites and pleural effusion are excluded unless cytologically confirmed and peri-portal lymph nodes should be ≥20mm short axis. | **Extrahepatic Lesions**  Any lesion that is detected at a given post-baseline timepoint and has no corresponding lesion on baseline imaging and is unequivocally malignant will be considered as evidence of disease progression.  Small or equivocal lesions should be reassessed with interval imaging. |

## Definitions of (non-PD) target lesion responses

- Complete Response (CR)
  - mRECIST: The disappearance of any intratumoural arterial enhancement in the target lesion
  - RECIST v1.1: The disappearance of the target lesion (including all lymph node short axis diameters regressed to normal, i.e., short axis that shrinks to <1 cm)
- Partial Response (PR)
  - mRECIST: At least a 30% decrease in the longest viable tumour diameter of target lesion from baseline
  - RECIST v1.1: At least a 30% decrease in the pertinent diameter of the target lesion, from baseline
- Stable Disease (SD)
  - mRECIST: The disappearance of any intratumoural arterial enhancement in the target lesion
  - RECIST v1.1: The disappearance of the target lesion (including all lymph node short axis diameters regressed to normal, i.e., short axis that shrinks to <1 cm)

## Management of Disease progression

See **Section 6.5 Treatment post progression**.

# PATIENT REPORTED OUTCOMES

Patient reported outcomes (PRO), an umbrella term referring to all outcomes and symptoms are directly reported by the participant. PROs have become a significant endpoint when evaluating the effectiveness of treatments in clinical trials. The following PROs will be administered:

## EORTC QLQ-C30

The EORTC QLQ-C30 consists of 30 items and measures cancer patients’ functioning and symptoms for all cancer types.(48) Questions can be grouped into 5 multi-item functional scales (physical, role, emotional, cognitive and social); 3 multi-item symptom scales (fatigue, pain, nausea and vomiting); a 2-item global health status scale; 5 single items assessing additional symptoms commonly reported by cancer patients (dyspnoea, loss of appetite, insomnia, constipation, diarrhoea) and 1 item on the financial impact of the disease.

## EORTC QLQ-HCC18

The EORTC QLQ-HCC is an 18 item instrument designed for use in parallel with the QLQ-C30 core instrument to assess all major dimensions of health-related quality of life in patients with HCC.(50, 51) The instrument is an 18-item scale, consisting of six symptom scales and two single items: fatigue (three items), body image (two items), jaundice (two items), nutrition (five items), pain (two items), fever (two items), sexual interest (one item), and abdominal swelling (one item). Scores are based on a 4-point Likert scale (with 1 = ‘not at all’ to 4 = ‘very much’); scaled scores for each domain ranged from 0–100 with a higher score indicating worse symptoms.

## Non-English speaking patients

This study may include participants that cannot read or write English. In this situation, one of the following options may be implemented in order to include these participants in the PRO component of the study:

1) The questionnaire will be supplied in the participants language

2) If the questionnaire is not available in the participants language, the assessment tool for that time point will not be given to the participant to complete.

The information regarding the collection of the PRO data will be documented on the Patient Reported Outcome (PRO) Completion and Missing Data (CoMiDa) checklist (Section 9.4 Patient Reported Outcome Completion and Missing Data).

## Patient Reported Outcome Completion and Missing Data

The Patient Reported Outcome (PRO) Completion and Missing Data (CoMiDa) checklist is a document developed by the Quality of Life office within the Psycho-oncology Co-operative Research Group (PoCoG)

The PRO CoMiDa checklist is a data management tool, designed to provide standardised documentation of the completion or reasons for non-completion of QOL assessments by participants in a clinical trial/study. Such documentation is crucial for quality assurance since missing data is the greatest threat to the integrity and interpretability of QOL data.

A PRO CoMiDa checklist has been included in the Case Report Form (CRF) suite for this trial. The person responsible for data collection at the site (i.e. Site Trial Coordinator, Data Manager, Research Nurse, or equivalent) must complete the PRO CoMiDa checklist at each scheduled time point.

## Completion of Patient Reported Outcome questionnaires

It is vital that the questionnaires completed within acceptable time limits surrounding the scheduled assessments (see **Table 5: Schedule of Assessments**). The completion rates of QoL forms will be monitored closely by the TCC. Specific instructions regarding the administration of PRO questionnaires are provided in the Data Management manual for this trial. These instructions must be followed to ensure the wellbeing of trial participants and the validity of PRO data. Please contact the TCC for further details.

# QUALITATIVE REVIEW OF THE PATIENT EXPERIENCE

A qualitative analysis of the participants’ perceptions of the treatments delivered during the trial is helpful to compliment the quantitative, efficacy outcomes of the trial. The primary aim of this qualitative review will be to investigate participants’ treatment experience with respect to overall treatment tolerability and satisfaction and how it varies according to the specific treatment received (SABR, percutaneous ablation, TACE). The data will assist with differentiating the participant treatment experience with different modalities, beyond other secondary outcomes such as adverse events.

An inductive, descriptive qualitative study design with qualitative interviews has been chosen.

## Number of participants

Sixty patients will be consecutively recruited over the duration of the study, with 15 patients from each of the four anticipated main treatments (SBRT, TACE, percutaneous ablation, combined TACE and percutaneous ablation).

## Recruitment and consent process

Participants will be invited to take part in the telephone interview by a delegated site staff member (e.g. their site study coordinator) within 4 weeks of treatment completion.

At this visit the delegated site staff member will;

- Inform the participant that;
  - the interview will take approximately 20 minutes
  - will involve briefly talking about their treatment experience in the trial
  - will be conducted by an interviewer from Flinders Medical Centre (South Australia), who is independent researcher from who is not part of the treatment or chief investigator research team
  - it is their choice to accept this invitation or not and if they consent, that they can change their mind and cancel the interview if they wish without effecting their continual care
- Obtain written consent from the participant for the designated interviewer to contact them for participation in the qualitative interviews and for site staff to pass on contact details to the interviewer.
- Site staff will pass on the name, study ID and contact details of the consented participant to the designated interviewer. These details WILL NOT be shared with the trial CRA or the chief investigator research team).

## Data collection

Designated interviews will conducted the patient interviews and these researchers will not be part of the treatment or chief investigator research team.

All interviews will be;

- Conducted within 4 weeks of treatment completion.
- Conducted over the phone
- Audio-recorded and transcribed verbatim, and field notes will be taken during and after each interview
- Open-ended and carried out as a conversation to understand the participant's treatment experience, with respect to tolerability and satisfaction, within the trial
  - An interview script with a set of standardised questions will be provided to the interviewer for consistency between interviews (see **APPENDIX 5: Qualitative Review of the patient experience of non-operative treatment in early-stage hepatocellular carcinoma: Example interview script**).

It is anticipated that each interview will take approximately 20 minutes to complete.

## Data Storage

Recordings of interviews will be allocated a unique study ID prior to being sent for transcription.

All data and digital recordings and will be stored securely, in a password protected computer and only the study investigators will have access to the data.

Participants will be given the option of requesting the transcript to check their answers.

## Analysis

A grounded theory approach will be used to analyse the data. This approach for content analysis is interpretive in nature and used to describe or illuminate a phenomenon through identification of manifest (the obvious) and latent (underlying meaning) content in a text. This will involve:

- - 1. reading of the text several times to become familiar with it and reflect upon the content,
    2. identification in the text of meaning units that describe the phenomenon,
    3. meaning units condensed and essential content is abstracted and labelled with a code, and
    4. codes compared based on similarities and differences sorted into themes. Coding and sorting of themes will be assisted using the analysis tool NVivo.

# WITHDRAWAL FROM TREATMENT OR WITHDRAWAL FROM STUDY

At any time, participants are free to discontinue study treatment or withdraw from the study without prejudice to further treatment.

- Where applicable, the participant will be asked about the reason(s) for discontinuing study treatment or withdrawing from the study.
- Where possible, the investigator will perform the best possible observation(s), test(s) and evaluation(s) as well as provide appropriate medication and all give possible measures for the safety of the participant. Ongoing adverse events will be assessed by an investigator and followed, either during scheduled study follow-up or, in the case of withdrawal of consent, outside of the clinical study.

The trial coordinating centre is to be notified of the discontinuation of study treatment and/or withdrawal from the study as soon as possible.

The status of ongoing, withdrawn (from the study) and “lost to follow up” participants at the time of an overall survival analysis should be obtained by the site personnel by checking the participant notes, hospital records, contacting the participant or their general practitioner and checking publicly available death registries.

## Discontinuation of a participant from treatment

Participants may be discontinued from treatment in the following situations:

- Participant decision
- Adverse event
- Severe non-compliance with the study protocol as judged by the investigator and/or TMC.
- Participant incorrectly initiated on study treatment.
- If the treating investigator believes the participant is no longer receiving clinical benefit from treatment and/or requires additional anti-cancer treatment

Study procedures related to SAEs and anti-cancer treatment must be captured until permanent withdrawal from the study.

Participants who stop study involvement prior to the time recommended in the protocol will be requested to continue follow-up visits according to the protocol.

If a participant wishes to stop the study visits, they will continue to receive ongoing care as per their treating team. Study investigators may request an update regarding their ongoing health status via their medical records unless the subject has withdrawn consent.

## Withdrawal from Study

The term withdrawal from the study refers to both discontinuation from study treatment AND study assessments.

Reasons for withdrawal from the study:

- Death
- Withdrawal of consent

If a participant wishes to withdraw their consent to both treatment and study assessments, they should be asked if they are willing to continue with survival follow up (which can be conducted by telephone).

Regardless of the reason for withdrawal, all data available for the participant at the time of discontinuation of follow up must be recorded. All reasons for discontinuation of treatment must be documented.

If a participant wishes to withdraw their consent to further participation in the study entirely, including survival follow up, this should be clearly documented in the participant notes.

## Participant transfers

Every effort should be made for a trial participant moving from the area to continue their treatment and/or follow-up at another participating Trial Site and for this Trial Site to take over responsibility for the participant.

Until the new Trial Site formally agrees (in writing) to take over responsibility and provides evidence of ethics approval, the participant remains the responsibility of the original Trial Site. Source documentation will remain at the original Trial Site up to the date of transfer.

# TERMINATION OF THE STUDY

The study may be stopped if in the judgement of the Sponsor and TMC, if trial participants are placed at undue risk because of clinically significant findings that:

- Meet individual stopping criteria or otherwise considered significant.
- Are assessed as causally related to study treatments.
- Are not considered to be consistent with the continuation of the study.

Should the study be terminated, the Sponsor will ensure that adequate consideration is given to the protection of the participants’ interests.

# SAFETY REPORTING AND MEDICAL MANAGEMENT

## Definitions

**Adverse Event (AE):** An AE is any untoward medical occurrence (toxicity, sign, symptom, abnormal result on an investigation or disease temporally associated with the use of a medicinal product) in a patient administered a study treatment; the event does not necessarily have a causal relationship with the treatment.

The term AE is used to include both serious and non-serious AEs.

**Serious Adverse Event (SAE):** An SAE is any untoward medical occurrence that occurs between consent and 28 days after cessation of treatment interventions, that fulfils one or more of the following criteria:

- Results in death
- Is immediately life-threatening (an event in which the participant was immediately at risk of death *at the time of event*)
- Requires in-patient hospitalisation or prolongation of existing hospitalisation
- Results in persistent or significant disability/incapacity
- Is a congenital anomaly/birth defect
- Important medical event (events may be considered a serious adverse experience if they require medical or surgical intervention to prevent one of the listed definitions, e.g. an ‘allergic bronchospasm’ which required intensive treatment in an emergency room or at home)

An event will not be considered to be a SAE if;

- Hospitalisation is due to administration of trial procedures
- Hospitalisation is due to placement of a permanent intravenous catheter
- Hospice placement for terminal care is required
- Hospitalisation is due to pre-trial scheduled elective surgery
  - Out-patient hospitalisation for procedures such as:
  - Elective day surgery
- Convenience purposes (e.g. transportation difficulties)
- Death is due to cancer (this is considered ‘expected’ and is therefore not a SAE)
- Any new cancer is diagnosed that does not meet the SAE criteria (this is to be reported on relevant CRF).

## Adverse event (AE) reporting

### Adverse Events (AE)

Adverse events are to be reported between from consent and 28 days after all the last study treatment [list study treatments] is discontinued and must be graded according to The National Cancer Institute (NCI) Common Terminology Criteria for Adverse Events (CTCAE) version 5.0 by a delegated staff member. For each AE, the highest grade observed since the last visit should be reported on the relevant CRF.

All non-serious adverse events (including anticipated and unanticipated device related adverse events) shall be recorded in the trial database via adverse event CRFs. Internal statistical analysis of these data shall be performed at the times specified in the protocol and investigators and responsible ethical committees will be advised of any safety issues which emerge during this process.

Any AEs that are unresolved at the participant’s last visit in the study are followed up by the investigator for as long as medically indicated, but without further recording in the CRF. The TMC retains the right to request additional information for any participant with ongoing AE(s) at the end of the study, if judged necessary.

### Serious Adverse Events (SAE)

SAEs are to be reported to the TCC by the trial site within 24 hours of being notified, from consent and within 28 days from the completion of treatment [SABR, RFA, MWA, TARE, TACE] is completed/discontinued.

The investigator at the trial site is responsible for;

- grading the event according to the [NCI CTCAE vs 5](chrome-extension://efaidnbmnnnibpcajpcglclefindmkaj/https:/ctep.cancer.gov/protocoldevelopment/electronic_applications/docs/ctcae_v5_quick_reference_5x7.pdf)
- identifying the seriousness of the adverse event
- identifying the causal relationship to the treatment (see **Table 8: Definitions of causality**)
  - attribution to the SAE must be recorded in the patients’ medical records and reported on the SAE form
- identifying the causal relationship in relation to other medication
- determining the expectedness according to the Investigators Brochure (medicinal products)
- reporting to their local approving authorities as per local guidelines
- to follow-up the event until resolution or death

The initial SAE report should always include at least the following information; participant number, suspected medicinal produce if applicable, identifiable reporting source, description of medical event, seriousness criteria and causality assessment by the investigator. Remaining information must be forwarded to the TCC as soon as possible, but no later than 7 calendar days from the initial report.

The investigator is also responsible for notifying responsible HRECs, Research Governance Officers or other appropriate regulatory authorities of the SAE according to local ethical and regulatory guidelines. They are also to follow up the event (with regular reporting) until resolved.

The trial coordinating centre (TCC) shall;

- Implement and maintain a suitable recording system to record information from all SAEs received from Trial Sites.
- On receipt of an SAE, arrange for the trial chairpersons and/ or a clinical reviewer to review the event and confirm causal relationship and expectedness
- Report all SAEs to TROG Cancer Research
- Notify the sponsor (TROG Cancer Research) and all other participating investigators of any serious unanticipated related adverse events and any action taken
- Notifying the regulatory authorities of each country of any significant issue that has arisen from analysis of overseas reports or action that has been taken by another country’s regulatory authority in reporting timelines.

**Table 7: Definitions of causality**

| **RELATIONSHIP** | **DESCRIPTION** |
| --- | --- |
| Unrelated | There is no evidence of any causal relationship with the trial treatment |
| Unlikely | There is little evidence to suggest there is a causal relationship (e.g. the event did not occur within a reasonable time after administration of the trial treatment). There is another reasonable explanation for the event (e.g. the patient’s clinical condition, other concomitant treatment) |
| Possible | There is some evidence to suggest a causal relationship (e.g. because the event occurs within a reasonable time after administration of the trial treatment). However, the influence of other factors may have contributed to the event (e.g. the patient’s clinical condition, other concomitant treatments) |
| Probable | There is evidence to suggest a causal relationship, and the influence of other factors is unlikely |
| Definitely | There is clear evidence to suggest a causal relationship, and other possible contributing factors can be ruled out |

## New cancers

The development of a new non-HCC cancer should be regarded as an AE, or an SAE if they meet at least one of the serious criteria. New non-HCC cancers are those that are not the primary reason for the administration of the study treatment and have been identified after the participant’s inclusion in this study. They do not include metastases of the original cancer.

# STATISTICAL CONSIDERATIONS

## Sample Size Calculation

**Cohort 1:** HCC ≤3cm **and** eligible for percutaneous thermal ablation.

- From a review of the literature, we estimate a 2 year FFLP of 75% in the thermal ablation arm. A 20% difference in 2 year FFLP was considered clinically significant for this surrogate endpoint given the potential for local salvage and multiple subsequent lines of therapy. Assuming a 2-sided type 1 error rate of alpha=0.05, 47 evaluable patients per arm will provide 80% power to detect an absolute difference in 2 year FFLP of 20% (95% SABR versus. 75% thermal ablation). The required total sample size of 47+47=94 will be inflated to N=47/0.80=59 per arm to allow for a drop-out rate of 10% and a 2-year mortality rate of 10%. The required sample size is therefore estimated N=118.

**Cohort 2:** HCC >3cm **or** ineligible for percutaneous thermal ablation if ≤3cm.

- For the “treatment stage migration” cohort, we estimate a 2-year FFLP for the SOC therapies of 65%. Assuming a 2-sided type 1 error rate of alpha=0.05, a sample size of 40 patients per arm will provide 80% power to detect an absolute difference in event rates at 2 years of 25% (90% SABR versus 65% SOC). (16, 18, 57) Assuming a 10% drop-out rate and 10% mortality rate at 2 years the estimated number of patients per treatment arm is N=40/0.80=50 per arm (SABR or SOC). The required sample size is therefore estimated N=100.

## Sequence Generation

Randomisation for both patient groups will be performed using random block sizes of 6 and 4 to help ensure minimal imbalance in the size of treatment groups for each patient cohort.

To prevent imbalance in disease severity and HCC BCLC stage, stratified block randomisation using two factors will be used for SABR vs SOC cohorts (see **Section 5.4.2 Randomisation Treatment Allocation**).

## Endpoint Definitions

**Table 8: Endpoint definitions**

|  | **ENDPOINTS** | **MEASURES** |
| --- | --- | --- |
| **PRIMARY:** | 2-year FFLP | Local target lesion progression in Cohort 1 and Cohort 2 defined by mRECIST and RECIST v1.1 as outlined above in Section 8.1 **Definition of Progression*** |
| **SECONDARY:** | 2-year PFS | Local progression or the development of new intrahepatic or extrahepatic lesions as defined in Section 8 DISEASE PROGRESSION |
|  | 2-year OS | Death from any cause |
|  | Safety and adverse events | Cumulative incidence CTCAE v5.0 |
|  | Patient reported outcomes | EORTC QLQ-C30 and QLQ-HCC18 - mean scores over time. Time to ≥10 point decrease in the combined GHS/QoL score. |
|  | Health economic comparisons | Costs cost per QALY gained for SABR compared with PA/SOC at two years |

* If the estimated treatment effects for FFLP are similar for the 2 cohorts we will also perform an additional secondary analysis for FFLP in which data from the 2 cohorts are combined. With 218 patients and an overall event rate of 15% in the combined population, this sample size will provide 90% power to detect a hazard ratio=2.0 at alpha=0.05.

## Analysis population

The following populations will be defined:

- Intention to treat
- Per protocol

## Definition of analyses sets

### Full analysis set

The full analysis set will include data for all patients that are randomised. Where there is less than 10% of data missing, ITT analysis will be performed using mixed effects modelling and standard survival statistics without imputation. For outcomes with more than 10% of data missing, ITT analysis will be performed using multiple imputation and mixed effects models and standard survival statistics. The multiple imputation for each outcome will include all variables considered informative to the censoring mechanism for that outcome.

### Safety analysis set

The safety population will consist of the per-protocol population of patients that received their assigned treatment throughout the trial. Subjects will be included in the per protocol analysis for each outcome if they have complete data at each timepoint for that outcome. Safety data to be analysed will include adverse events, toxicity, and laboratory evaluations. The TROG Independent Data and Safety Monitoring Committee (IDSMC) will evaluate safety data every 6 months to ensure that the trial is safe to continue.

## Statistical Analysis Plan

A statistical analysis plan (SAP) will be finalised prior to datalock and contain additional detail on the methods described below.

All analyses will be performed according to an intention-to-treat protocol, with a per-protocol analysis performed as a sensitivity analysis. For the FFLP, PFS, TTP, ORR and OS endpoints, analysis will be performed comparing 2-year estimates from cumulative incidence (FFLP) and Kaplan-Meier survival curves (PFS and OS). The two comparisons between groups (SABR vs. MWA/RFA and SABR vs. SOC) will be analysed separately. Secondary analysis using Gray and log-rank tests will be performed to evaluate differences in overall time to event distributions. Fine and Gray, Cox proportional hazards regression will used to estimate hazard ratios with 95% confidence intervals. Secondary outcomes including the EORTC QLQ-C30 and QLQ-HCC18 (presented as mean scores over time) will be assessed using linear mixed-effects models to account for repeated measures. Additionally, time to deterioration (TTD) of combined GHS/QoL will be analysed. TTD will be defined as the time to first onset of a ≥10 point decrease from baseline. A 2-sided type-1 error rate of alpha=0.05 will be used for all hypothesis testing. All analysis will be performed using Stata software (version 17).

### Health economics analysis

This study uses a cost-effectiveness analysis to investigate whether the use of SABR represents value for money relative to SOC. The assessment of costs will reflect the use of SABR and its associated comparators, as well as ongoing patient management costs for HCC care (including physician visits, diagnostic and monitoring services, and the management of treatment relative toxicities). Primary hospital service use for the delivery of SABR and conduct of SOC treatments will be recorded through case report form reporting, noting the mode of delivery for SABR, the form of SOC undertaken and whether or not patients received TACE prior to MWA/RFA. Ongoing treatment costs will be assessed using administrative data (Medicare data) for outpatient medical service use (MBS) and pharmaceutical services (PBS). Patient specific costs associated with accessing care, particularly those associated with travel time for attending clinics for care, will also be assessed based on a patient completed questionnaire. In addition, out-of-pocket costs for MBS and PBS use will be estimated based on Medicare data reporting. For each patient, the costs of care will be assessed at the end of follow-up.

Outcomes for the cost-effectiveness analysis include the difference in the proportion of patients with FFLP at two years, and the difference in quality adjusted life years (QALYS) observed over the study period. In the first instance, outcomes will be expressed based on the primary outcome, the two-year FFLP rate, and the cost-effectiveness expressed as the cost per additional patient with local control at two years. QALYs, the combined impact of the impact of HCC and its treatment on survival and quality of life, will be assessed using patient completed information from the EORTC QLQ-C30. Thus, responses to that questionnaire, will be converted to values on a preference scale – 0 to 1 and applied to survival time to estimate QALYs, allowing the cost per QALY gained for SABR compared with SOC at two years to be estimated.

The base case analysis will adjust for the effect on OS of crossover post-progression. The potential for costs and outcomes to extend beyond the duration of the trial, and the resulting impact on the cost per QALY, will be explored in a modelled analysis. Cost-effectiveness will be estimated for each of the patient cohorts separately (PA eligible and stage migration patients, respectively) and for an overall weighted analysis based on the proportion of early inoperable HCC patients in Australia anticipated to fall into these two groups.

## Secondary Analysis

TROG encourages secondary analysis and TROG Policy Statement E12 *Secondary Analyses in TROG Clinical Trials* outlines the procedures for this.

There is an intent to undertake and facilitate ad hoc secondary analyses with data collected during the trial.

Participants on this study will be asked to grant advanced permission for the possible future sharing of the data collected in this study. Additional participant consent for use of the data will only be sort if it is a requirement of the HREC.

# STUDY AND DATA MANAGEMENT

## Participating Centres

The Principal Investigator at each centre should comply with all the terms, conditions, and obligations of the Clinical Trial Research Agreement (CTRA), or equivalent, for this study. In the event of any inconsistency between this Clinical Study Protocol and the CTRA, the terms of Clinical Study Protocol shall prevail with respect to the conduct of the study and the treatment of participants and in all other respects, not relating to study conduct or treatment of participants, the terms of the CTRA shall prevail.

Before activating the trial, participating sites are required to sign an agreement accepting responsibility for all trial activity which takes place within their site.

Once the CTRA has been signed by all required signatories, the required trial documentation is in place and a site initiation (visit or teleconference) has taken place, notification of site activation will be issued to the PI and other applicable site staff.

**Sites must not approach any potential participant until they have received the activation notification.**

## Training of study site personnel

Before the first participant is entered into the study, training will be provided on the requirements of the Clinical Study Protocol and related documents with the investigational staff and training in any study specific procedures.

The Principal Investigator will ensure that appropriate training relevant to the study is provided to their study team, and that any new information relevant to the performance of this study is forwarded to the staff involved.

The Principal Investigator will maintain a record of all individuals involved in the study (medical, nursing and other staff).

### Radiotherapy

Each centre will complete radiotherapy quality assurance procedures, as described in Section 6.3 and the **SOCRATES HCC Quality Assurance (QA), RT Planning and Delivery Guidelines**, prior to commencing recruitment. The quality assurance programme will continue throughout the trial, with investigator training as required.

## Monitoring of the study

During the study, the central trial coordinator will have regular contact with the site to:

- Provide information and support to the Investigator(s)
- Confirm that facilities remain acceptable
- Confirm that the investigational team is adhering to the protocol, that data is being accurately and timely recorded in the CRFs and accountability checks are being performed

### Site visits

If a monitoring visit is required, TROG will contact the site to arrange the visit. Once a date has been confirmed, the site should ensure that full medical records of participants selected for source data verification are available for monitoring.

TROG staff conducting on-site monitoring will review essential documentation and carry out source data verification to confirm compliance with the clinical trial agreement and trial protocol. If any problems are detected during the monitoring visit, TROG will work with the Principal Investigator/s or delegated individual to resolve issues and determine appropriate action.

### Audit or inspection

Authorised representatives of the Sponsor, funder, applicable regulatory authorities, or an Ethics Committee may perform audits or inspections at the centre, including source data verification. The purpose of an audit or inspection is to systematically and independently examine all study related activities and documents, to determine whether these activities were conducted, and data were recorded, analysed, and accurately reported according to the protocol, Good Clinical Practice (GCP), guidelines of the International Conference on Harmonisation (ICH), and any applicable regulatory requirements. The Investigator will contact TROG immediately if contacted by a regulatory agency about an inspection at the centre.

### Archiving of study documents

Essential trial documents and source documentation (including medical history, radiological imaging, laboratory tests, treatment records, verification films and portal images) must be retained for at least 15 years after completion of the trial in accordance with ICH GCP Guidelines. Documents should be securely stored and access restricted to authorised personnel.

### Regulatory reviews

The TCC will be responsible for collecting, maintaining and monitoring all required essential documentation from the Trial Site before, during and after the conduct of the trial.

## Study timetable and end of study

The end of the study is defined as the last visit of the last participant undergoing the study.

The study may be terminated at individual centres if the study procedures are not being performed according to GCP, or if recruitment is slow. TROG may also terminate the entire study prematurely if concerns for safety arise within this study or from any other study with similar interventions.

## Data acquisition and management

Case Report Forms (CRF) will be used for the collection of trial data. The TCC will provide guidance to sites to aid the completion of the CRFs.

The TMC reserves the right to amend or add to the CRF template suite as appropriate. Such changes do not constitute a protocol amendment, and revised or additional forms should be used by sites in accordance with the guidelines provided by the TCC.

The investigator will ensure that the data are recorded in the Case Report Forms as specified in the study protocol and in accordance with the instructions provided. The investigator ensures the accuracy, completeness and timeliness of the data recorded and of the provision of answers to data queries according to the Clinical Study Agreement. The investigator will sign the completed Case Report Form at the end of the study.

## Security and back up of data

All study data will be stored securely either in locked rooms or on a secure server at trial site and the TCC. Access to the study data will be restricted to trial staff. In addition, Site Trial Coordinators will only have access to their own centre’s data. At the TCC a password system will be utilised to control access to the trial database and only those staff members who will be working on this study will be allocated access to this trial. All reports prepared will be prepared such that no individual participant can be identified.

An independent external back-up and disaster recovery system that ‘mirror replicates’ a copy of all data saved in real-time is in place at the TROG Central Operations Office. The back-up server is located off-site in a climate-controlled facility and will be retained indefinitely.

## Study Conduct Quality Assurance reviews

### Regulatory Reviews

The Trial Coordinating Centre will be responsible for collecting, maintaining and monitoring all required essential documentation from the Trial Site before, during and after the conduct of the trial.

### Informed Consent

Reviews of de-identified consent forms will be performed for all cases to determine that Informed Consent was obtained before registration on the trial.

Throughout the trial, copies of relevant documents (such as clinical history notes, pathology/histology reports, imaging reports, blood test results) may be requested for source data verification.

### Eligibility Reviews

The eligibility criteria for entry into the trial will be reviewed by the sponsor (TROG) for all participants.

### Progression Reviews

Disease progression image sets (DICOM) and supplementary data will be uploaded to the TROG Central Quality Management System (CQMS) for all participants where a disease progression event has been triggered, or if the investigator is in doubt as to whether progression has occurred. Please contact [QA@trog.com.au](mailto:QA@trog.com.au) for login and CQMS upload instructions. Further details can also be found in the SOCRATES HCC Quality Assurance (QA), Planning and Delivery Guidelines.

NOTE: CQMS will de-identify the participant scan on upload. Sites are responsible for de-identifying the imaging report and any additional supporting documentation prior to submission.

# RESEARCH GOVERNANCE

## Sponsor and Funder

This trial is sponsored by the Trans Tasman Radiation Oncology Group (TROG) and funded by NHMRC Medical Research Future Fund (Rare Cancers and Unmet Needs) grant. This funding source will have no role in the design or execution of this study, analyses, interpretation of the data, or decision to submit results.

As the sponsor of this trial, TROG requires that all TROG Policy Statements regarding the study design; collection, management, analysis, interpretation of data, writing of the report and publications are adhered to by the Trial Chairperson.

## Trial Chairpersons

The Trial Chairpersons have the overall responsibility for the design and conduct of the trial according to TROG Policy Statements. Further details regarding the responsibilities and delegations are set out in the Clinical Trial Agreement between TROG and the Trial Chairpersons.

## Trial Executive Committee and the Trial Management Committee

The Trial Executive Committee (TEC) / Trial Management Committee (TMC) will be responsible for the monitoring of the progress of the trial, decision making, education and information services and reporting as described in TROG Policy Statement TPS E8 Trial Management Committee Responsibilities. TEC members are listed at the beginning of the protocol.

## Independent Data Safety Monitoring Committee

The TROG Independent Data Safety Monitoring Committee (IDSMC), separate to the TMC, will independently monitor the conduct of the trial in order to ensure its ethical and scientific integrity.

The roles and responsibilities of the IDSMC are set out in TROG Policy Statement TPS E9 Data Monitoring Committee Guidelines and throughout this protocol document.

## Principal Investigator

In each participating centre, a Principal Investigator will be identified. They will be responsible for the trial conduct at the centre (including satellite sites) as per ICH GCP guidelines and as set out in the Clinical Trial Agreement between TROG and the participating centre. Each Principal Investigator is to adhere to the TROG Policy Statements and is strongly encouraged to be a current member of TROG.

## Consumer Representative

TROG encourages wider participation from consumer representatives’, as defined by the Statement on Consumer and Community Participation in Health and Medical Research, and supports the recommendation that consumers are involved in decision making throughout the development, conduct and closeout phases of a trial.

# PATIENT PROTECTION AND ETHICAL CONSIDERATIONS

## Ethical Principles and Regulatory Compliance

The study will be performed in accordance with ethical principles that have their origin in the Declaration of Helsinki and are consistent with ICH/Good Clinical Practice and conducted according to the approved protocol and its amendments, supplementary guidance and manuals supplied by the Sponsor and in accordance with relevant national guidelines.

## Adherence to Protocol

Except for an emergency situation in which proper care for the protection, safety and wellbeing of the trial participant requires that an alternative treatment be used, the trial shall be conducted exactly as per the terms and instructions described in the approved protocol. No protocol exceptions, waivers or exemptions will be granted. If the protocol constraints cannot be met then the participant should be treated off the trial.

## Cultural values and principles

TROG recognises and commits to the respect of Aboriginal, Torres Strait Islander, Maori and other indigenous populations cultural values and principles. Although this trial is not targeted specifically to Indigenous peoples, a person from one of these communities may be invited to participate if they meet the eligibility criteria of this trial.

The invitation to a participant in the trial will be at the discretion of the Investigator at the Trial Site. The investigator will consent and treat the participant according to the principles set forth in the applicable country’s guidelines (examples listed below) and any specific requirements of the approving authority.

**Table 9: Cultural principles**

| **COUNTRY** | **CULTURAL GUIDELINES** | **APPROVING AUTHORITY** |
| --- | --- | --- |
| Australia | Guidelines for Ethical Conduct in Aboriginal and Torres Strait Islander Health Research | Human Research Ethics Committees (HREC) |

## Confidentiality

The trial will be conducted in accordance with applicable Privacy Acts and Regulations. All information regarding trial participants must be treated in strict confidence. Data, which identify any trial participant, must not be revealed to anyone not directly involved in the trial or the clinical care of that participant. An exception is where the trial participant has provided written consent for his/her records to be included in source document verification. In this instance, the records may be inspected by (a) a representative of TROG for the purposes of source document verification or quality audit as stipulated in the ICH GCP Guidelines, or (b) a representative of a government regulatory authority for the purposes of official inspection. Records must be made available for inspection on the understanding that all information relating to trial participants will be treated in strict professional confidence.

## Informed consent

The Investigator(s) at each centre will:

- Ensure each participant is given full and adequate oral and written information about the nature, purpose, possible risk and benefit of the study.
- Ensure each participant is notified that they are free to discontinue from the study at any time.
- Ensure that each participant is given the opportunity to ask questions and allowed time to consider the information provided.
- Ensure each participant provides signed and dated informed consent before conducting any procedure specifically for the study.
- Ensure the original, signed Informed Consent Form(s) is/are stored in the Investigator’s Study File.
- Ensure a copy of the signed Informed Consent Form is given to the participant
- Ensure that any incentives for participants who participate in the study as well as any provisions for participants harmed as a consequence of study participation are described in the informed consent form that is approved by an Ethics Committee.

## Insurance and compensation

TROG endorses the principles of the Medicines Australia Guidelines for Compensation for Injury Resulting from Participation in a Company Sponsored Trial in Australian and the Research Medicines Industry equivalent in Singapore.

To provide protection for trial participants involved in TROG Clinical Trials, TROG maintains a clinical trials insurance policy.

# PUBLICATION AND PRESENTATION POLICY

The Trial Chairs and TMC are responsible for presentations and publications arising from this trial with the TROG Publications Committee providing oversight and independent scientific review of all relevant material prior to submission.

Refer to TROG Authorship, Publication and Spokesperson Guideline for detailed information and responsibilities of both committees. Authorship will be determined via the guidelines and by recruitment of site.

# REFERENCES

1. Global, regional, and national progress towards Sustainable Development Goal 3.2 for neonatal and child health: all-cause and cause-specific mortality findings from the Global Burden of Disease Study 2019. Lancet. 2021;398(10303):870-905.

2. Petrick JL, Braunlin M, Laversanne M, Valery PC, Bray F, McGlynn KA. International trends in liver cancer incidence, overall and by histologic subtype, 1978-2007. Int J Cancer. 2016;139(7):1534-45.

3. Wallace MC, Preen DB, Short MW, Adams LA, Jeffrey GP. Hepatocellular carcinoma in Australia 1982-2014: Increasing incidence and improving survival. Liver Int. 2019;39(3):522-30.

4. Welfare AIoHa. Cancer in Australia 2019. Cancer series no.119. Cat. no. CAN 123. 21 Mar 2019 ed. Canberra: AIHW; 2019.

5. European Association for the Study of the Liver. Electronic address eee, European Association for the Study of the L. EASL Clinical Practice Guidelines: Management of hepatocellular carcinoma. J Hepatol. 2018;69(1):182-236.

6. Reig M, Forner A, Rimola J, Ferrer-Fábrega J, Burrel M, Garcia-Criado A, et al. BCLC strategy for prognosis prediction and treatment recommendation Barcelona Clinic Liver Cancer (BCLC) staging system. The 2022 update. J Hepatol. 2021.

7. Marrero JA, Kulik LM, Sirlin CB, Zhu AX, Finn RS, Abecassis MM, et al. Diagnosis, Staging, and Management of Hepatocellular Carcinoma: 2018 Practice Guidance by the American Association for the Study of Liver Diseases. Hepatology. 2018;68(2):723-50.

8. Devaki P, Wong RJ, Marupakula V, Nangia S, Nguyen L, Ditah IC, et al. Approximately one-half of patients with early-stage hepatocellular carcinoma meeting Milan criteria did not receive local tumor destructive or curative surgery in the post-MELD exception era. Cancer. 2014;120(11):1725-32.

9. Loo K, Woodman RJ, Bogatic D, Muller KR, Chandran V, Chinnaratha MA, et al. Treatment stage migration for early HCCs and its impact on outcomes- a multi-centre study Hepatology. 2020;72(S1):655A-A.

10. Roberts SK, Gazzola A, Lubel J, Gow P, Bell S, Nicoll A, et al. Treatment choice for early-stage hepatocellular carcinoma in real-world practice: impact of treatment stage migration to transarterial chemoembolization and treatment response on survival. Scand J Gastroenterol. 2018;53(10-11):1368-75.

11. Zhang YJ, Chen MS, Chen Y, Lau WY, Peng Z. Long-term Outcomes of Transcatheter Arterial Chemoembolization Combined With Radiofrequency Ablation as an Initial Treatment for Early-Stage Hepatocellular Carcinoma. JAMA Netw Open. 2021;4(9):e2126992.

12. Lu DS, Raman SS, Limanond P, Aziz D, Economou J, Busuttil R, et al. Influence of large peritumoral vessels on outcome of radiofrequency ablation of liver tumors. J Vasc Interv Radiol. 2003;14(10):1267-74.

13. Kuvshinoff BW, Ota DM. Radiofrequency ablation of liver tumors: influence of technique and tumor size. Surgery. 2002;132(4):605-11; discussion 11-2.

14. Jeong Y, Lee KJ, Lee SJ, Shin YM, Kim MJ, Lim YS, et al. Radiofrequency ablation versus stereotactic body radiation therapy for small (</= 3 cm) hepatocellular carcinoma: A retrospective comparison analysis. J Gastroenterol Hepatol. 2021.

15. Chinnaratha MA, Sathananthan D, Pateria P, Tse E, MacQuillan G, Mosel L, et al. High local recurrence of early-stage hepatocellular carcinoma after percutaneous thermal ablation in routine clinical practice. Eur J Gastroenterol Hepatol. 2015;27(3):349-54.

16. Baek MY, Yoo JJ, Jeong SW, Jang JY, Kim YK, Jeong SO, et al. Clinical outcomes of patients with a single hepatocellular carcinoma less than 5 cm treated with transarterial chemoembolization. Korean J Intern Med. 2019;34(6):1223-32.

17. Ishikawa K, Chiba T, Ooka Y, Suzuki E, Ogasawara S, Maeda T, et al. Transarterial chemoembolization as a substitute to radiofrequency ablation for treating Barcelona Clinic Liver Cancer stage 0/A hepatocellular carcinoma. Oncotarget. 2018;9(30):21560-8.

18. Sapir E, Tao Y, Schipper MJ, Bazzi L, Novelli PM, Devlin P, et al. Stereotactic Body Radiation Therapy as an Alternative to Transarterial Chemoembolization for Hepatocellular Carcinoma. Int J Radiat Oncol Biol Phys. 2018;100(1):122-30.

19. Salem R, Johnson GE, Kim E, Riaz A, Bishay V, Boucher E, et al. Yttrium-90 Radioembolization for the Treatment of Solitary, Unresectable HCC: The LEGACY Study. Hepatology. 2021;74(5):2342-**52.**

20. Doyle A, Gorgen A, Muaddi H, Aravinthan AD, Issachar A, Mironov O, et al. Outcomes of radiofrequency ablation as first-line therapy for hepatocellular carcinoma less than 3 cm in potentially transplantable patients. J Hepatol. 2019;70(5):866-73.

21. Gory I, Fink M, Bell S, Gow P, Nicoll A, Knight V, et al. Radiofrequency ablation versus resection for the treatment of early stage hepatocellular carcinoma: a multicenter Australian study. Scand J Gastroenterol. 2015;50(5):567-76.

22. Kim JE, Kim YS, Rhim H, Lim HK, Lee MW, Choi D, et al. Outcomes of patients with hepatocellular carcinoma referred for percutaneous radiofrequency ablation at a tertiary center: analysis focused on the feasibility with the use of ultrasonography guidance. Eur J Radiol. 2011;79(2):e80-4.

23. Kim TH, Koh YH, Kim BH, Kim MJ, Lee JH, Park B, et al. Proton beam radiotherapy vs. radiofrequency ablation for recurrent hepatocellular carcinoma: A randomized phase III trial. J Hepatol. 2021;74(3):603-12.

24. Ng KKC, Chok KSH, Chan ACY, Cheung TT, Wong TCL, Fung JYY, et al. Randomized clinical trial of hepatic resection versus radiofrequency ablation for early-stage hepatocellular carcinoma. Br J Surg. 2017;104(13):1775-84.

25. Nishikawa H, Kimura T, Kita R, Osaki Y. Radiofrequency ablation for hepatocellular carcinoma. International Journal of Hyperthermia. 2013;29(6):558-68.

26. Vietti Violi N, Duran R, Guiu B, Cercueil JP, Aubé C, Digklia A, et al. Efficacy of microwave ablation versus radiofrequency ablation for the treatment of hepatocellular carcinoma in patients with chronic liver disease: a randomised controlled phase 2 trial. Lancet Gastroenterol Hepatol. 2018;3(5):317-25.

27. Takayama T, Hasegawa K, Izumi N, Kudo M, Shimada M, Yamanaka N, et al. Surgery versus Radiofrequency Ablation for Small Hepatocellular Carcinoma: A Randomized Controlled Trial (SURF Trial). Liver Cancer. 2021.

28. Dobrzycka M, Spychalski P, Rostkowska O, Wilczyński M, Kobiela P, Grąt M, et al. Stereotactic body radiation therapy for early-stage hepatocellular carcinoma - a systematic review on outcome. Acta Oncol. 2019;58(12):1706-13.

29. Rim CH, Kim HJ, Seong J. Clinical feasibility and efficacy of stereotactic body radiotherapy for hepatocellular carcinoma: A systematic review and meta-analysis of observational studies. Radiother Oncol. 2019;131:135-44.

30. LaCouture TA, Xue J, Subedi G, Xu Q, Lee JT, Kubicek G, et al. Small Bowel Dose Tolerance for Stereotactic Body Radiation Therapy. Semin Radiat Oncol. 2016;26(2):157-64.

31. Hara K, Takeda A, Tsurugai Y, Saigusa Y, Sanuki N, Eriguchi T, et al. Radiotherapy for Hepatocellular Carcinoma Results in Comparable Survival to Radiofrequency Ablation: A Propensity Score Analysis. Hepatology. 2019;69(6):2533-45.

32. Kim N, Cheng J, Jung I, Liang J, Shih YL, Huang WY, et al. Stereotactic body radiation therapy vs. radiofrequency ablation in Asian patients with hepatocellular carcinoma. J Hepatol. 2020;73(1):121-9.

33. Kubo K, Kimura T, Aikata H, Takahashi S, Takeuchi Y, Takahashi I, et al. Long-term outcome of stereotactic body radiotherapy for patients with small hepatocellular carcinoma. Hepatology Research. 2018;48(9):701-7.

34. Liu HY, Lee Y, McLean K, Leggett D, Hodgkinson P, Fawcett J, et al. Efficacy and Toxicity of Stereotactic Body Radiotherapy for Early to Advanced Stage Hepatocellular Carcinoma - Initial Experience From an Australian Liver Cancer Service. Clin Oncol (R Coll Radiol). 2020;32(10):e194-e202.

35. Mathew AS, Atenafu EG, Owen D, Maurino C, Brade A, Brierley J, et al. Long term outcomes of stereotactic body radiation therapy for hepatocellular carcinoma without macrovascular invasion. Eur J Cancer. 2020;134:41-51.

36. Park S, Jung J, Cho B, Kim SY, Yun S-C, Lim Y-S, et al. Clinical outcomes of stereotactic body radiation therapy for small hepatocellular carcinoma. Journal of Gastroenterology and Hepatology. 2020;35(11):1953-9.

37. Wahl DR, Stenmark MH, Tao Y, Pollom EL, Caoili EM, Lawrence TS, et al. Outcomes After Stereotactic Body Radiotherapy or Radiofrequency Ablation for Hepatocellular Carcinoma. J Clin Oncol. 2016;34(5):452-9.

38. Yoon SM, Kim SY, Lim YS, Kim KM, Shim JH, Lee D, et al. Stereotactic body radiation therapy for small (≤5 cm) hepatocellular carcinoma not amenable to curative treatment: Results of a single-arm, phase II clinical trial. Clin Mol Hepatol. 2020.

39. Kim N, Kim HJ, Won JY, Kim DY, Han KH, Jung I, et al. Retrospective analysis of stereotactic body radiation therapy efficacy over radiofrequency ablation for hepatocellular carcinoma. Radiother Oncol. 2019;131:81-7.

40. Shanker MD, Liu HY, Lee YY, Stuart KA, Powell EE, Wigg A, et al. Stereotactic radiotherapy for hepatocellular carcinoma: Expanding the multidisciplinary armamentarium. J Gastroenterol Hepatol. 2021;36(4):873-84.

41. Wigg AJ, Narayana SK, Le H, Iankov I, Chinnaratha MA, Tse E, et al. Stereotactic body radiation therapy for early hepatocellular carcinoma: a retrospective analysis of the South Australian experience. ANZ J Surg. 2019;89(9):1138-43.

42. Zhang T, Sun J, He W, Li H, Piao J, Xu H, et al. Stereotactic body radiation therapy as an effective and safe treatment for small hepatocellular carcinoma. BMC Cancer. 2018;18(1):451.

43. Llovet JM, Lencioni R. mRECIST for HCC: Performance and novel refinements. J Hepatol. 2020;72(2):288-306.

44. Llovet JM, Villanueva A, Marrero JA, Schwartz M, Meyer T, Galle PR, et al. Trial Design and Endpoints in Hepatocellular Carcinoma: AASLD Consensus Conference. Hepatology. 2021;73 Suppl 1:158-91.

45. Mendiratta-Lala M, Masch W, Owen D, Aslam A, Maurino C, Devasia T, et al. Natural history of hepatocellular carcinoma after stereotactic body radiation therapy. Abdom Radiol (NY). 2020;45(11):3698-708.

46. Mendiratta-Lala M, Masch WR, Shampain K, Zhang A, Jo AS, Moorman S, et al. MRI Assessment of Hepatocellular Carcinoma after Local-Regional Therapy: A Comprehensive Review. Radiol Imaging Cancer. 2020;2(1):e190024.

47. Serper M, Parikh ND, Thiele G, Ovchinsky N, Mehta S, Kuo A, et al. Patient-reported outcomes in HCC: A scoping review by the Practice Metrics Committee of the American Association for the Study of Liver Diseases. Hepatology. 2022.

48. Aaronson NK, Ahmedzai S, Bergman B, Bullinger M, Cull A, Duez NJ, et al. The European Organization for Research and Treatment of Cancer QLQ-C30: a quality-of-life instrument for use in international clinical trials in oncology. J Natl Cancer Inst. 1993;85(5):365-76.

49. King MT, Costa DSJ, Aaronson NK, Brazier JE, Cella DF, Fayers PM, et al. QLU-C10D: a health state classification system for a multi-attribute utility measure based on the EORTC QLQ-C30. Quality of life research : an international journal of quality of life aspects of treatment, care and rehabilitation. 2016;25(3):625-36.

50. Chie WC, Blazeby JM, Hsiao CF, Chiu HC, Poon RT, Mikoshiba N, et al. International cross-cultural field validation of an European Organization for Research and Treatment of Cancer questionnaire module for patients with primary liver cancer, the European Organization for Research and Treatment of Cancer quality-of-life questionnaire HCC18. Hepatology. 2012;55(4):1122-9.

51. Serrano D, Podger L, Barnes G, Song J, Tang B. Psychometric validation of the EORTC QLQ-HCC18 in patients with previously treated unresectable hepatocellular carcinoma. Qual Life Res. 2021.

52. Lubel JS, Roberts SK, Strasser SI, Thompson AJ, Philip J, Goodwin M, et al. Australian recommendations for the management of hepatocellular carcinoma: a consensus statement. Medical Journal of Australia. 2021;214(10):475-83.

53. ICRU. Report 50: Prescribing, recording and reporting Photon beam therapy. Journal of the International Commission on Radiation Units and Measurements. 1993;26.

54. ICRU. Report 62: Supplement to Report 50: Prescribing, recording and reporting Photon beam therapy. Journal of the International Commission on Radiation Units and Measurements. 1999;26(1).

55. ICRU. Report 83: Prescribing, recording and reporting Photon beam Intensity Modulated Radiation Therapy (IMRT). Journal of the International Commission on Radiation Units and Measurements. 2010;10(1):1-106.

56. Eisenhauer EA, Therasse P, Bogaerts J, Schwartz LH, Sargent D, Ford R, et al. New response evaluation criteria in solid tumours: revised RECIST guideline (version 1.1). Eur J Cancer. 2009;45(2):228-47.

57. Roberts SK, Gazzola A, Lubel J, Gow P, Bell S, Nicoll A, et al. Treatment choice for early-stage hepatocellular carcinoma in real-world practice: impact of treatment stage migration to transarterial chemoembolization and treatment response on survival. Scand J Gastroenterol. 2018;53(10-11):1368-75.

# APPENDIX 1: Child-Pugh score & Barcelona Clinic Liver Cancer (BCLC) staging system

## A1.1 Child-Pugh score

| **Measure** | **1 point** | **2 points** | **3 points** |
| --- | --- | --- | --- |
| Total bilirubin, μmol/L | < 34 | 34–50 | > 50 |
| Serum albumin, g/L | > 35 | 28–35 | < 28 |
| INR | < 1.7 | 1.7–2.3 | > 2.3 |
| Ascites | None | Mild (or suppressed with medication) | Moderate to severe (or refractory) |
| Hepatic encephalopathy | None | Grade I–II | Grade III–IV |

| **Points** | **Child–Pugh Class** |
| --- | --- |
| 5–6 | **A** |
| 7–9 | **B** |
| 10–15 | **C** |

## A1.2 Barcelona Clinic Liver Cancer (BCLC) staging system

| **Stage** | **Description** |
| --- | --- |
| 0  (Very Early) | - Single tumour ≤2 cm - Preserved liver function*, PS0 |
| A  (Early) | - Single, or ≤3 nodules each ≤ 3 cm - Preserved liver function*, PS0 |
| B  (Intermediate) | - Multinodular - Preserved liver function*, PS0 |
| C  (Advanced) | - Portal invasion and/or extrahepatic spread - Preserved liver function, PS1-2 |
| D  (Terminal) | - Any tumour burden - End stage liver function, PS 3-4 |

*Except for those with tumour burden acceptable for transplant

*As published in Journal of Hepatology; Reig M, Forner A, Rimola J, Ferrer-Fábrega J, Burrel M, Garcia-Criado Á, Kelley RK, Galle PR, Mazzaferro V, Salem R, Sangro B. BCLC strategy for prognosis prediction and treatment recommendation: The 2022 update. Journal of Hepatology. 2021 Nov 19.*

# APPENDIX 2: ECOG Performance Status

| **Grade** | **Description** |
| --- | --- |
| 0 | Normal activity. Fully active, able to carry on all pre-disease performance without restriction. |
| 1 | Symptoms, but ambulatory. Restricted in physically strenuous activity, but ambulatory and able to carry out work of a light or sedentary nature (e.g., light housework, office work). |
| 2 | In bed < 50% of the time. Ambulatory and capable of all self-care, but unable to carry out any work activities. Up and about more than 50% of waking hours. |
| 3 | In bed > 50% of the time. Capable of only limited self-care, confined to bed or chair more than 50% of waking hours. |
| 4 | 100% bedridden. Completely disabled. Cannot carry on any selfcare. Totally confined to bed or chair. |
| 5 | Dead. |
| *As published in Am. J. Clin. Oncol.: Oken MM, Creech RH, Tormey DC, Horton J, Davis TE, McFadden ET, Carbone PP: Toxicity And Response Criteria Of The Eastern Cooperative Oncology Group. Am J Clin Oncol. 1982;5:649-655. The Eastern Cooperative Oncology Group, Robert Comis*  *M.D., Group Chair.* | |

# APPENDIX 3: Imaging Guidelines

**The following is intended as a guide only**

## A3.1 Tri/quad-phasic CT protocol

Tri-phasic CT scans may be performed as either Scans 1-2-3 or Scans 2-3-4 combination. Scan 1-2-3 is required for the post-ablation scan. The addition of scan 4 is required for subsequent follow-up scans.

|  | **Scan 1**  **(Optional)** | **Scan 2** | **Scan 3** | **Scan 4** |
| --- | --- | --- | --- | --- |
| **Scan Phase/Delay** | *Pre-Contrast* | Arterial Phase  (20-25 seconds post injection or [preferably] be determined by bolus tracking) | Portal Venous Phase  (60‑70 seconds post injection) | Delayed Phase  (Min. 3 – 5 minutes post injection) |
| **Scan Location/Coverage** | Entire Liver | Entire Liver | Lung apices to symphysis pubis | Entire Liver |
| **Participant Orientation** | Supine | Supine | Supine | Supine |
| **Breath-hold Details** | Single breath-hold | Single breath-hold | Single breath-hold | Single breath-hold |
| **Scan FOV** | Large | Large | Large | Large |
| **Display FOV** | Unique to participant size | Unique to participant size | Unique to participant size | Unique to participant size |
| **Acquisition Slice Thickness** | ≤1mm | ≤1mm | ≤1mm | ≤1mm |
| **Reconstruction interval**  **(distance between slice locations)** | 3 – 5 mm  (3 mm preferred) | 3 – 5 mm  (3 mm preferred) | 3 – 5 mm  (3 mm preferred) | 3 – 5 mm  (3 mm preferred) |
| **Gap**  **(slice spacing)** | None  (i.e., contiguous) | None  (i.e., contiguous) | None  (i.e., contiguous) | None  (i.e., contiguous) |

| **Contrast Media Instructions:** | |
| --- | --- |
| **IV Contrast** | 100 – 150 mL non-ionic only (1.5 – 2 mL per kg body weight) |
| **IV Contrast Concentration** | 300 – 350 mg/mL |
| **Injection Rate** | Single Injection: 4 – 5 cc/sec via power injector |
| **Oral Contrast Option** | Use site standard protocol |

## A3.2 Triphasic MRI of the Liver

MRI sequences are heavily dependent on vendor equipment. Sites are encouraged to use their local protocol or consult with their vendor and/or specialist liver MRI radiologist to establish an optimal and reproducible protocol for diagnosing HCC.

**Scanning Technique:** the following sequences should be obtained after a standard localiser:

- **Ax** SSFSE, with an interleaved acquisition, scanned from top to bottom of liver, in a single breath hold (BH), if possible.
- **Ax** dual in-phase and out-of-phase, unenhanced BH T1 Spoiled Gradient (SPGR).
- **Ax** DWI **with** B values of 50, 400, 800 + ADC map
- **Ax** 3D Dynamic enhanced scans:

1. Pre-contrast fat saturated 3D volume acquisition.
2. MRI contrast - 0.1 – 0.2 mmol/kg, with maximum volume of 30 cc, injected at 3 cc/sec, followed immediately by a 20 cc Normal Saline flush.
3. Image acquisition, with fat saturation, should be timed to obtain early arterial, late arterial, and portal venous phase images.

# APPENDIX 4: mRECIST for Hepatocellular Carcinoma

**Figure 2: mRECIST assessment of tumour lesions at baseline**


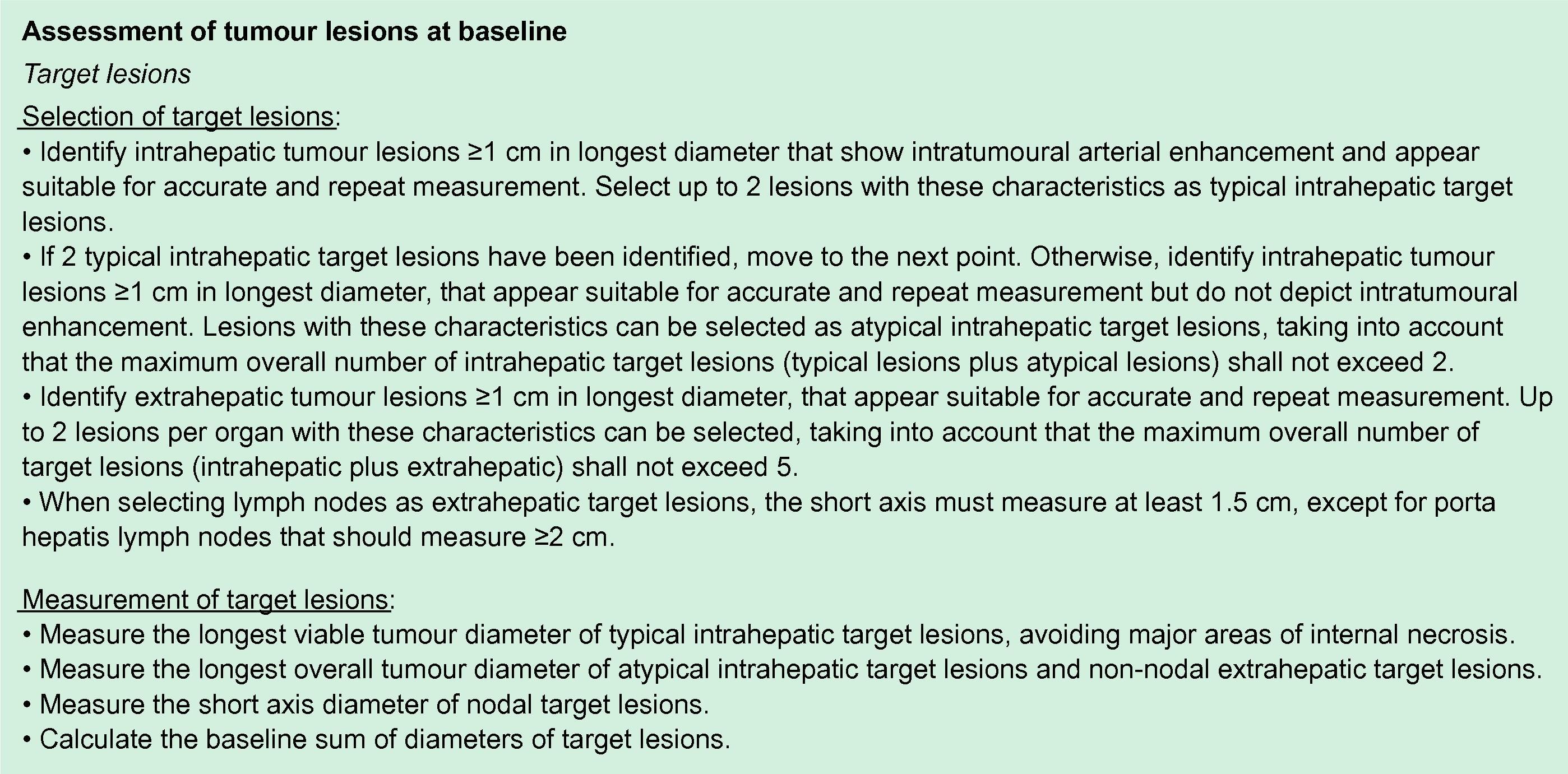


**Figure 3: mRECIST assessment of tumour response**


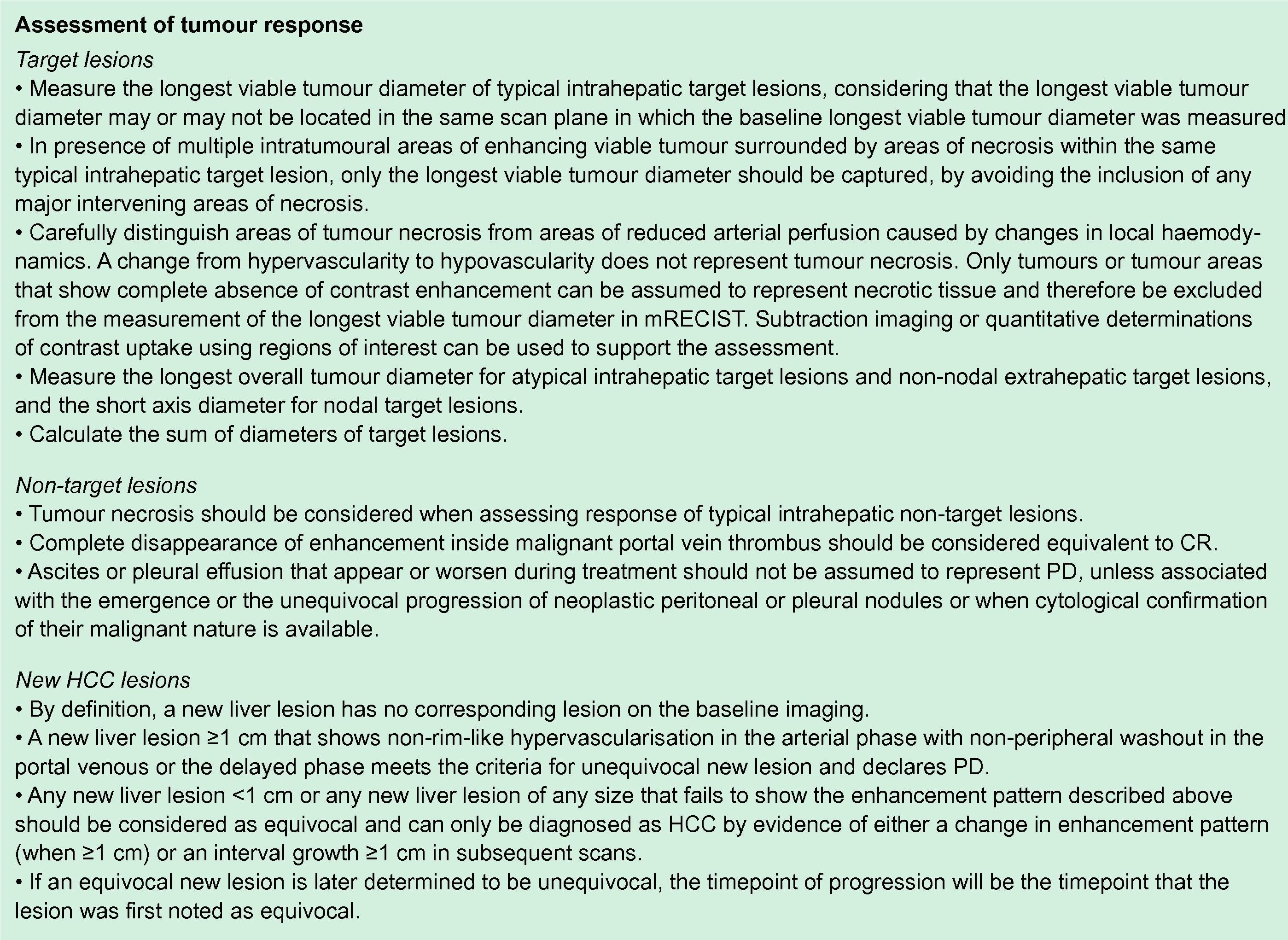


*As published in J Hepatol.:* *Llovet JM, Lencioni R. mRECIST for HCC: Performance and novel refinements. J Hepatol. 2020 Feb;72(2):288-306. doi: 10.1016/j.jhep.2019.09.026. PMID: 31954493*

# APPENDIX 5: Qualitative Review of the patient experience of non-operative treatment in early-stage hepatocellular carcinoma: Example interview script

**QUESTIONS**

Introduction Hi, my name is [state name] and I’m a researcher on the SOCRATES HCC trial from [location].

I am contacting you as you have consented to take part in a telephone interview on treatment experience. Is this still a good time for the interview?  / Thank you for taking the time to schedule an interview today.

We are looking to explore each patient’s experience, their satisfaction with their treatment experience and if this varies according to the type of treatment received (e.g SABR, RFA / MWA and/or TACE / TARE).

Your treating physician [SITE INVESTIGATOR NAME] is part of the research team conducting this research project. I would like to emphasise that I’m external to your treatment team and that if you have any questions about your treatment, you will have to direct them to your treating team. They will not be aware that you are participating in this interview.

This interview will take around 20 minutes. If at any stage you would like to stop or would prefer not to answer a question please let me know. It is important to note that the information you provide during this interview is confidential to the researchers and will not be shared in an individually identifiable manner.

This interview will be audio recorded, so I don’t have to take notes while we are talking, do you consent to this?

Do you have any questions for me before we start?

Feel free to stop and ask me questions at any time.

Questions

1. What treatment(s) did you receive for your cancer as part of the trial?
2. Tell me about your experience during the process of having XXX for your HCC treatment at your hospital, what was it like?
3. Were you satisfied with the experience of receiving XXX for your HCC treatment.
4. What were some of the good things about how the treatment was delivered?
5. Were there any bad things about the way the treatment was delivered?
6. Is there anything that would have made the experience of receiving your HCC treatment any better?
7. Do you have any other feedback about the HCC treatment you received at your hospital?

1. *See APPENDIX 1: Child-Pugh score & Barcelona Clinic Liver Cancer (BCLC) staging system* [↑](#footnote-ref-2)
2. *See APPENDIX 2: ECOG Performance Status* [↑](#footnote-ref-3)
3. MDT to consist of at least a hepatologist, a hepatobiliary surgeon and a radiologist. [↑](#footnote-ref-4)
4. *See* ***APPENDIX 3: Imaging Guidelines*** *for recommendations on specific imaging sequences.*  [↑](#footnote-ref-5)
